# Universal Approach for the Depolymerization of Polyamides via Photothermal Conversion

**DOI:** 10.1021/jacs.6c03063

**Published:** 2026-04-23

**Authors:** Deepika Shingwekar, Erin E. Stache

**Affiliations:** Department of Chemistry, 6740 Princeton University, Princeton, New Jersey 08544, United States

## Abstract

Polyamides (PAs) exhibit excellent chemical stability and mechanical resistance, yet these same characteristics lead to their widespread accumulation in the environment as pollution. In this work, we developed an inclusive and operationally simple photothermal strategy to recycle PAs, overcoming the high energy barriers necessary to break down these materials. PAs can be depolymerized using photothermally mediated ring-closing depolymerization and acidic hydrolysis to afford cyclic and linear monomers using carbon black as a photothermal agent (PTA) under visible light irradiation. We showed that polyamide 6 is efficiently depolymerized to ε-caprolactam with 74% yield in 10 min. Similarly, in 1 h, the photothermal acidic hydrolysis of polyamide 6,6 afforded hexamethylene diamine and adipic acid with 97 and 96% yields, respectively. This method was further applied to a variety of aliphatic and aromatic PAs and mixed PA waste. Both photothermally promoted processes effectively depolymerize pigment-containing postconsumer waste by leveraging existing black pigments as PTAs. Here, photothermal conversion provided a general and rapid route for PA depolymerization under visible light irradiation, enabling high monomer yields with inexpensive reagents and a general tolerance to additives, demonstrating this approach’s potential for a circular plastic economy.

## Graphical abstract

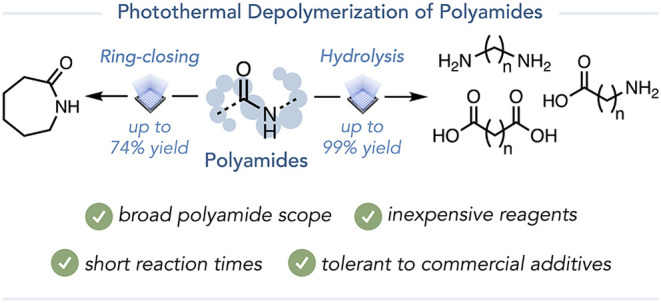

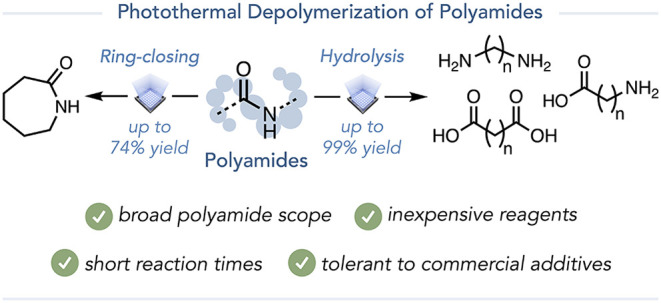

## Introduction

The widespread use of plastics has contributed to a global plastics pollution crisis, with 79% of all plastic waste ever produced ending up in landfills or leaking into the environment.^1^ The durability and ease of production have made plastics inherently essential, yet these materials often persist for decades after their use.^2^ Current plastic mitigation efforts predominantly rely on incineration for energy recovery and conventional mechanical recycling. These methods either generate undesired greenhouse gases or yield materials with inferior physical properties compared to virgin plastics, thereby necessitating the development and further improvement of recycling strategies.^3,4^ Chemical recycling has emerged as a promising approach to transform waste polymers into their constituent monomers.^5^ While this process represents a favorable strategy toward a circular plastics economy, the effectiveness of chemical recycling is highly material-dependent and often employs high bulk temperatures and designer catalysts.^6^ Resultingly, most plastics remain poorly recycled.^1^

In particular, polyamides (PAs) are a class of polymers characterized by their amide linkages in the backbone.^7^ PAs uniquely exhibit exceptional chemical resistance and mechanical strength due to the strong hydrogen bonding and resonance stabilization afforded by these characteristic backbone amide groups.^8^ Due to these advantageous properties, PAs have an extensive range of applications in textiles, automobiles, additive manufacturing, electronics, and more, with the global market share of PAs projected to increase to 48 billion USD by 2032.^9–11^ However, PA recycling is especially challenging as harsh reaction conditions are currently needed to overcome high energy barriers arising from the strong intermolecular forces between polymer chains. Consequently, the global recycling rate for PAs is <2%.^12^

Currently, several chemical recycling methods have been developed for PAs, with most efforts focused on aliphatic PAs, more commonly termed nylons (Figure 1A). Early work on polyamide 6 (PA6) recycling primarily focused on alkaline pyrolysis to recover the cyclic monomer, ε-caprolactam (CPL).^13–16^ More recently, chemical recycling efforts for PA6 have centered on solvolysis.^17–21^ Žagar and co-workers employed microwave irradiation for acidic hydrolysis to achieve near quantitative yields of monomers.^22,23^ Similarly, Yang and colleagues demonstrated that alcoholysis is a viable alternative for depolymerizing polyamides at lower temperatures.^24^ However, solvolysis methods will inevitably yield the linear open-chain monomer, 6-aminocaproic acid (ACA), which must be subsequently cyclized into CPL to be reused in polymerization. Other methods include lanthanide-catalyzed reactions and ionic liquids, yet all of these procedures employ harsh conditions and operate at temperatures above 200 °C to overcome strong interchain forces.^25–27^ Depolymerization for other PAs, including polyamide 6,6 (PA66), to obtain linear monomers, adipic acid (AA) and hexamethylenediamine (HMDA), also suffer from high reaction temperatures and extended reaction times.^23,28–32^ The vast majority of PA depolymerization methods are often limited by the materials’ high crystallinity and insolubility in most common solvents. These factors lead to the use of temperatures above the melting point of the polymer and other severe reaction conditions, necessitating further reaction development.

**Figure 1:**
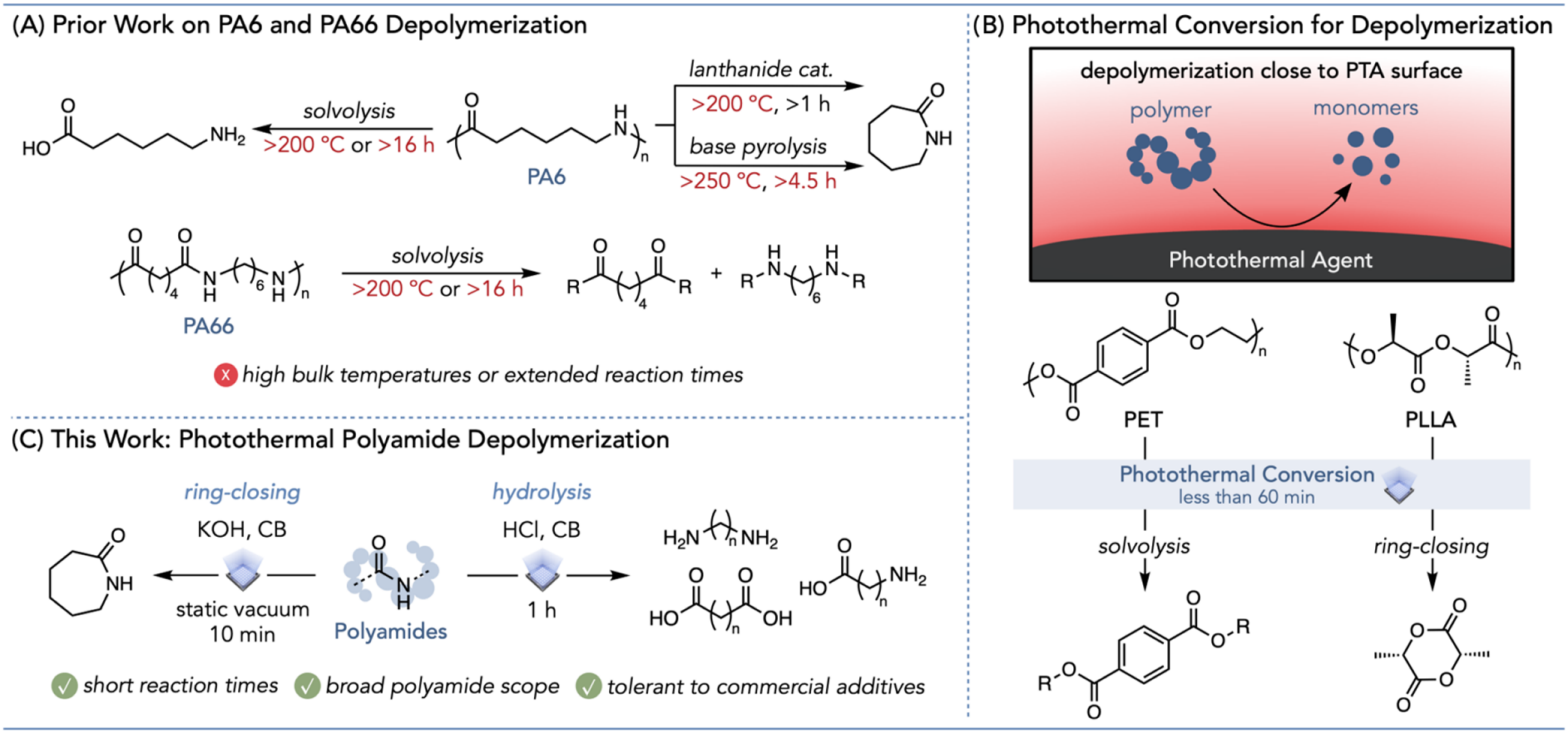
Photothermal depolymerization of polyamides. (A) Prior work on PA6 and PA66 depolymerization. (B) Photothermal conversion for depolymerization of PET and PLLA. (C) This work: Photothermal polyamide depolymerization.

As PA depolymerization often requires high temperatures and extended reaction times, photothermal conversion, a process where a large amount of heat is generated by a photothermal agent (PTA) when irradiated with visible light, could be utilized to overcome these limitations (Figure 1B).^33–35^ During irradiation, PTAs produce a localized heat gradient near the PTA’s surface, reducing unwanted side reactivity often observed when heating the bulk of the reaction.^34,36,37^ Additionally, the generation of a localized heat gradient from photothermal conversion is nearly instantaneous, allowing for short reaction times, often under 60 min.^33^ Our previous work using photothermal conversion utilizes carbon black (CB) as a PTA, as it is an inexpensive, carbon-based material commonly used as a pigment and additive in plastic products.^38,39^ Similarly, photothermal conversion can also be used for multiple different depolymerization and degradation mechanisms, such as poly­(ethylene terephthalate) (PET) solvolysis, poly­(l-lactide) (PLLA) ring-closing depolymerization, polystyrene (PS) and poly­(methyl methacrylate) (PMMA) radical unzipping, and polyethylene (PE) and polypropylene (PP) fragmentation.^39–46^ Unlike the previous polymers studied with photothermal conversion, which are easily reprocessed, PAs present a fundamentally different challenge due to the material’s high chemical stability. We envisioned that photothermal conversion would be a facile approach for the depolymerization of both aliphatic and aromatic PAs, leveraging localized heating to overcome the energetic challenges of PA depolymerization.

Here, we report an efficient platform for PA recycling using photothermal conversion to enable ring-closing depolymerization and acidic hydrolysis (Figure 1C). We applied photothermal depolymerization to PA6, PA66, and a wide range of other aliphatic and aromatic polyamides, the latter of which often degrades before melting. Our strategy rapidly produces near quantitative yields for aliphatic PAs and enables monomer recovery for even highly resistant aromatic and semiaromatic PAs. Notably, this system is applicable to postconsumer and mixed PA waste. Additionally, we leverage already existing black pigments in these materials as PTAs, which ultimately demonstrates this method as a facile and sustainable avenue for a circular plastic economy.

## Results and Discussion

We began our investigation by studying the photothermal depolymerization of PA6. Inspired by prior alkaline pyrolysis literature, we photothermally depolymerized PA6, achieving a 74% yield of CPL in just 10 min (Figure 2A, entry 1).^13–16^ These experiments were performed by simply placing a vial with a mixture of PA6 powder, CB, and potassium hydroxide (KOH), under a static vacuum, and irradiating the bottom of the vial with white light (Pages S8–S11).

**Figure 2:**
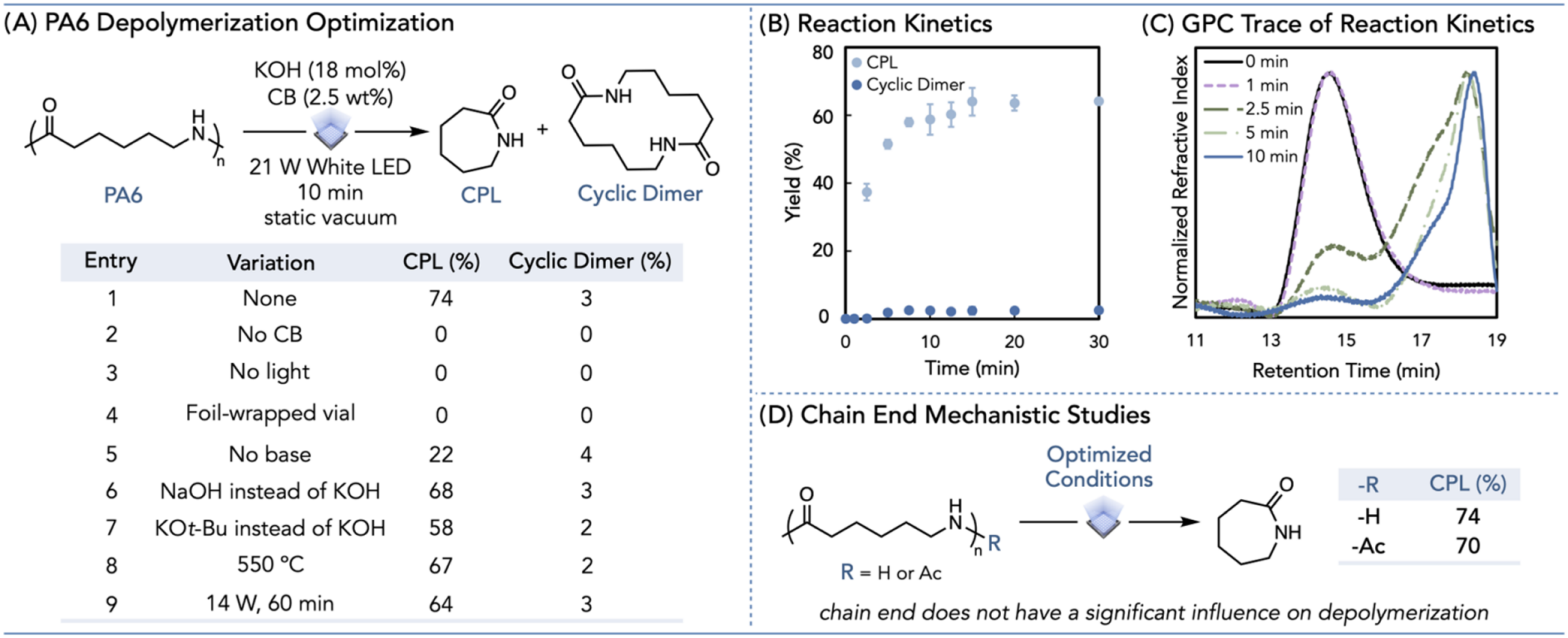
Photothermal ring-closing depolymerization of PA6. Detailed information on the photothermal reaction setup can be found in the Supporting Information (Pages S4–S6). (A) Reaction optimization table. The measured 21 W and 14 W light intensities were calculated to be 3.95 and 2.64 W/cm^2^, respectively. (B) Scatter plot of 14 W PA6 photothermal depolymerization products over time. Error bars represent the standard deviation across three trials. (C) Normalized gel-permeation chromatography (GPC) traces of PA6 after photothermal depolymerization using 14 W irradiation. (D) Chain-end mechanistic studies through AcPA6 depolymerization.

Without CB, we observed no monomer, demonstrating the necessity of a PTA for this system (Figure 2A, entry 2). Reactions run without light did not produce any CPL (Figure 2A, entry 3, Page S13). Similarly, we wrapped the reaction vial in foil and still observed no CPL yield, revealing any residual heat from the usage of the light-emitting diode (LED) light does not enable depolymerization (Figure 2A, entry 4, Page S13). The addition of a base was also shown to be beneficial for reactivity, as reactions without a base afforded only a 22% yield of CPL (Figure 2A, entry 5). Static vacuum was found to improve reaction yields over reactions run under nitrogen or air, likely owing to improved sublimation of CPL away from the photothermal agent and shifting the reaction equilibrium forward (Page S13).

Varying the base provided insight into the reactivity. KOH afforded improved yield compared to sodium and lithium hydroxide (Figure 2A, entry 6, Table S5) due to the weaker ion pairing of the potassium cation and increased relative availability of the basic anion for the reaction to proceed.^47^ Following the same trend, potassium *tert*-butoxide (KO*t-*Bu) also outperformed its sodium and lithium counterparts (Figure 2A, entry 7, Table S5). Organic bases, such as 1,5,7-triazabicyclo[4.4.0]­dec-5-ene (TBD) and 1,8-diazabicycloundec-7-ene (DBU), did not display the same performance as the inorganic bases tested (Table S5). These observed reaction trends are likely attributed to the thermal stability of the bases. Under our neat, vacuum, and photothermal conditions, TBD and DBU are susceptible to volatilization and sublimation, leading to poor colocalization of the base and polymer chain near the surface of the PTA, hindering depolymerization.^48,49^

Reactions run thermally at 550 °C for 10 min afforded 67% monomer, a comparable yield to our optimized conditions, demonstrating the rapid heat generation and high temperatures achieved using photothermal conversion (Figure 2A, entry 8, Pages S16 and S17). Finally, when a lower relative light intensity light is used, the CPL yield is slightly diminished with a longer reaction time (Figure 2A, entry 9, Page S20). This decreased yield is expected, as lower light intensity results in a lower maximum temperature near the PTA surface, consistent with other photothermally promoted reactivity.^34,37^

Next, we sought to probe the mechanism of the PA6 ring-closing depolymerization. From our previous optimization results, the addition of a base was found to improve reactivity. From this, we hypothesized that chain scission, from nucleophilic attack of the amide carbonyl, or chain unzipping, from midchain or chain-end deprotonation, were two plausible pathways for depolymerization. Thus, we turned to kinetics and molecular weight studies for further examination. Since our optimized reaction conditions using 21 W white light irradiation afforded over 40% CPL yield in just 1 min (Table S11 and Figure S20), we opted to use a lower-intensity 14 W white LED to monitor early reaction kinetics (Figure 2B, Page S20). Under these conditions, the CPL yield increased at a slightly slower but still logarithmic rate in the first 10 min (Figure 2B). Using gel permeation chromatography (GPC) analysis, we observed the residual polymer after 14 W white light irradiation exhibited a bimodal molecular weight distribution at the 2.5 and 5 min time points (Figure 2C, Pages S20–S24). This indicates that some polymer chains have rapidly decreased in molecular weight, whereas the remaining chains retain their initial molecular weight, seemingly unreacted.^39,50^ This distribution is characteristic of a chain-unzipping mechanism rather than chain scission, as the latter would result in a unimodal shift toward longer retention times and lower molecular weights.

While our molecular weight studies rule out chain scission as the primary depolymerization pathway, chain unzipping could occur via either chain-end or midchain deprotonation followed by cyclization of the monomer. As one of the chain-ends in our PA6 starting material was a free amine (Figure S8), we sought to synthesize PA6 with no amine-chain-ends that would be deprotonated when subjected to KOH in our depolymerization conditions (Figure 2D). PA6 with an acetyl chain end (AcPA6) was synthesized through the NaH-catalyzed anionic ring-opening polymerization using *N*-acetylcaprolactam (AcCPL) as an initiator (Pages S28–S31).^51,52^ The acetylated amide chain end of AcPA6 is chemically indistinct from midchain amides and is therefore as likely to be deprotonated as the midchain amides. Thus, if chain-end amine deprotonation is the main depolymerization pathway, then reactivity is expected to be inhibited or significantly slowed. To distinguish between these two pathways, AcPA6 was subjected to our optimized depolymerization conditions, in which we found that AcPA6 and PA6 afforded comparable yields of CPL and the cyclic dimer (Figure 2D and Page S32).

Additionally, the early depolymerization kinetics of AcPA6 revealed that CPL and the cyclic dimer were produced at very similar rates to those of PA6 with the amine-chain-end (Pages S33–S38). Furthermore, after 2.5 and 5 min of 14 W white light irradiation, we observed intact acetylated chain ends of AcPA6 by matrix-assisted laser desorption/ionization - time-of-flight mass spectrometry (MALDI-TOF MS) analysis, indicating that the AcPA6 acetylated chain end is unlikely to degrade during photothermal depolymerization (Figures S31–S34). These findings suggest that midchain amide deprotonation followed by chain unzipping is likely the dominant depolymerization mechanism for PA6 under our conditions (Figure S35). Similarly, we observe similar dimer yields for AcPA6 and PA6, indicating that midchain unzipping is likely to be the main pathway for the cyclic dimer formation (Figure S36).

Next, to illustrate the sustainability potential of our system, we explored the use of focused sunlight irradiation as the sole light source for PA6 depolymerization (Figure 3A, Pages S40–S41). Gratifyingly, a 60% CPL yield was observed within 3 min under sunlight irradiation, comparable to yields achieved with our laboratory setup (Figure 3A, Table S11 entry 4). With efficient monomer generation established, we sought to address monomer isolation and reuse, as impurities in CPL often negatively impact the polymerization of PA6.^9,53^ Therefore, we employed the use of a fractional dynamic vacuum sublimation setup, where CPL is able to sublimate on the sides of the vacuum adapter away from the vial, leaving the remaining polymer, PTA, and base at the bottom of the vial near the light source (Figure S38). With this setup, monomer separation is facile, allowing us to isolate CPL in situ, in over 98% purity and in a yield comparable to our optimized conditions (Figure 3B, Page S45). The recovered CPL was then repolymerized to PA6 using previously reported methods, showing that photothermal conversion is a feasible route for a cyclic plastic economy (Figure 3B, Pages S45–S48).^51,52^

**Figure 3:**
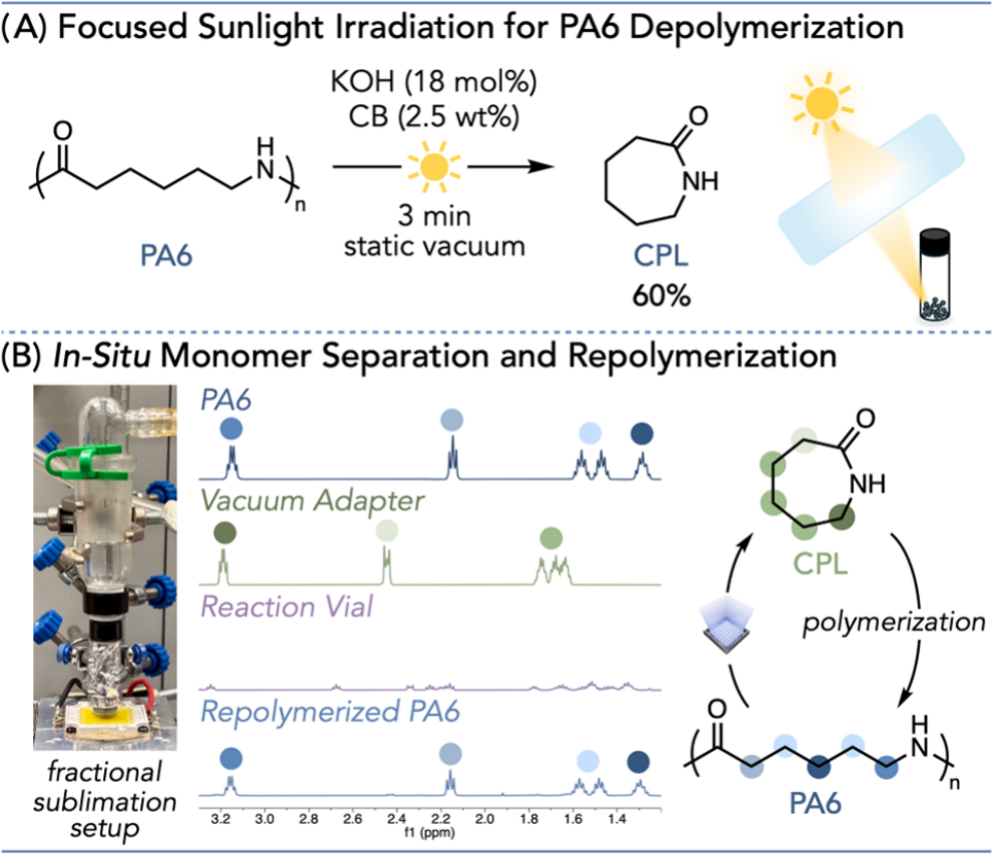
(A) PA6 photothermal depolymerization using focused sunlight. (B) *In situ* monomer separation under dynamic vacuum and repolymerization. ^1^H NMR spectra of the vacuum adapter and the reaction vial (CDCl_3_) are from dynamic vacuum experiments. ^1^H NMR spectrum of PA6 and repolymerized PA6 (3:1 TFE/CDCl_3_) is from the repolymerization of collected CPL from dynamic vacuum experiments.

Following our success with PA6, we utilized photothermal conversion for PA66 depolymerization, the second-most produced PA after PA6.^54^ Unlike PA6, PA66 is produced by polycondensation of two monomers, adipic acid (AA) and hexamethylenediamine (HMDA), and therefore requires different depolymerization conditions to obtain these synthetically useful monomers.^7^ By irradiating a mixture of PA66 powder, CB, and aqueous acid under air, we depolymerized PA66 nearly quantitatively using photothermally mediated acidic hydrolysis, affording 97 and 96% of HMDA and AA, respectively (Figure 4A, entry 1, Pages S49–S52). Control reactions without light or CB did not yield either monomer, displaying that this process is photothermally driven (Figure 4A, entries 2 and 3, Page S53). Furthermore, this method is dependent on aqueous acid, as reactions run without it did not produce either monomer (Figure 4A, entry 4, Page S53).

**Figure 4:**
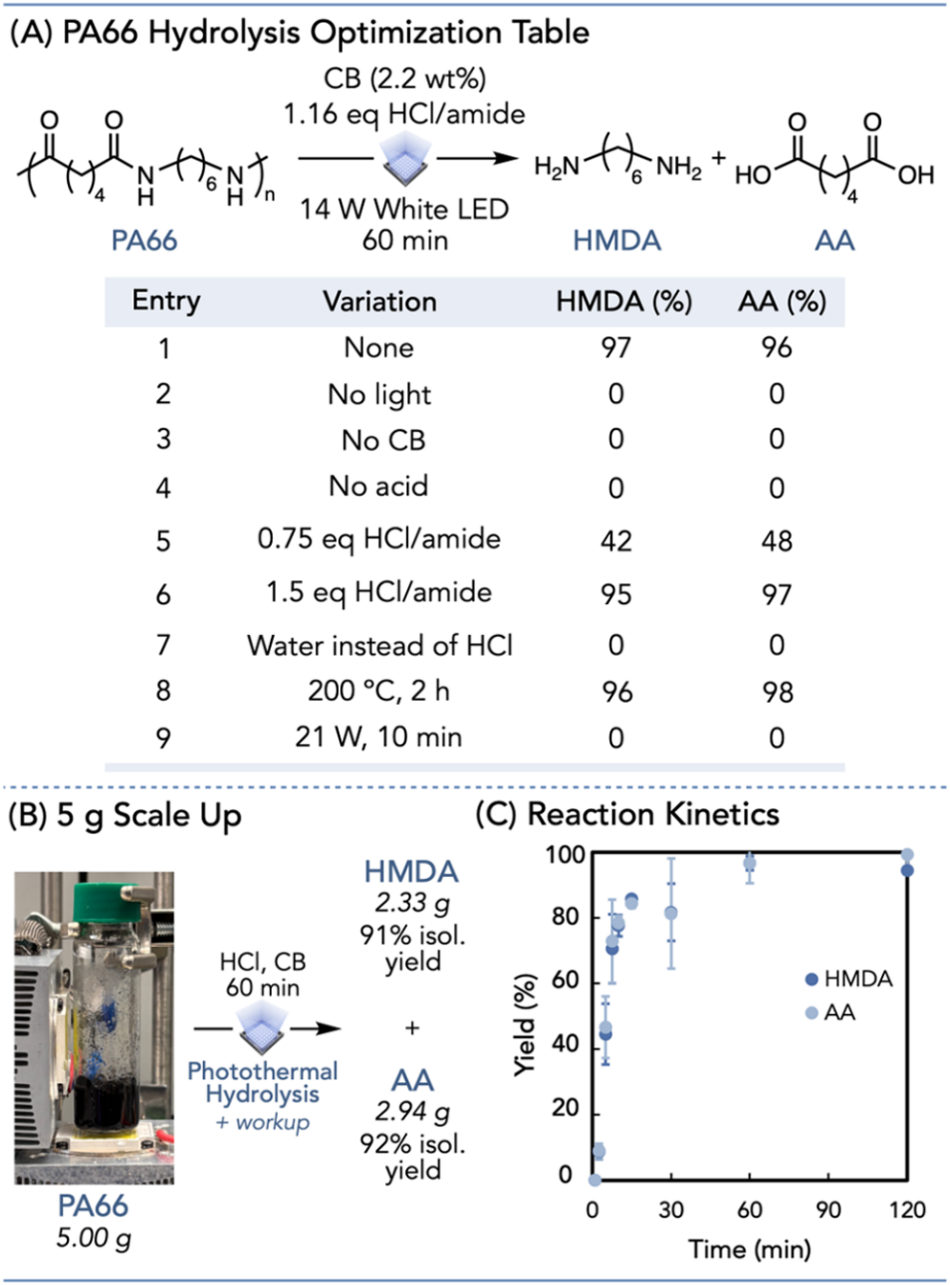
(A) Photothermal PA66 acidic hydrolysis optimization table. (B) Large-scale PA66 photothermal acidic hydrolysis. (C) Scatter plot of PA66 photothermal hydrolysis products over time. Error bars represent the standard deviation across two trials.

We found that a stoichiometric ratio of acid to amide was necessary to hydrolyze PA66 to appreciable yield, with lower equivalents affording less than 50% of each monomer (Figure 4A, entries 5 and 6, Pages S55 and S56). Similarly, depolymerization did not occur when only water was used (Figure 4A, entry 7, Page S53). When subjected to traditional bulk heating at 200 °C for 2 h, a temperature commonly used for thermal PA66 hydrolysis, HMDA and AA were produced in 96 and 98% yields, respectively, demonstrating the rapid heat generation from photothermal conversion allowing for faster reactivity (Figure 4A, entry 8, Pages S57 and S58).^10^ As opposed to the ring-closing depolymerization, a higher intensity light proved to be detrimental for this hydrolysis process. It did not yield any product, likely owing to higher temperatures near the PTA surface during photothermal conversion resulting in HCl volatilization to the vial headspace and polymer degradation (Figure 4A, entry 9, Pages S59–S61).^55^ Finally, we easily scaled this reaction to 5 g, achieving an isolated yield of 90% for each monomer (Figure 4B, Pages S64–S67). By tracking monomer formation over the course of the reaction, we found that we reliably achieve near-quantitative yields of HMDA and AA at 60 min, with a gradual increase in monomer yield over the first hour and no product decomposition at longer reaction times (Figure 4C, Page S63). These observations suggest a chain-scission mechanism, consistent with proposed mechanisms of traditional thermal hydrolysis procedures.^18,56–58^

To broaden our polymer scope, we also applied our photothermal hydrolysis conditions to aliphatic homo- and copolyamides (Figure 5A,B). Polyamide 11 (PA11) and polyamide 12 (PA12) are used in electrical components, textiles, additive manufacturing, and other applications.^59^ The photothermal hydrolysis method was used for these polymers, as polycondensation of 11-aminoundecanoic acid (11-AUDA) and 12-aminododecanoic acid (12-ADDA) is a common polymerization route for PA11 and PA12, respectively. PA11 and PA12 are less polar than PA66, making them more resistant to hydrolysis.^7,23,60^ Therefore, we used a higher acid to amide ratio and found that both PA11 and PA12 were readily hydrolyzed into their linear open-chain monomers in over 90% yield (Figure 5A, Pages S68 and S69). Similar to PA66, the aliphatic copolymer polyamide 6,10 (PA610) is also well tolerated by this system, affording over 95% sebacic acid and 94% HMDA (Figure 5B, Pages S71 and S72).

**Figure 5:**
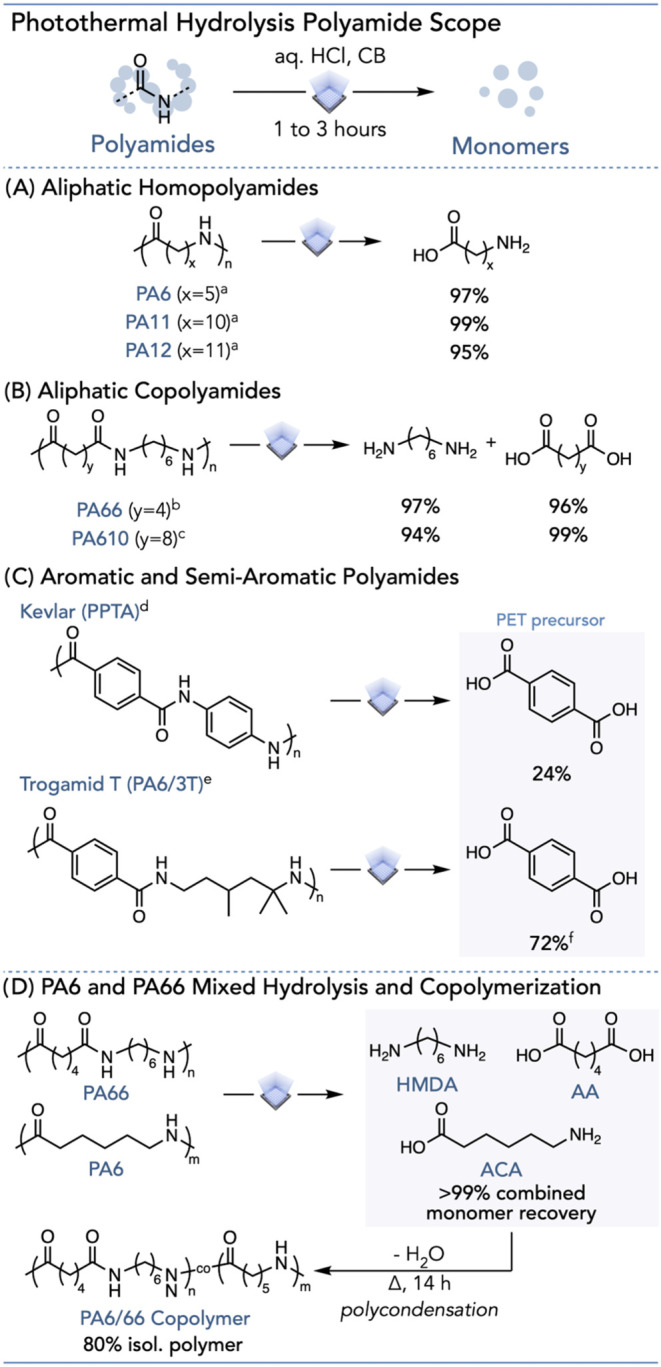
Acidic hydrolysis PA scope. All reactions use 2.2 wt % CB and 14 W White LED light irradiation. (A) Aliphatic homopolyamides. (B) Aliphatic copolyamides. (C) Aromatic and semiaromatic polyamides. (D) Photothermal hydrolysis of mixed PA6 and PA66 and polycondensation of recovered monomers. The mixed hydrolysis used 2.5 mol equiv of HCl/amide and a 1-h reaction time. ^a^ Reaction time of 1 h and 2.5 mol equiv of HCl/amide were used. ^b^ Reaction time of 1 h and 1.16 mol equiv of HCl/amide were used. ^c^ Reaction time of 1 h and 1.5 mol equiv of HCl/amide were used. ^d^ Reaction time of 3 h and 1.25 mol equiv of HCl/amide were used. ^e^ Reaction time of 1 h and 1.25 mol equiv of HCl/amide were used. ^f^ Isolated yield.

To demonstrate the applicability of photothermal depolymerization on very chemically and thermally resistant polymers, we used our approach on the aromatic polyamide poly­(*p*-phenylene terephthalamide) (PPTA), also known as Kevlar. PPTA is commonly used in bulletproof vests, bicycle tires, and other high-impact applications (Figure 5C, Pages S73 and S74).^61^ PPTA displays superior mechanical properties compared to many other thermoplastics due to its strong intermolecular forces, including π–π stacking and hydrogen bonding, which allow for a highly crystalline microstructure.^62^ Consequently, PPTA degrades before melting with degradation temperatures above 400 °C, behaving similarly to a covalently cross-linked thermoset.^63^ We envisioned that photothermal conversion could facilitate PPTA hydrolysis, a reaction that typically occurs at 250 °C.^29,64^ Despite the diamine monomer degrading under light irradiation, we observed a 24% yield of terephthalic acid (TPA), a precursor to PET.^65^ On the other hand, the semiaromatic polyamide poly­(trimethyl hexamethylene terephthalamide) (PA6/3T), also known as Trogamid T, has a more amorphous character compared to other PAs, due to the structural irregularity of its diamine.^66,67^ Nevertheless, PA6/3T also readily depolymerizes under these conditions, and TPA was isolated in 72% yield by simple base extraction (Figure 5C, Pages S75 and S76).

To address mixed plastic waste streams, we envisioned using our photothermal acidic hydrolysis method to depolymerize mixed PAs (Figure 5D). We achieved near-complete hydrolysis of a 1:1 mixture of PA6 and PA66, resulting in the quantitative recovery of HMDA, AA, and 6-ACA (Figure 5D, Pages S77 and S78). Moreover, we repolymerized the monomer mixture from the PA6/PA66 hydrolysis back into a PA6/66 copolymer, commonly used for additive manufacturing, thereby demonstrating the potential for a circular plastic economy (Pages S79–S81).^68^ Additionally, we can extend this mixed PA hydrolysis system to other PA combinations, including PA11/PA12 and PA66/PA610, affording over 95% of the monomers (Pages S82 and S83).

Lastly, we demonstrated that our photothermal conditions can depolymerize commercial samples. Commercial PA products often contain additives such as plasticizers, pigments, and fillers, which can be incompatible with certain recycling methods, and black plastics are recycled to a lesser extent than their uncolored counterparts.^4^ Postconsumer PA6 samples, including PA6 tubing, carpeting, and thread, were subjected to our photothermal depolymerization conditions without any prior processing (Figure 6A). To our delight, all of the commercial samples tested were effectively depolymerized into CPL (Pages S87–S90). Since black pigments in many commercial products can act as PTAs, we performed the same depolymerization experiments without additional CB, achieving up to 74% CPL recovery (Table S32). However, white samples, such as the PA6 thread, did not depolymerize to an appreciable extent in the absence of CB. Using a mixture of commercial samples as our starting material, we found that a 3:1 ratio of white thread to black t-shirt yielded up to 42% monomer (Page S93). Furthermore, using focused sunlight as the light source, a black PA6 t-shirt was depolymerized to afford a 70% yield of CPL without added CB, demonstrating the simplicity and tolerance of our photothermal ring-closing depolymerization method (Pages S91 and S92).

**Figure 6:**
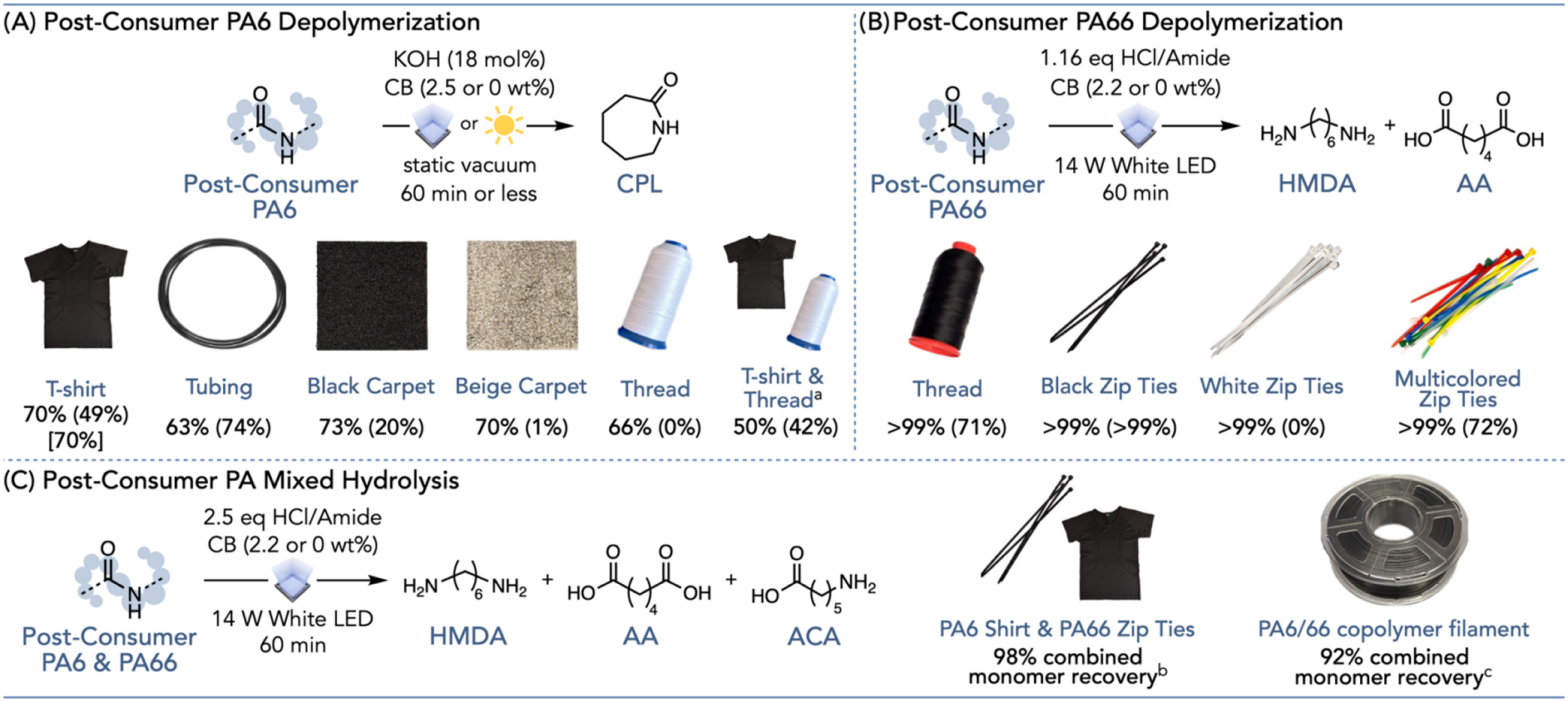
(A) Depolymerization of postconsumer PA6 samples. Yields are reported after correcting for plastic purity, where yields outside of parentheses are reaction yields with 2.5 wt % CB and yields in parentheses are reaction yields with 0 wt % CB. Yields in brackets use focused sunlight instead of LED light, with a 3 min reaction time and 2.5 wt % CB. Detailed reaction conditions and purity calculations are provided in the Supporting Information (Pages S84–S93). (B) Postconsumer PA66 photothermal depolymerization. Yields are reported after correcting for plastic purity and are averages of the individual HMDA and AA yields. Yields outside of parentheses are reaction yields with 2.2 wt % CB, and yields in parentheses are reaction yields with 0 wt % CB. Detailed reaction conditions and purity calculations are provided in the Supporting Information (Pages S94–S101). (C) Photothermal hydrolysis of PA6 T-shirt and PA66 zip ties and PA6/66 copolymer filament, where combined monomer recovery is reported. Detailed reaction conditions and purity calculations are provided in the Supporting Information (Pages S102–S108). ^a^ A 3:1 mixture of white PA6 thread and black PA6 T-shirt was used, and yields are reported after correcting for purity of the mixture. ^b^ 0 wt % of CB was used, and monomer recovery is scaled for PA6 and PA66 purity. ^c^ 2.2 wt % of CB was used, and monomer recovery is not scaled for PA purity.

PA66 commercial products were also well tolerated by photothermal hydrolysis conditions, yielding nearly quantitative amounts of HMDA and AA (Figure 6B, Pages S98–S101). Notably, multicolored zip ties were hydrolyzed with yields comparable to those of other commercial samples, demonstrating tolerance to dyes and pigments that may be present. Similar to our previous results with PA6, black commercial PA66 samples were hydrolyzed without an additional CB (Table S37). We extended our mixed PA hydrolysis method to PA6 and PA66 postconsumer waste, with a mixture of a black PA6 T-shirt and black PA66 zip ties yielding near-quantitative monomer recovery by using the already existing black pigments in the commercial materials (Figure 6C, Pages S102 and S103). Moreover, the PA6/66 copolymer filament was depolymerized into its constituent monomers by this system and subsequently repolymerized to afford the PA6/66 copolymer, thus demonstrating that photothermal depolymerization can tolerate more complex polymer architectures (Figure 6C, Pages S104–S108).

## Conclusions

Here, we demonstrate that photothermal conversion enables a rapid and generalizable strategy for PA depolymerization through ring-closing depolymerization and acidic hydrolysis. Ring-closing depolymerization of PA6 affords CPL within minutes and can be performed by focused sunlight irradiation. Photothermal hydrolysis delivers near quantitative yields of monomer for aliphatic polyamides, including PA6 and PA66, and can be extended to generally resistant aromatic and semiaromatic polyamides, such as PPTA and PA6/3T. Additionally, both depolymerization mechanisms tolerate postconsumer and mixed PA waste, further illustrating the method’s applicability for use in a circular plastic economy.

## Supplementary Material



## Notes

Experimental methods, spectral data, and photographs of this work (PDF)
