# Supplementary material for: Universal Approach for the Depolymerization of Polyamides via Photothermal Conversion
Source: J Am Chem Soc. 2026 Apr 23;148(17):18256–64. doi: 10.1021/jacs.6c03063 (PMC13154200; doi:10.1021/jacs.6c03063)
Supplement: Supplementary file 1 [file ja6c03063_si_001.pdf]

Supporting Information

A Universal Approach for the Depolymerization of Polyamides via Photothermal Conversion

Deepika Shingwekar and Erin E. Stache*
Corresponding author: Erin E. Stache, estache@princeton.edu

Department of Chemistry, Princeton University, Princeton, New Jersey, 08544, United States

Pages S1 to S114
Figures S1 to S108
Tables S1 to S39

Table of Contents

Table of Contents **2**

Materials and Methods **4**

General Reagent Information 4

Photothermal Reaction Setup 4

Characterization Methods 6

Photothermal Hydrolysis Safety Precaution 7

PA6 Photothermal Ring-Closing Depolymerization Optimization **8**

General Procedure for PA6 Photothermal Ring-Closing Depolymerization 8

Varied Carbon Black Loading for PA6 Photothermal Ring-Closing Depolymerization 12

Control Experiments for PA6 Photothermal Ring-Closing Depolymerization 13

Varied KOH Loading for PA6 Photothermal Ring-Closing Depolymerization 14

Base Screen for PA6 Photothermal Ring-Closing Depolymerization 15

Thermal Controls for PA6 Photothermal Ring-Closing Depolymerization 16

Light Intensity Screen for PA6 Photothermal Ring-Closing Depolymerization 18

Large scale PA6 Photothermal Ring-Closing Depolymerization 19

PA6 Mechanistic Studies **20**

14 W Light Intensity Kinetics for PA6 Photothermal Ring-Closing Depolymerization 20

21 W Light Intensity Kinetics for PA6 Photothermal Ring-Closing Depolymerization 25

Anionic Ring-Opening Polymerization for AcPA6 synthesis 28

AcPA6 Photothermal Ring-Closing Depolymerization using Optimized Conditions 32

AcPA6 Photothermal Ring-Closing Depolymerization using 14 W Light intensity 33

Proposed Mechanism for CPL and Cyclic Dimer formation from PA6 39

PA6 Photothermal Depolymerization Using Focused Sunlight as a Light Source **40**

PA6 Photothermal Depolymerization under Dynamic Vacuum **42**

Repolymerization of CPL after PA6 Photothermal Depolymerization 45

PA66 Photothermal Acidic Hydrolysis Optimization **49**

General PA66 Photothermal Acidic Hydrolysis Conditions 49

Control Experiments for PA66 Photothermal Acidic Hydrolysis 53

Varied Carbon Black Loading for PA66 Photothermal Acidic Hydrolysis 54

Varied Acid Loading (constant volume) for PA66 Photothermal Acidic Hydrolysis 55

Varied Acid Loading (constant HCl/Amide molar ratio) for PA66 Photothermal Acidic Hydrolysis... 56

Thermal Controls for PA66 Acidic Hydrolysis 57

Light Intensity Screen for PA66 Photothermal Acidic Hydrolysis 59

Acid Screen for PA66 Photothermal Acidic Hydrolysis 62
PA66 Photothermal Acidic Hydrolysis Kinetics 63
Large Scale PA66 Photothermal Acidic Hydrolysis 64
PA Photothermal Acidic Hydrolysis Scope 68
PA11 Photothermal Acidic Hydrolysis 68
PA12 Photothermal Acidic Hydrolysis 69
PA6 Photothermal Acidic Hydrolysis 70
PA610 Photothermal Acidic Hydrolysis 71
PPTA Photothermal Acidic Hydrolysis 73
PA63T Photothermal Acidic Hydrolysis 75
Mixed PA Photothermal Acidic Hydrolysis 77
Mixed PA6 and PA66 Photothermal Acidic Hydrolysis 77
Polycondensation of HMDA, AA, and ACA from Mixed PA6 and PA66 Photothermal Acidic Hydrolysis 79
Mixed PA11 and PA12 Photothermal Acidic Hydrolysis 82
Mixed PA66 and PA610 Photothermal Acidic Hydrolysis 83
Post-Consumer PA6 Photothermal Ring-Closing Depolymerization 84
Purity Determination for PA6 Post-Consumer Samples 84
Photothermal Ring-Closing Depolymerization of PA6 Post-Consumer Samples 87
Photothermal Ring-Closing Depolymerization of PA6 T-shirt Using Focused Sunlight Irradiation 91
Photothermal Ring-Closing Depolymerization of Mixed Post-Consumer PA6 93
Post-Consumer PA66 Photothermal Acidic Hydrolysis 94
Purity Determination for PA66 Post-Consumer Samples 94
Post-Consumer PA66 Photothermal Acidic Hydrolysis 98
Mixed Post-Consumer PA Photothermal Acidic Hydrolysis 102
Post-Consumer Mixed PA6 and PA66 Photothermal Acidic Hydrolysis 102
PA6/66 Copolymer Filament Photothermal Acidic Hydrolysis 104
Polycondensation of HMDA, AA, and ACA from PA6/66 Copolymer Filament Photothermal Acidic Hydrolysis 106
Bulk Temperature Measurements of Photothermal Reactions 109
21 W Light Intensity Bulk Temperature Measurement Using a Digital Thermometer 109
14 W Light Intensity Bulk Temperature Measurement Using a Thermal Camera 111
Photothermal Ring-Closing Depolymerization for PA11 and PA12 112
References 114

Materials and Methods

General Reagent Information

Carbon black (CB) (amorphous, acetylene 100 % compressed, Alfa Aesar), hydrochloric acid (HCl) (GR ACS 37 %, Sigma Aldrich), 6-aminocaprioic acid (ACA) (95%, Combi-Blocks), adipic acid (AA) (99%, Sigma Aldrich), hexamethylene diamine (HMDA) (99%, Oakwood), sodium hydride (NaH) (60 % dispersion in mineral oil, Sigma Aldrich), Polyamide 6 (PA6) (500 mesh, Magerial), Polyamide 66 (100 mesh, Magerial), poly(trimethyl hexamethylene terephthalamide) (PA63T) (Scientific Polymer Products), and PA610 (Scientific Polymer Products) were used as received. Polyamide 11 (PA11) (Sigma Aldrich) and Polyamide 12 (PA12) (Sigma Aldrich) were pressed into 1-mm thick films at 195 °C and 185 ºC, respectively, and cut into 1-mm by 1-mm squares prior to use. Potassium hydroxide (KOH) (90%, Sigma Aldrich) was finely ground using a mortar and pestle before use. Caprolactam (CPL) (99%, Oakwood) was distilled and dried under vacuum prior to use in polymerization. 1-Acetylazepan-2-one (AcCPL) (98%, Oakwood) was distilled prior to use in polymerization. Poly(p-phenylene terephthalamide) (PPTA) (Fibre Glast Kevlar pulp filler binder) was purchased from Amazon.com. 1,3,5-Trimethoxybenzene (TMB) (99%, Sigma Aldrich), dimethyl sulfone ($DMSO_2$) (98%, Sigma Aldrich), maleic acid (99%, Sigma Aldrich), and 2-(4-Hydroxyphenylazo)benzoic acid (HABA) (95%, Combi-Blocks) were used without further purification. Deuterated chloroform ($CDCl_3$) (98%, Cambridge Isotope Laboratories Inc.), dimethyl sulfoxide-d_6 (DMSO-d_6) (99.9%, Cambridge Isotope Laboratories Inc.), deuterium oxide (D_2O) (99.9%, Cambridge Isotope Laboratories Inc.), 2,2,2-Trifluoroethanol (TFE) (99.9%, Oakwood), 1,1,1,3,3,3-Hexafluoroisopropanol (HFIP) (99.5%, Oakwood) were used as received. Post-consumer polymers were donated by Stache lab members or purchased from Amazon.com. All post-consumer polymers were cut into 1-mm by 1-mm pieces prior to use.

Photothermal Reaction Setup

Low-intensity (15 W or lower) laboratory photochemical reactions were irradiated using a 100 W 6000K white Chanzon LED chip equipped with a TX Aluminum Heatsink Cooling Fan and powered with a HLG-120H-36A LED power supply purchased from Mean Well. High-intensity (16 W or higher) laboratory photochemical reactions were irradiated using a 100 W 6000K white Chanzon LED chip cooled with a copper, 40-mm by 40-mm water block and powered by a HLG-120H-36A LED power supply purchased from Mean Well. Ice water was flowed through the copper water block using a fish pump to cool the LED chip, maintaining an average LED chip temperature of 25 ºC. Each piece of the setup was purchased individually from either Amazon.com or Mouser Electronics and wired manually. Light intensity was adjusted using the built-in current potentiometer on the LED power supply. Precaution was taken to cover all photochemical reactions with a cardboard box during irradiation. For reactions that used focused sunlight as a light source, plastic Fresnel lenses (21.1 cm x 29.8 cm and 24.1 cm x 16.5 cm) purchased from Amazon.com were used to focus sunlight, with focal area of 1.77 cm^2.

For laboratory photochemical reactions, light intensity was measured using the PMD 100D compact power and energy meter equipped with the thermal power sensor head (2.6 cm diameter) purchased from Thor Labs. The light intensities throughout the main text and SI are referred to in units of Watts (W). The light intensity per unit area (in units of W/cm^2) can be calculated by

dividing the wattage readout by the area of the ThorLabs compact power and energy meter (5.31 cm^2), and listed below for reference:

Light Intensity (W)	Light Intensity per Area (W/cm^2)	Light Intensity (W)	Light Intensity per Area (W/cm^2)
10	1.88	15	2.82
11	2.07	16	3.01
12	2.26	17	3.20
13	2.45	18	3.39
14	2.64	21	3.95

Light Intensity per area (W/cm^2) = [Light intensity] / [Power Meter Area]
Power Meter Area = 5.31 cm^2

For reactions that used focused sunlight as a light source, the light intensity per area (in W/cm^2) was estimated by dividing the Fresnel lens area by the focal point area and multiplying by the unfocused sun intensity data (solar radiation, obtained from https://www.njweather.org/charts, New Jersey, USA). The light intensity per area was determined to range from 17.7 to 28.1 W/cm^2.

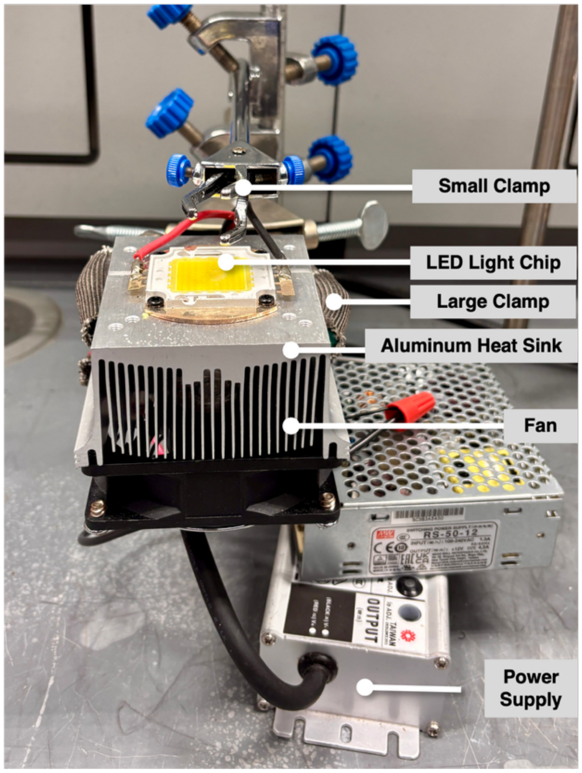

Figure S1. Low-intensity light setup, used for all photothermal reactions with intensities of 15 W and lower.

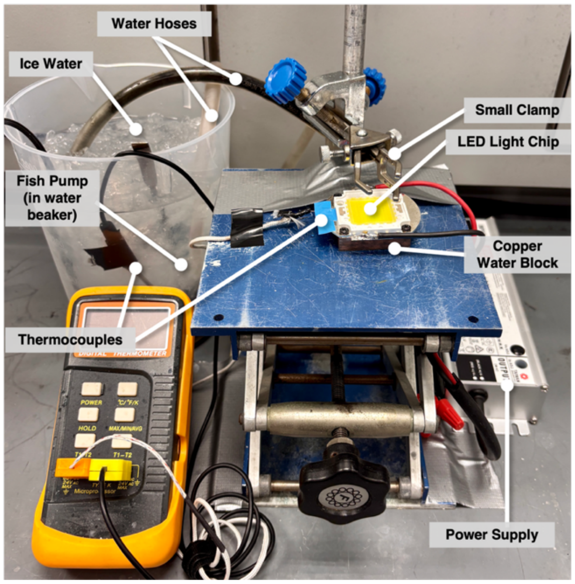

Figure S2. High-intensity light setup, used for all photothermal reactions with intensities of 16 W and higher.

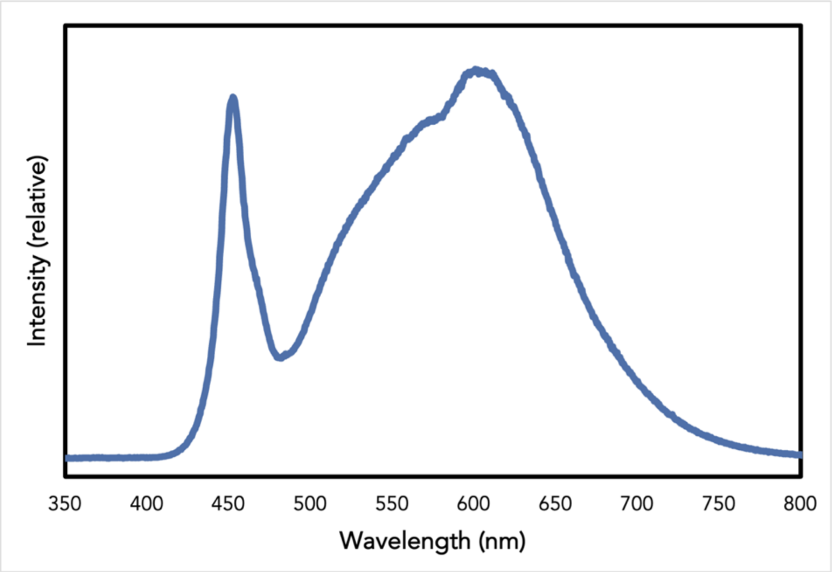

Figure S3. Emission spectra of white LED light.

Characterization Methods

Gel-Permeation Chromatography (GPC) –Number-average molecular weights (M_n) and dispersities ($Đ$, where $Đ = M_w/M_n$) of polymer samples were analyzed using a Tosoh EcoSEC HLC 8240 system using two TSKgel SuperAMW-H columns in series, with a Tosoh LenS3 Multi-Angle Light Scattering detector and a built-in Refractive Index detector. The flow rate of 0.350 mL/min. 1,1,1,3,3,3-Hexafluoroisopropanol with 0.05 M potassium trifluoroacetate was used as the eluent. The system was calibrated using PMMA standards (Agilent, ReadyCal).

Fourier-Transform Infrared Spectroscopy (FTIR) – A Nicolet 6700 FTIR instrument equipped with an attenuated total reflectance (ATR) set up was used to collect data. Spectra were taken with 4 cm^{-1} resolution with 4 background scans and sample scans.

Nuclear Magnetic Resonance (NMR) – Bruker 500 MHz NMR instruments were used to obtain ^{1}H and ^{13}C NMR spectra at room temperature using DMSO-d_6, D_2O, $CDCl_3$, and a 3:1 mixture of TFE/$CDCl_3$ as the NMR solvent. External standards, 1,3,5-trimethoxybenzene, maleic acid, and dimethyl sulfone, were used depending on the NMR solvent. All PA66 hydrolysis NMR data was collected on an NMR instrument with a room temperature probe to avoid peak broadening and uneven baselines caused by high ionic concentrations within the sample.

Matrix-Assisted Laser Desorption/Ionization - Time-of-Flight Mass Spectrometry (MALDI-TOF MS) – Spectral data were collected on a Bruker Auto flex maX MALDI-TOF instrument (positive reflectron mode, signal suppression under 4000 m/z). To prepare samples, a solution of polymer (5 mg/mL) in HFIP was made and let sit overnight to dissolve. Once fully dissolved, the polymer solution (20 μL) and a saturated solution of HABA in HFIP (20 μL) were mixed well, then applied in a 1 μL aliquot onto a stainless-steel target.

qNMR Calculation Method for Depolymerization – The following equation was used for calculating the yield (%) of small molecules after polyamide depolymerization via ^{1}H NMR:

$$Yield\ (\%) = \frac{a \times b}{c} 100\%$$

a = the moles of internal standard added
b = the ratio of the moles of small molecule to internal standard
c = the moles of the polyamide repeating unit in the initial polyamide amount used*

*The moles of repeating unit for each polyamide are found through the molar mass of the repeating unit. For homopolyamides (PA6, PA11, and PA12), the repeating unit molar mass the same as their cyclic monomer molar masses. For copolyamides (PA66, PA610, PPTA, and PA63T), the repeating unit molar mass is the mass of two water molecules (32 g/mol) subtracted from the sum of the diacid and diamine molar masses. Therefore, depolymerization yields for copolyamides are scaled for the amount of each monomer, rather than a mol %.

Photothermal Hydrolysis Safety Precaution

Photothermal acidic hydrolysis reactions can generate hydrogen chloride (HCl) gas, particularly under photothermal conditions or if excess aqueous HCl is used, which may lead to pressure buildup and potential vial rupture. Therefore, a user must wear the personal protection equipment (lab coat, protection glasses, and gloves) and perform all experiments in a well-ventilated area. Reaction vials should be appropriately sized to handle any pressure buildup and care should be taken to avoid reactions with excess aqueous HCl.

PA6 Photothermal Ring-Closing Depolymerization Optimization

General Procedure for PA6 Photothermal Ring-Closing Depolymerization

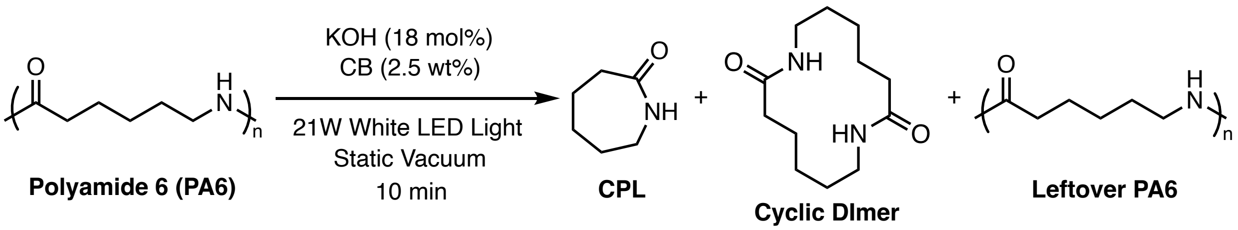

In a 1-dram glass vial, PA6 (77.4 mg, 0.684 mmol), ground KOH (6.9 mg, 0.123 mmol, 0.18 mol equivalents), and CB (2.2 mg, 2.5 wt% of total reaction volume) were added, sealed with a cap fitted with a Teflon septum, and vortexed for 1 minute to mix until thoroughly combined. The vial was evacuated and backfilled with nitrogen three times and left under static vacuum after a fourth vacuum pull. The vial was subsequently placed 0.2 mm above a 6000K white LED light (21 W) and irradiated for 10 minutes. After the reaction, the vial was cooled in a dry ice-acetone bath for 1 minute and defrosted to room temperature. $CDCl_3$ (1 mL) and a stock solution of 1,3,5-trimethoxybenzene stock solution (0.2 mL, 0.12M in $CDCl_3$) were added to the reaction vial. Aliquots were taken for ^{1}H NMR analysis (0.1 mL of the dissolved reaction mixture was diluted with 0.35 mL $CDCl_3$).

To determine the amount of leftover PA6 after photothermal ring-closing depolymerization, a modified workup procedure was performed. After the reaction, dimethyl sulfone ($DMSO_2$) (5 to 9 mg, 0.053 to 0.096 mmol) and a 3:1 mixture of TFE/$CDCl_3$ (3.5 mL) were added to the reaction vial. The vial was vortexed to dissolve all residual polymer, and aliquots were taken for ^{1}H NMR analysis.

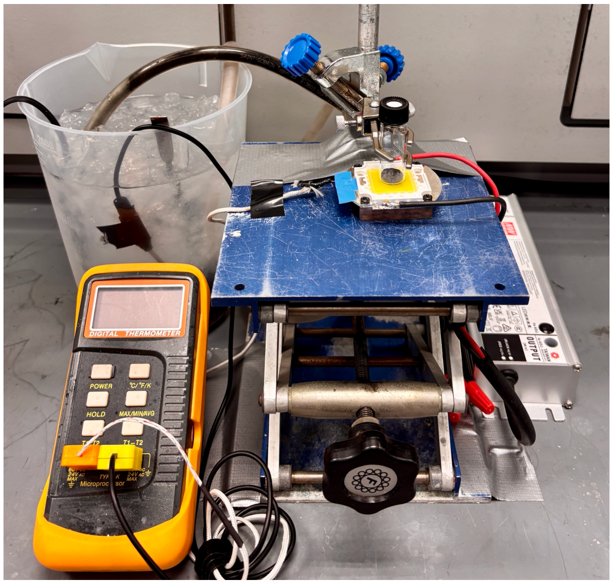

Figure S4. Reaction setup of PA6 ring-closing photothermal depolymerization.

Table S1. Results of PA6 ring-closing photothermal depolymerization.

Entry	CPL Yield$_{NMR}$(%)	Dimer Yield$_{NMR}$ (%)	Leftover PA6 (%)	NMR mass recovery (%)
1	74.1 (± 2.5)	3.0 (± 1.2)	11.5 (± 3.8)	91.6 (± 3.2)

Each entry is an average of 3 trials. Error represented is the standard deviation between trials.
NMR mass recovery includes leftover PA6 after depolymerization.

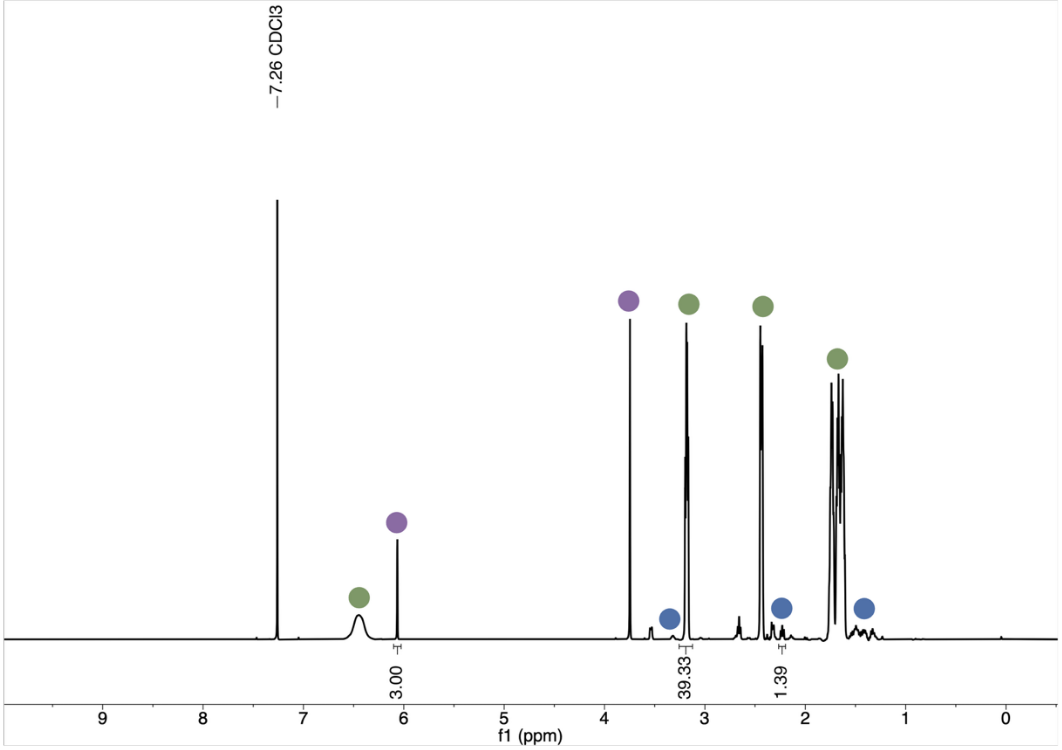

Figure S5. ^{1}H NMR of PA6 after photothermal ring-closing depolymerization in $CDCl_3$. CPL (green circles), cyclic dimer (blue circles) and TMB (purple circles) signals are labeled. The cyclic dimer signals are consistent with prior literature data.[1]

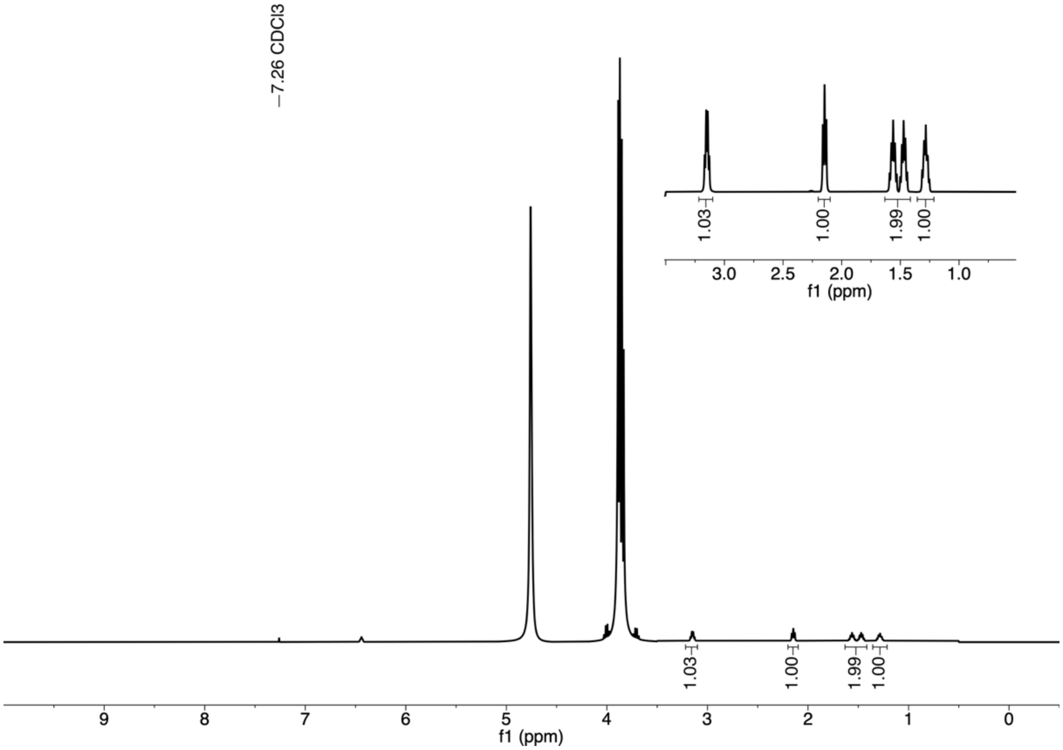

Figure S6. ^{1}H NMR of PA6 in 3:1 TFE/$CDCl_3$. The spectrum was baseline corrected between the 3.5 to 0.5 ppm region to ensure that protonated TFE signals appearing at 3.8 ppm and 4.9 ppm would not interfere with integrations.

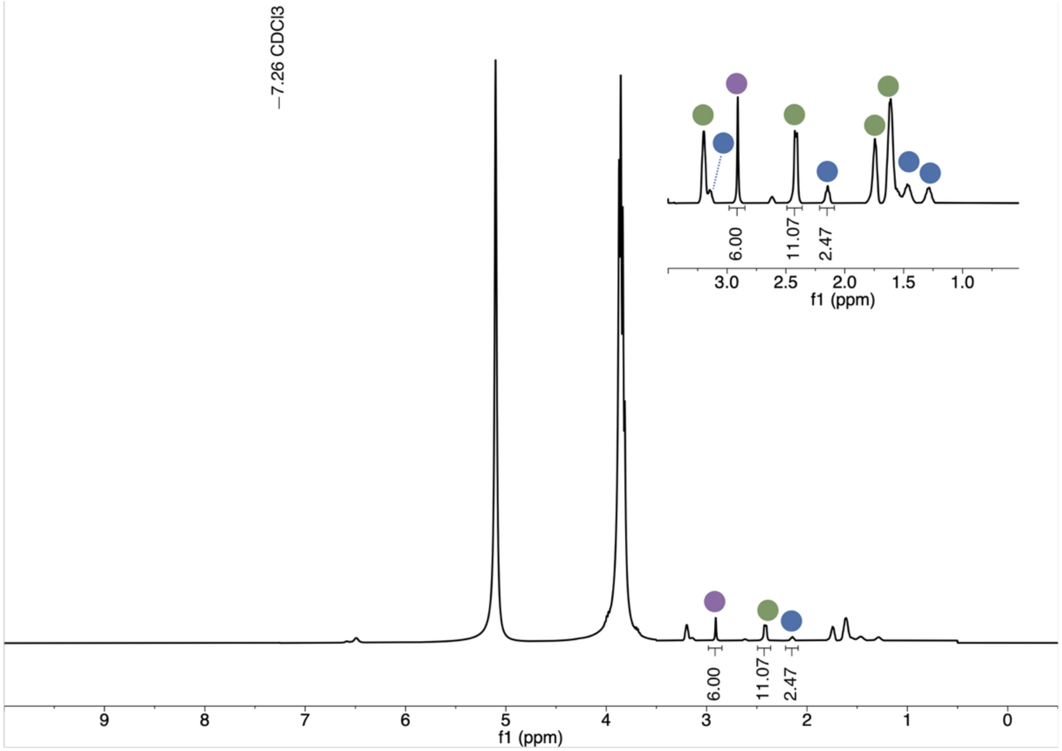

Figure S7. ^{1}H NMR of PA6 after photothermal ring-closing depolymerization in 3:1 TFE/$CDCl_3$. The spectrum was baseline corrected between the 3.5 to 0.5 ppm region to ensure that

protonated TFE signals appearing at 3.8 ppm and 5.1 ppm would not interfere with qNMR calculations. CPL (green circles), leftover PA6 (blue circles) and $DMSO_2$ (purple circles) signals are labeled.

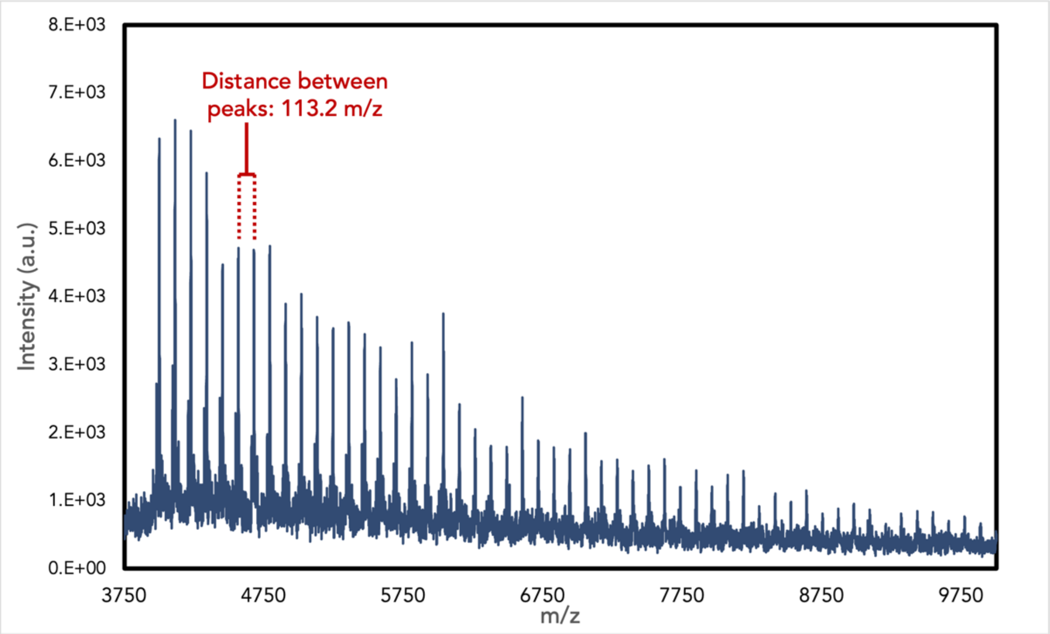

Figure S8. MALDI-TOF mass spectrum of purchased PA6 between 3750 and 10000 m/z. The spacing between peaks correlates well to the PA6 repeating unit mass (113.2 g/mol).

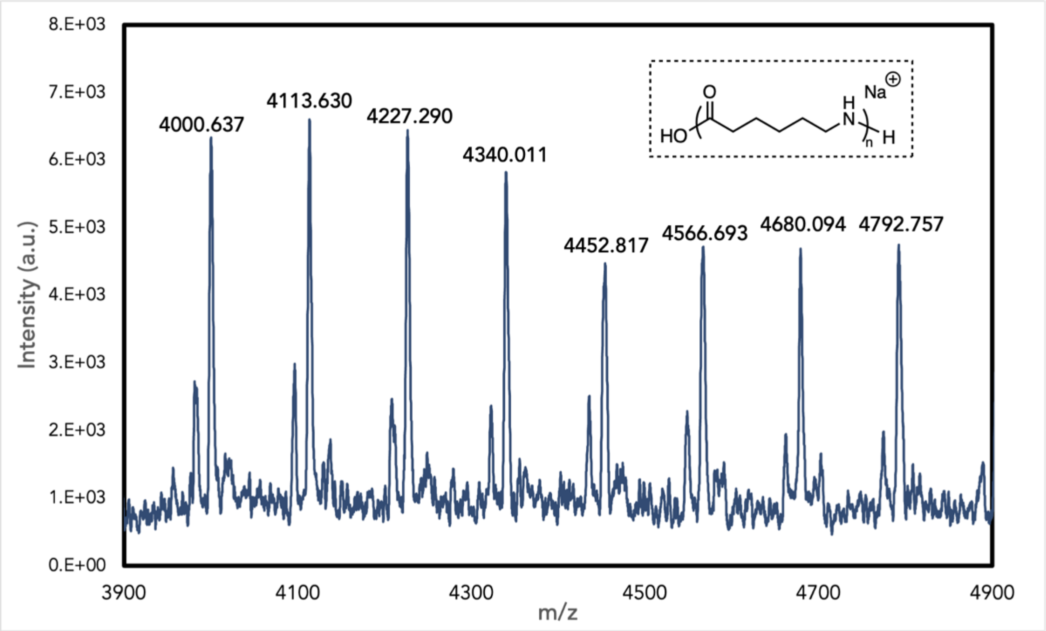

Figure S9. MALDI-TOF mass spectrum of purchased PA6 between 3900 and 4900 m/z. The spacing between peaks correlates well to the PA6 repeating unit mass (113 g/mol), with a combined cation and chain end mass of 40 g/mol (Carboxylic acid and amine chain end, 18 g/mol; Sodium cation, 22 g/mol).

Varied Carbon Black Loading for PA6 Photothermal Ring-Closing Depolymerization

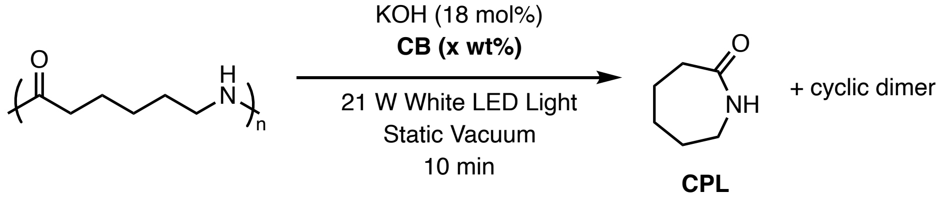

The procedure was slightly modified from the general PA6 ring-closing depolymerization procedure, where different loadings of CB were used while keeping the total reaction volume the same (86.5 mg). ^{1}H NMR analysis was performed in $CDCl_3$, and leftover PA6 was not quantified. The reaction workup was the same as the general PA6 photothermal depolymerization procedure. The depolymerization results are summarized below.

Table S2. Results of carbon black loading for PA6 photothermal ring-closing depolymerization.

Entry	CB (wt%)	CPL $Yield_{NMR}$(%)	Dimer $Yield_{NMR}$ (%)
1	0	0.0	0.0
2	1	48.3	1.4
3	2.5	74.1	3.0
4	5	70.1	1.8
5	10	71.4	2.3

each entry is an average of 2 trials

Control Experiments for PA6 Photothermal Ring-Closing Depolymerization

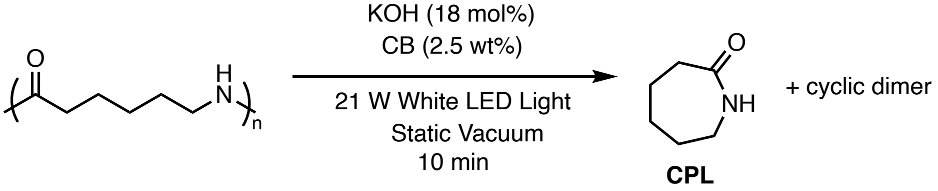

The procedure was slightly modified from the general PA6 ring-closing depolymerization procedure, and the deviations are listed below. ^{1}H NMR analysis was performed in $CDCl_3$, and leftover PA6 was not quantified. The reaction workup was the same as the general PA6 photothermal depolymerization procedure. The depolymerization results are summarized below.

For the foil-wrapped variation, the entire reaction vial was completely wrapped in aluminum foil prior to light irradiation. For the no light variation, the reaction vial was not subjected to LED light irradiation but rather left for 10 minutes in ambient laboratory light prior to workup. For entry 5, all reagents (PA6, KOH, and CB) were added to the reaction vial in a N_2 glovebox, the vial was tightly sealed and placed under static vacuum prior to light irradiation.

Table S3. Results of control PA6 photothermal ring-closing depolymerization experiments.

Entry	Variation	CPL $Yield_{NMR}$(%)	Dimer $Yield_{NMR}$ (%)
1	Foil Wrapped	0.0	0.0
2	No light	0.0	0.0
3	Air, instead of static vacuum	35.8	2.1
4	N_2, instead of static vacuum	27.7	0.9
5	Prepared in N_2 Glovebox	72.6	2.3

each entry is an average of 2 trials

Varied KOH Loading for PA6 Photothermal Ring-Closing Depolymerization

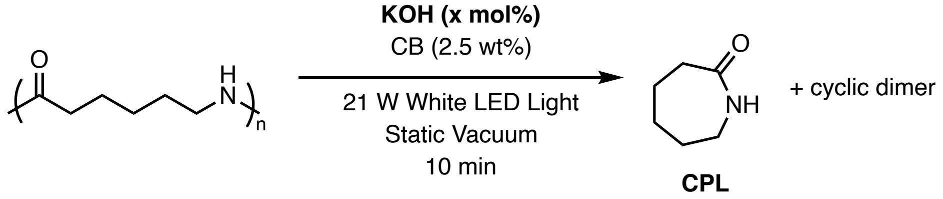

The procedure was slightly modified from the general PA6 ring-closing depolymerization procedure, where different loadings of KOH were used while keeping the total reaction volume the same (86.5 mg). ^{1}H NMR analysis was performed in $CDCl_3$, and leftover PA6 was not quantified. The reaction workup was the same as the general PA6 photothermal depolymerization procedure. The depolymerization results are summarized below.

Table S4. Results of KOH loading for PA6 photothermal ring-closing depolymerization.

Entry	KOH (mol%)	CPL $Yield_{NMR}$(%)	Dimer $Yield_{NMR}$ (%)
1	0	22.3	3.8
2	10	57.8	1.7
3	12	59.6	1.0
4	14	65.8	1.5
5	16	69.4	1.8
6	18	74.1	3.0
7	20	73.2	2.5
8	22	71.8	1.7
9	30	74.0	2.4

each entry is an average of 2 trials

Base Screen for PA6 Photothermal Ring-Closing Depolymerization

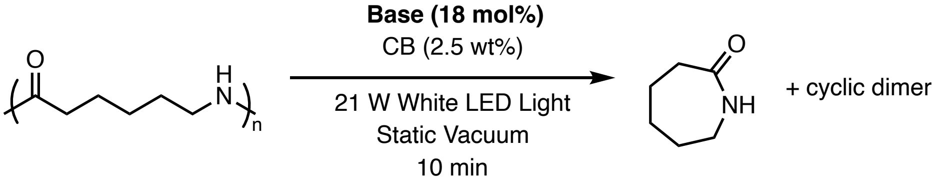

CPL

The procedure was the same as the general PA6 ring-closing depolymerization procedure, except that KO*t*-Bu , NaOH, NaO*t*-Bu, LiOH, LiO*t*-Bu, DBU, and TBD were used instead of KOH. ^{1}H NMR analysis was performed in $CDCl_3$, and leftover PA6 was not quantified. The reaction workup was the same as the general PA6 photothermal depolymerization procedure. The depolymerization results are summarized below.

Table S5. Results of base screen for PA6 photothermal ring-closing depolymerization.

Entry	Base	CPL $Yield_{NMR}$(%)	Dimer $Yield_{NMR}$ (%)
1	KOH	74.1	3.0
2	KO*t*-Bu	58.3	2.2
3	NaOH	68.0	2.9
4	NaO*t*-Bu	51.3	1.5
5	LiOH	56.5	1.8
6	LiO*t*-Bu	47.2	1.9
7	DBU	33.4	1.2
8	TBD	40.4	3.3

each entry is an average of 2 trials

Thermal Controls for PA6 Photothermal Ring-Closing Depolymerization

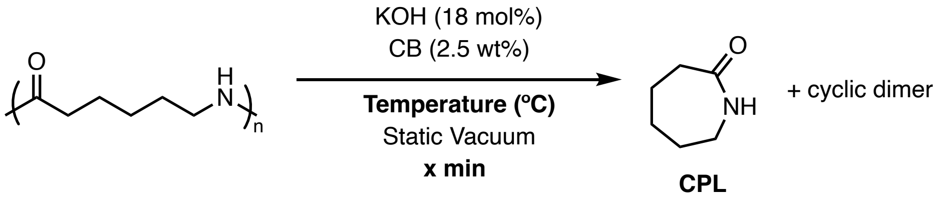

The procedure was modified from the general PA6 ring-closing depolymerization procedure, where the reaction proceeded via traditional thermal heating instead of photothermal conditions. A sand bath was preheated and equilibrated to 200, 300, 400, 450, 500, or 550 ºC, using a thermocouple to measure the temperature. Once the desired temperature stabilized, the reaction vial was inserted into the sand bath at a 2 cm depth for either 10 or 60 minutes. ^{1}H NMR analysis was performed in $CDCl_3$, and leftover PA6 was not quantified. The reaction workup was the same as the general PA6 photothermal depolymerization procedure. The depolymerization results are summarized below.

Table S6. Results of thermal PA6 ring-closing depolymerization.

Entry	Temperature (ºC)	Time (min)	CPL Yield$_{NMR}$(%)	Dimer Yield$_{NMR}$ (%)
1	200	60	0.2	0
2	300	60	6.0	0.3
3	400	60	29.5	0.8
4	400	10	7.0	0.4
5	450	10	29.7	1.6
6	500	10	59.2	1.9
7	550	10	67.3	1.9

each entry is an average of 2 trials

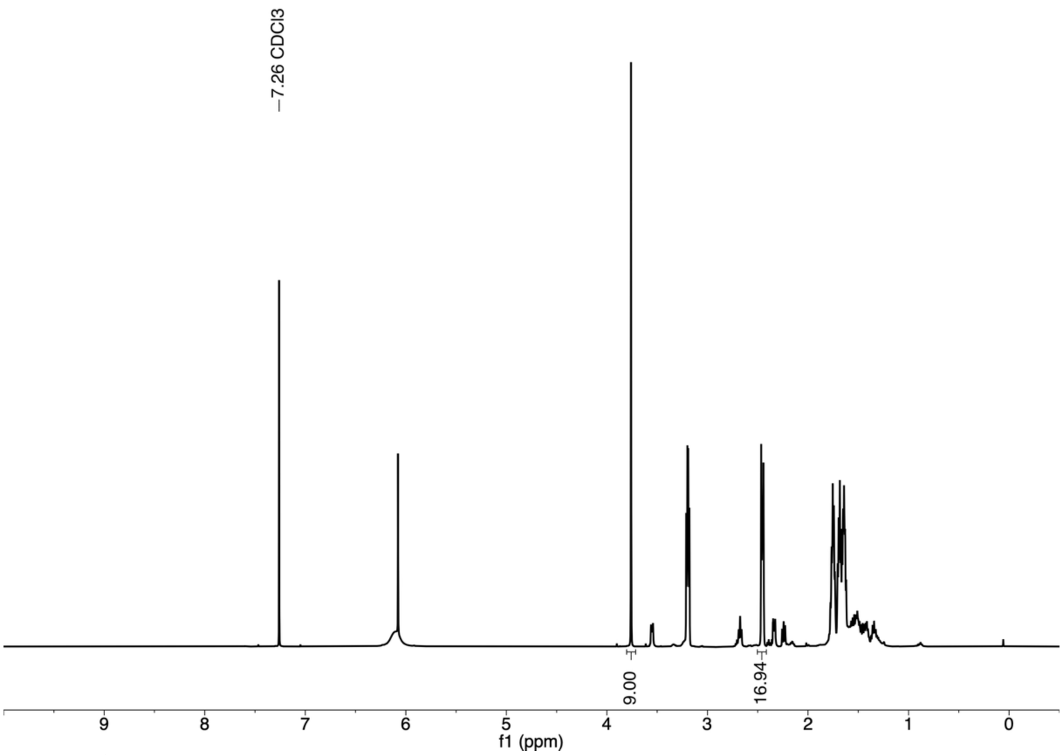

Figure S10. ^1H NMR of PA6 after thermal depolymerization at 400 ºC for 1 h.

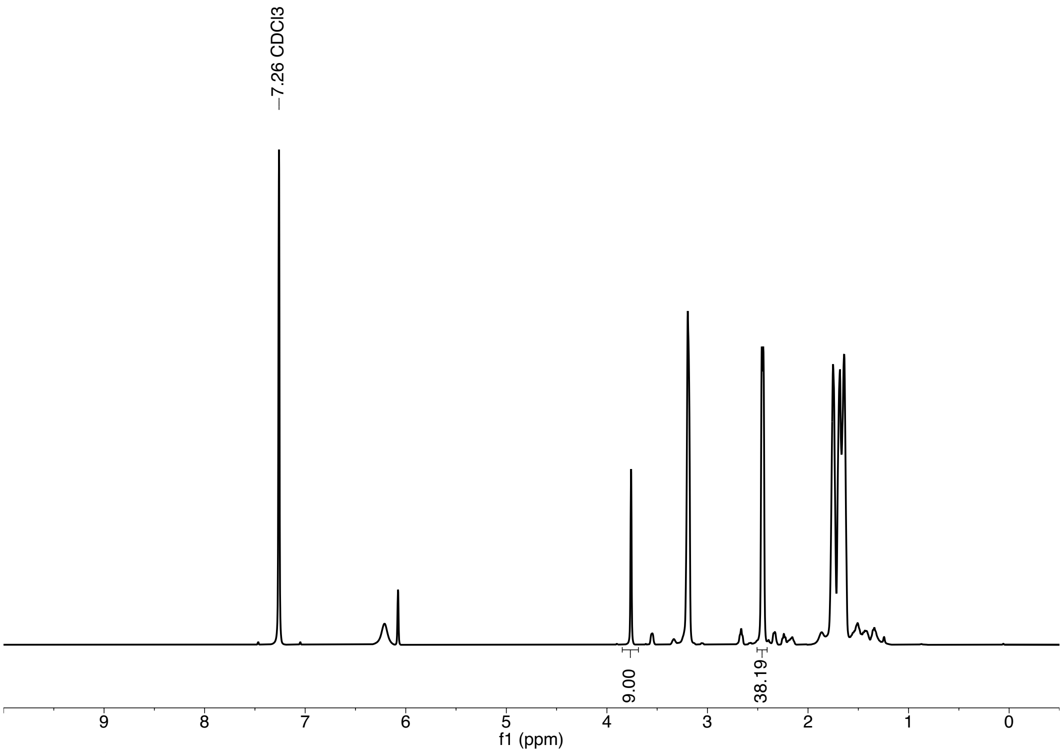

Figure S11. ^1H NMR of PA6 after thermal depolymerization at 550 ºC for 10 minutes.

Light Intensity Screen for PA6 Photothermal Ring-Closing Depolymerization

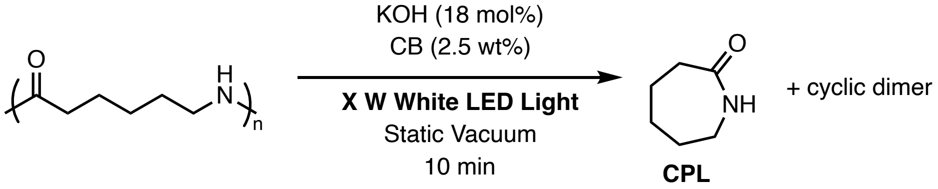

The procedure was the same as the general PA6 ring-closing depolymerization procedure, except that the light intensity was varied from 12 W to 21 W. ^{1}H NMR analysis was performed in $CDCl_3$, and leftover PA6 was not quantified. The reaction workup was the same as the general PA6 photothermal depolymerization procedure. The depolymerization results are summarized below.

Table S7. Results of light intensity screen for PA6 photothermal ring-closing depolymerization.

Entry	Light Intensity	CPL $Yield_{NMR}$(%)	Dimer $Yield_{NMR}$ (%)
1	12 W	43.4	1.8
2	14 W	58.8	2.4
3	16 W	56.9	1.3
4	18 W	60.1	1.4
5	21 W	74.1	3.0

each entry is an average of 2 trials

Large scale PA6 Photothermal Ring-Closing Depolymerization

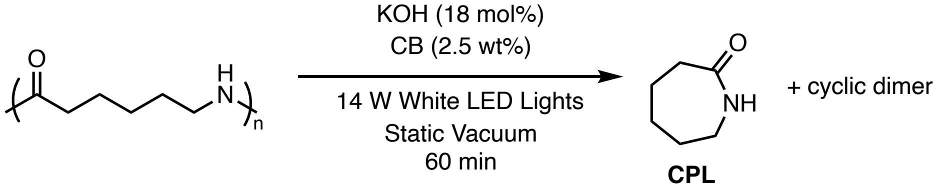

The procedure was modified from general PA6 ring-closing depolymerization procedure. For the 1-gram scale, a 20 mL scintillation vial fitted with a PTFE septa cap was used instead of a 1-dram vial. The reaction setup was also modified, where the vial was tilted at a 60° angle and used two 14 W lights (one on the bottom of the vial, and one on pointing downwards on the vial side) instead of just one 21 W light at the bottom of the vial. The reaction time was also modified to be 60 minutes instead of 10 minutes. ^1H NMR analysis was performed in $CDCl_3$, and leftover PA6 was not quantified. The reaction workup was the same as the general PA6 photothermal depolymerization procedure. The depolymerization results are summarized below.

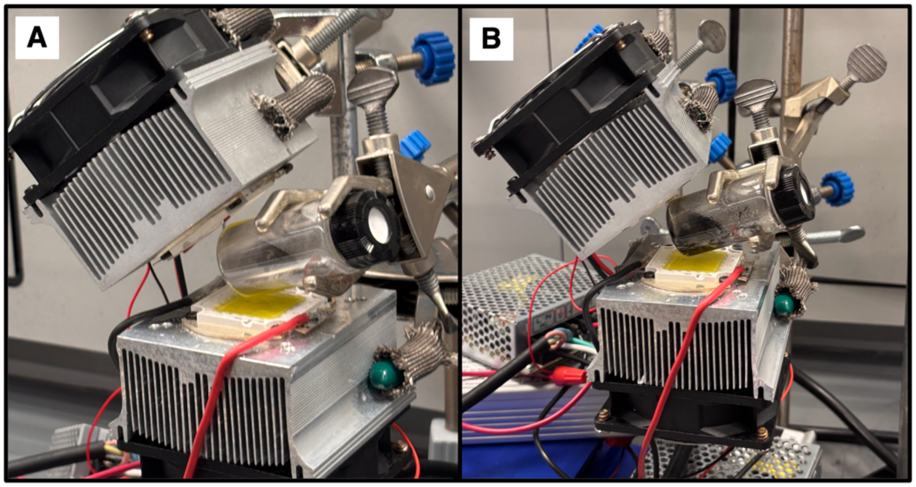

Figure S12. Reaction setup of large scale PA6 ring-closing photothermal depolymerization. A) Before light irradiation. B) After light irradiation.

Table S8. Results of large scale PA6 photothermal ring-closing depolymerization.

Entry	Variation	CPL Yield$_{NMR}$(%)	Dimer Yield$_{NMR}$ (%)
1	1 g scale	62.2	2.5

each entry is an average of 2 trials

PA6 Mechanistic Studies

14 W Light Intensity Kinetics for PA6 Photothermal Ring-Closing Depolymerization

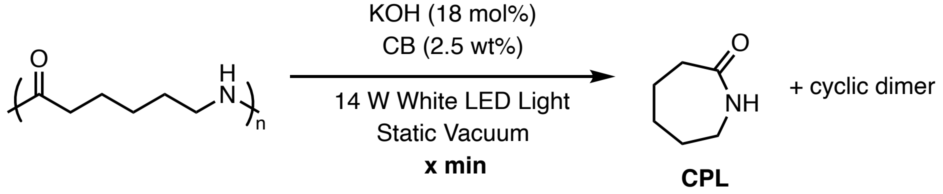

The procedure was the same as the general PA6 depolymerization procedure, where the light intensity was adjusted to 14 W and that the depolymerization time was varied from 0 minutes to 60 minutes. ^{1}H NMR analysis was performed in $CDCl_3$, and leftover PA6 was not quantified. The reaction workup was the same as the general PA6 photothermal depolymerization procedure. The depolymerization results are summarized below.

Table S9. Results of 14 W kinetics for PA6 photothermal ring-closing depolymerization.

Entry	Time (min)	CPL $Yield_{NMR}$(%)	Dimer $Yield_{NMR}$ (%)
1	0	0.0	0.0
2	1	0.1	0.0
3	2.5	37.4	0.1
4	5	51.5	1.3
5	7.5	58.0	2.6
6	10	58.8	2.4
7	12.5	60.2	2.2
8	15	64.0	2.4
9	20	63.6	2.4
10	30	64.1	2.5
11	60	64.2	2.5

Each entry is an average of 3 trials.

Gel permeation chromatography (GPC) is a type of chromatography that separates polymer chains by their size in solution, where the refractive index detector measures the relative concentration of the eluted separated sample over time. Refractive index signals at a higher retention time indicate polymer chains that have a smaller hydrodynamic volume, and vice versa. While hydrodynamic volume does not necessarily equate to molecular weight, the use of calibration standards, and in this case, narrow dispersity PMMA standards, allows for the comparison of molecular weight with hydrodynamic radius. GPC was used to determine the molecular weight distribution of the polymer, and more specifically how polymer length decreased as the reaction progressed.

To determine the molecular weight distribution as photothermal depolymerization proceeded, $CDCl_3$ was removed through evaporation from each reaction vial after aliquots were taken for NMR analysis. Once dry, the solid residues were redissolved in HPLC-grade HFIP (2 mL) and vortexed well to mix. The reaction mixture was passed through a cotton plug and a 0.22 µm PTFE syringe filter, and aliquots were taken for Gel Permeation Chromatography (GPC) analysis. The GPC results are summarized below.

Table S10. GPC results of PA6 photothermal ring-closing depolymerization at 14 W.

Entry	Time (min)	M_n (kg/mol)	Đ
1	0	24.1	1.70
2	1	22.4	1.70
3	2.5	2.2	7.59
4	5	1.6	1.86
5	7.5	1.5	1.67
6	10	1.4	1.72
7	30	1.3	1.60
8	60	1.4	1.55

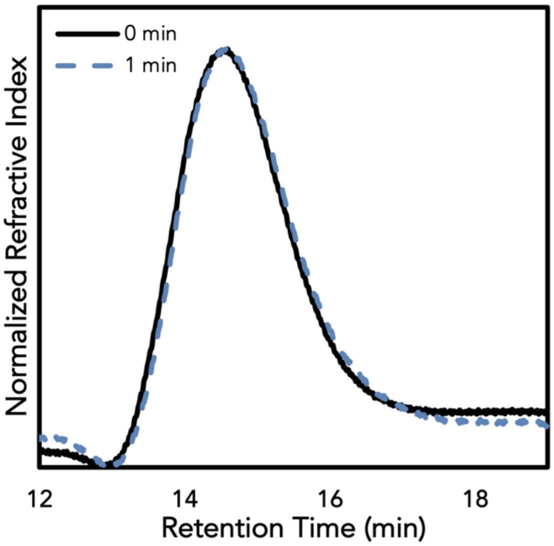

Figure S13. GPC of PA6 after 1 minute of photothermal depolymerization at 14 W light intensity.

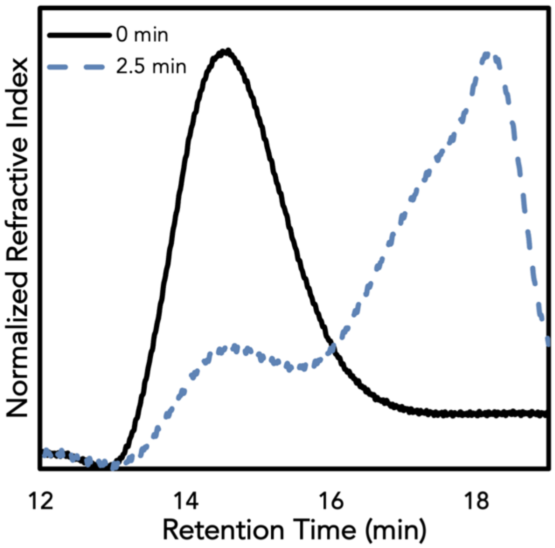

Figure S14. GPC of PA6 after 2.5 minutes of photothermal depolymerization at 14 W light intensity.

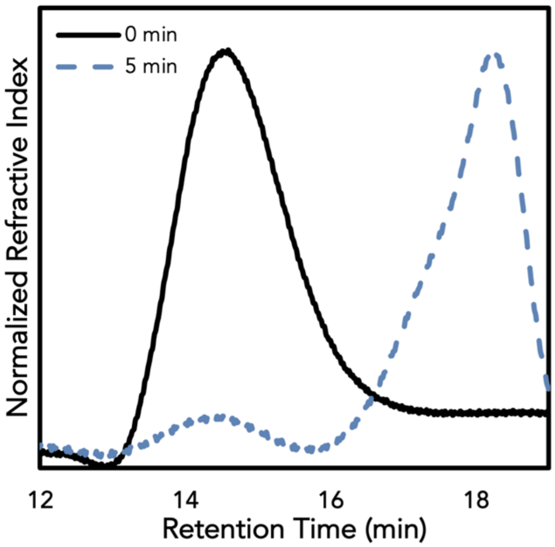

Figure S15. GPC of PA6 after 5 minutes of photothermal depolymerization at 14 W light intensity.

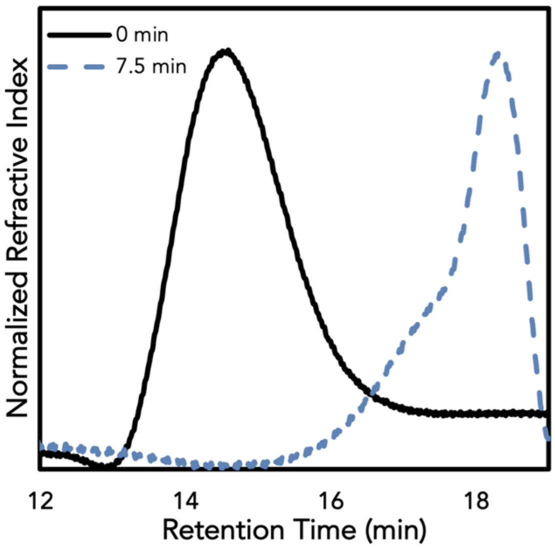

Figure S16. GPC of PA6 after 7.5 minutes of photothermal depolymerization at 14 W light intensity.

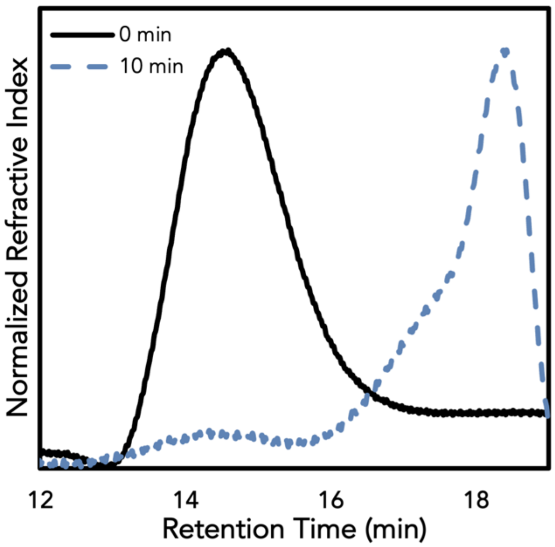

Figure S17. GPC of PA6 after 10 minutes of photothermal depolymerization at 14 W light intensity.

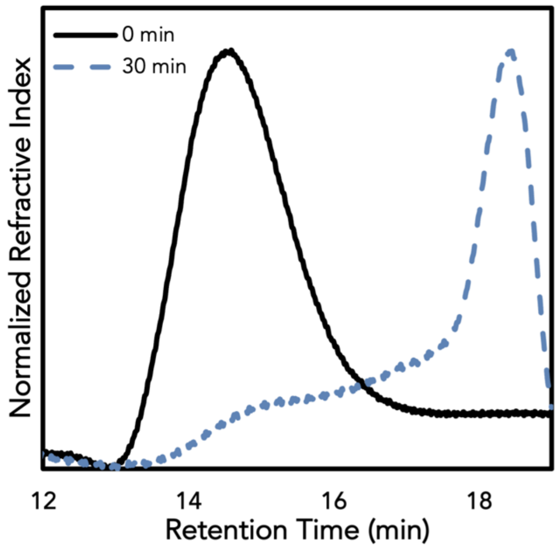

Figure S18. GPC of PA6 after 30 minutes of photothermal depolymerization at 14 W light intensity.

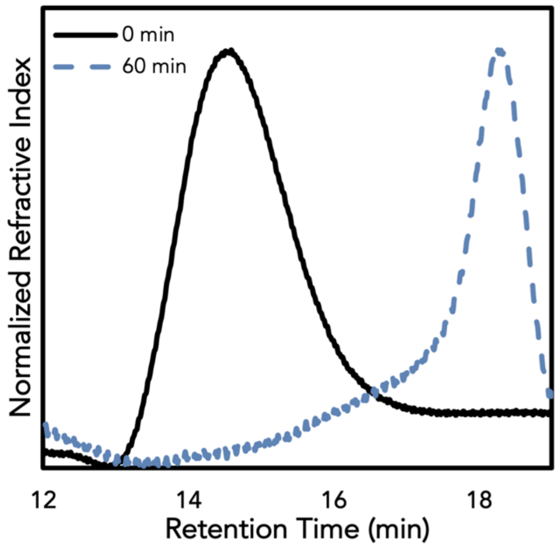

Figure S19. GPC of PA6 after 60 minutes of photothermal depolymerization at 14 W light intensity.

21 W Light Intensity Kinetics for PA6 Photothermal Ring-Closing Depolymerization

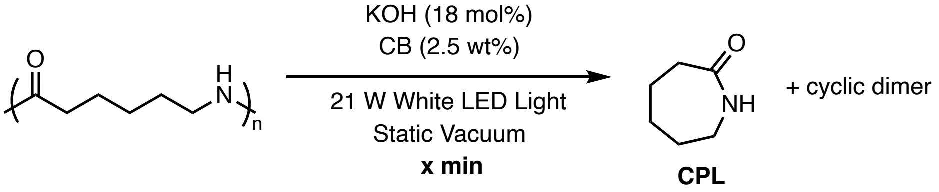

The procedure was the same as the general PA6 depolymerization procedure, except that the depolymerization time was varied from 0 minutes to 15 minutes. ^{1}H NMR analysis was performed in $CDCl_3$, and leftover PA6 was not quantified. The reaction workup was the same as the general PA6 photothermal depolymerization procedure. The depolymerization results are summarized below. An aliquot of the 10-minute time point was also taken for GPC analysis using the same procedure outlined in the 14 W light intensity kinetics procedure.

Table S11. Results of 21 W Kinetics for PA6 photothermal ring-closing depolymerization.

Entry	Time (min)	CPL Yield$_{NMR}$(%)	Dimer Yield$_{NMR}$ (%)
1	0	0.0	0.0
2	1	41.8	1.1
3	2	53.7	1.7
4	3	66.1	2.1
5	4	64.9	2.3
6	5	66.7	3.3
7	7.5	70.0	2.1
8	10	74.1	3.0
9	15	72.8	3.1

each entry is an average of 3 trials

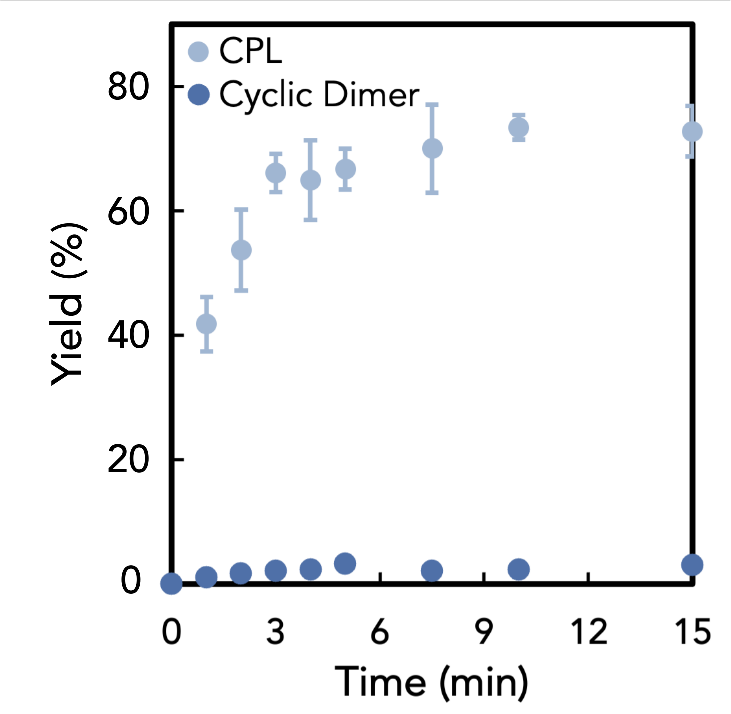

Figure S20. Scatter plot of PA6 photothermal depolymerization products over time using 21 W light intensity. Error bars represent the standard deviation across three trials.

Table S12. GPC results of PA6 after photothermal ring-closing depolymerization at 21 W.

Entry	Time (min)	M_n (kg/mol)	Đ
1	0	24.1	1.70
2	10	1.2	1.32

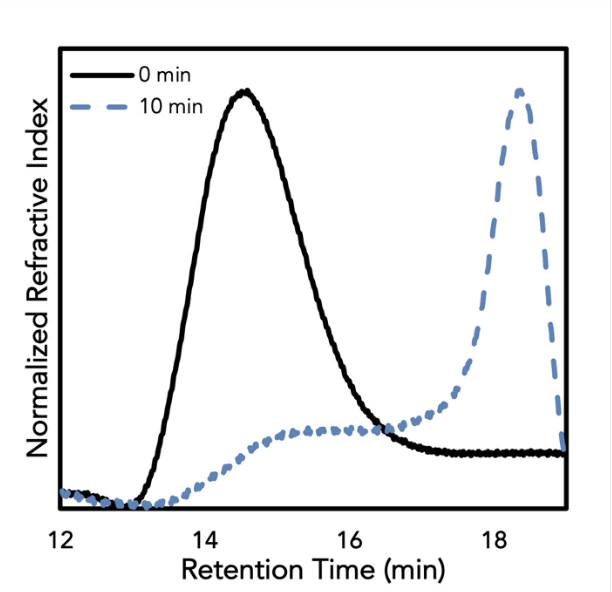

Figure S21. GPC of PA6 after 10 minutes of photothermal depolymerization at 21 W light intensity.

Anionic Ring-Opening Polymerization for AcPA6 synthesis

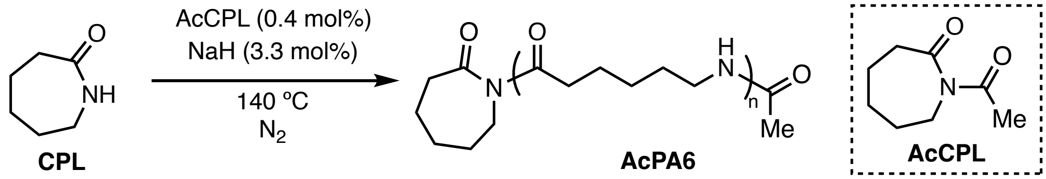

PA6 was synthesized *via* anionic ring opening polymerization, using a slightly modified version of previously reported methods.[2,3] CPL (400 mg, 3.53 mmol), NaH (4.7 mg, 60% dispersion in mineral oil, 0.12 mmol), AcCPL (1.8 µL, 1.27×10^{-2} mmol), and a small stir bar were added to a 2-dram glass vial fitted with a PTFE septa cap in a nitrogen-filled glovebox. The vial was tightly sealed and transferred out of the glovebox. The vial was then placed in a preheated 140 ºC pie block and stirring was started. Once the contents of the reaction vial solidified at 140 ºC (~10 minutes), the reaction was stopped and quickly cooled using a dry ice-acetone bath. The vial was allowed to thaw to room temperature, and 3:1 TFE/$CDCl_3$ (4 mL) was added and mixed well to dissolve polymer. A small aliquot was taken for crude ^{1}H NMR analysis. The remaining polymerization reaction mixture was precipitated in cold MeOH (150 mL) and the precipitate was collected and dried under vacuum overnight. The resulting solid polymer product was weighed (310.1 mg, 2.74 mmol) and characterized using ^{1}H and ^{13}C NMR analysis in 3:1 TFE/$CDCl_3$ and MALDI-TOF MS. Purity was measured through ^{1}H NMR analysis against a $DMSO_2$ internal standard.

Table S13. Results of anionic ring-opening polymerization for PA6 synthesis.

Entry	CPL (mg)	Conversion (%)	Isolated PA6 (mg)	Purity (%)
1	400	94.3	310.1	98.8

Conversion was measured using the ratio between monomer signal and PA6 signal in the crude ^{1}H NMR.

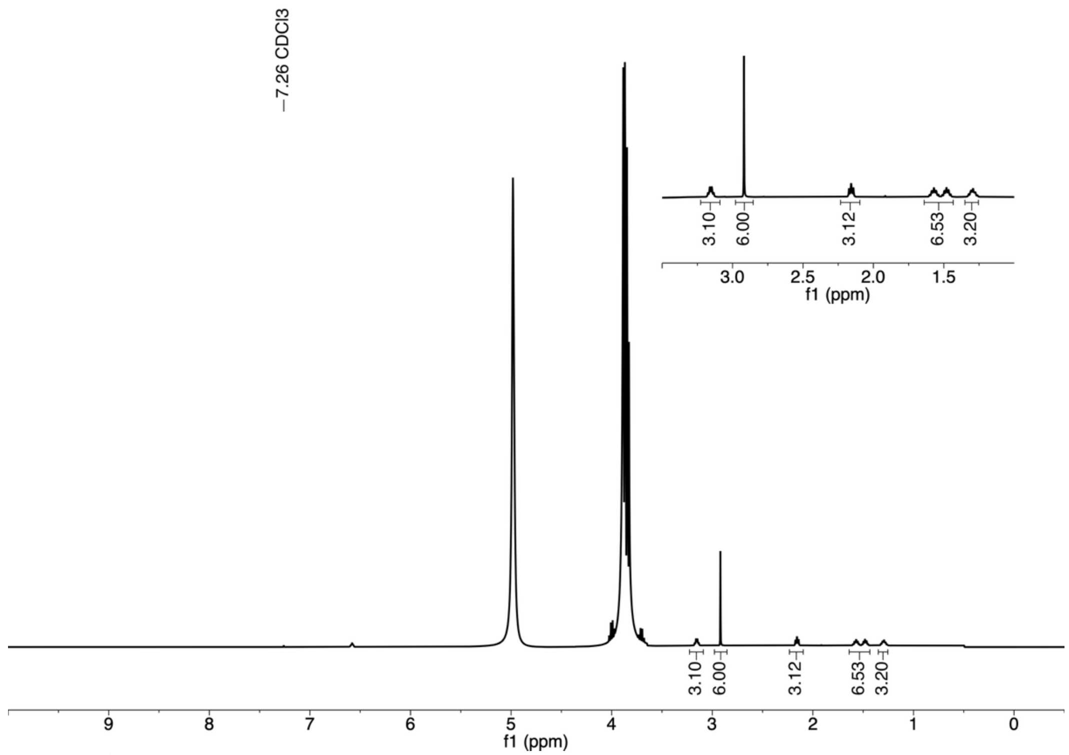

Figure S22. ^{1}H NMR of synthesized PA6 *via* anionic ring opening polymerization. The spectrum was baseline corrected between the 3.5 to 0.5 ppm region to ensure that protonated TFE signals appearing at 3.8 ppm and 4.9 ppm would not interfere with integrations. $DMSO_2$ signal appears at 2.9 ppm.

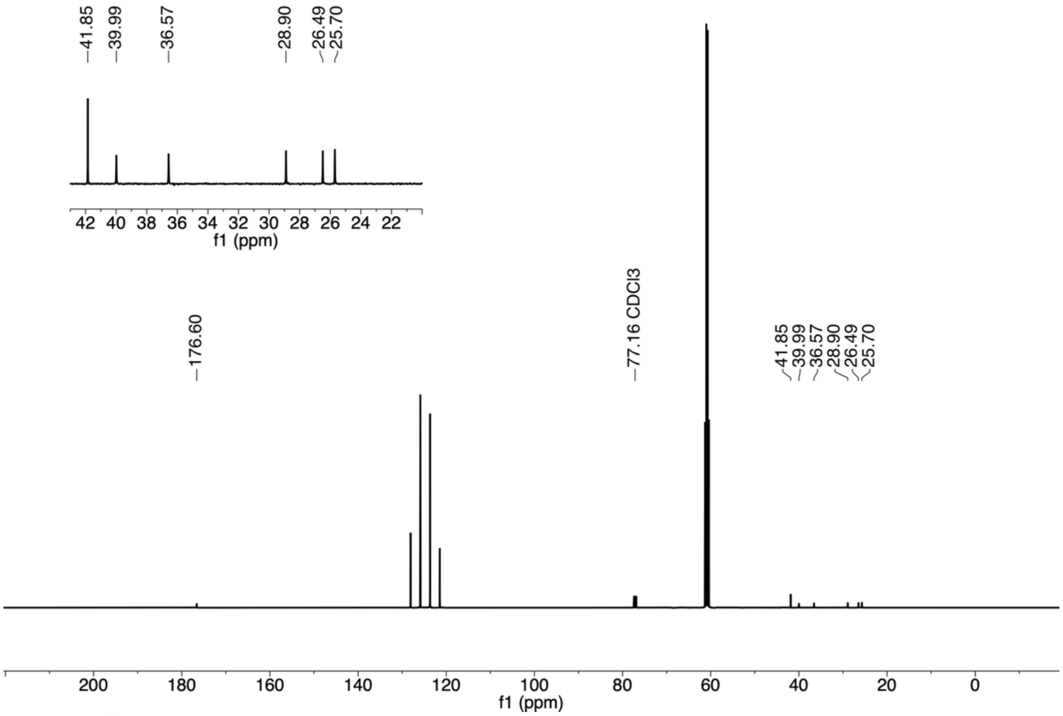

Figure S23. ^{13}C NMR of synthesized PA6 *via* anionic ring opening polymerization. TFE peaks can be seen at 124 and 60 ppm.

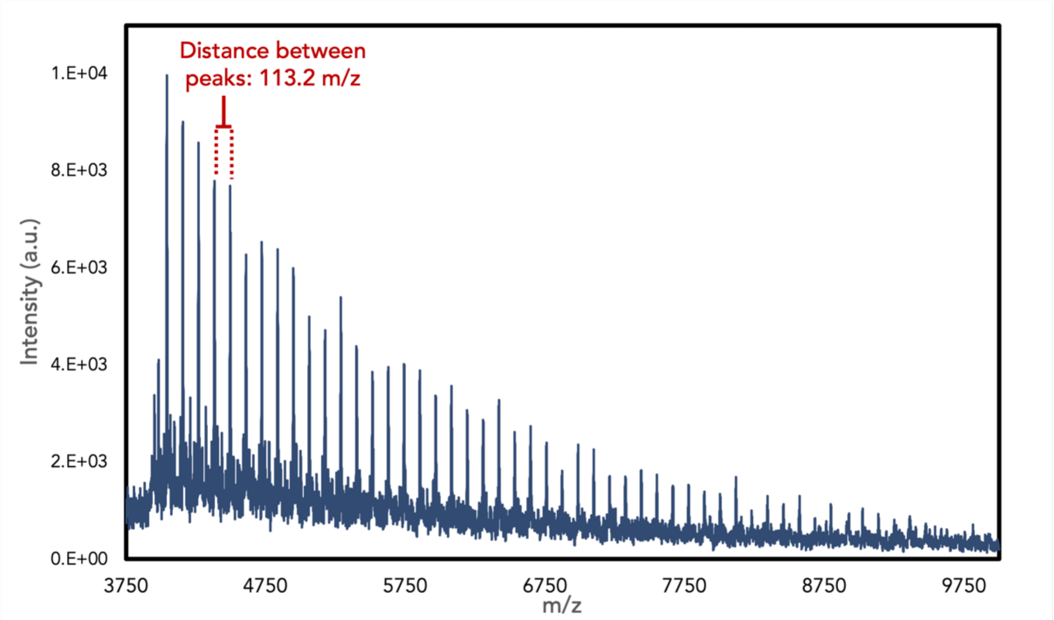

Figure S24. MALDI-TOF mass spectrum of synthesized PA6 *via* anionic ring opening polymerization between 3750 and 10000 m/z (signal suppression up to 4000 m/z). The spacing between peaks correlates well to the PA6 repeating unit mass (113.2 g/mol).

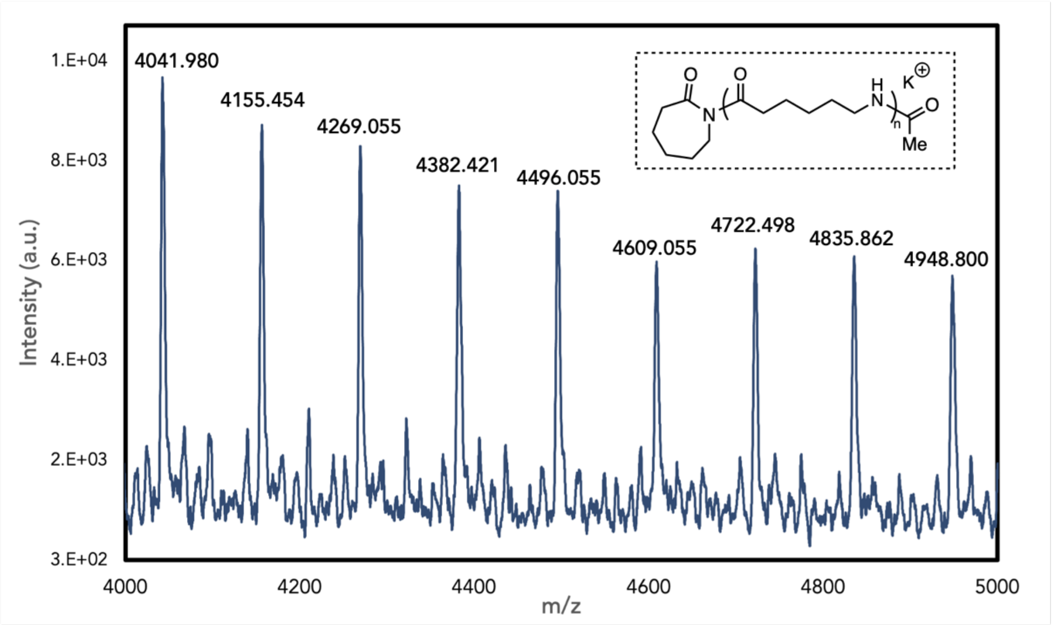

Figure S25. MALDI-TOF mass spectrum of synthesized PA6 *via* anionic ring opening polymerization between 4000 and 5000 m/z. The spacing between peaks correlates well to the PA6 repeating unit mass (113.2 g/mol), with a combined cation and chain end mass of 82 g/mol (Acetyl chain end, 43 g/mol; Potassium cation, 39 g/mol; Caprolactam chain end, 113 g/mol, is accounted for in the repeating units).

AcPA6 Photothermal Ring-Closing Depolymerization using Optimized Conditions

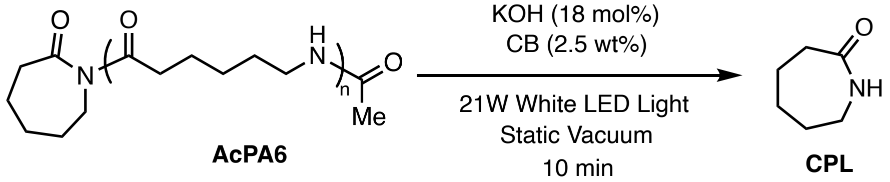

The procedure was slightly modified from the general PA6 ring-closing depolymerization procedure, where the synthesized PA6 with the acetyl chain (AcPA6) end was used instead of the commercially purchased PA6 powder. ^{1}H NMR analysis was performed in $CDCl_3$, and leftover PA6 was not quantified. The reaction workup was the same as the general PA6 photothermal depolymerization procedure. The depolymerization results are summarized below.

Table S14. Results of synthesized PA6 with acetyl chain end after photothermal ring-closing depolymerization.

Entry	CPL Yield$_{NMR}$(%)	Dimer Yield$_{NMR}$ (%)
1	69.7 (± 3.1)	1.8 (± 0.2)

Each entry is an average of 2 trials. Error represented is the standard deviation between trials.

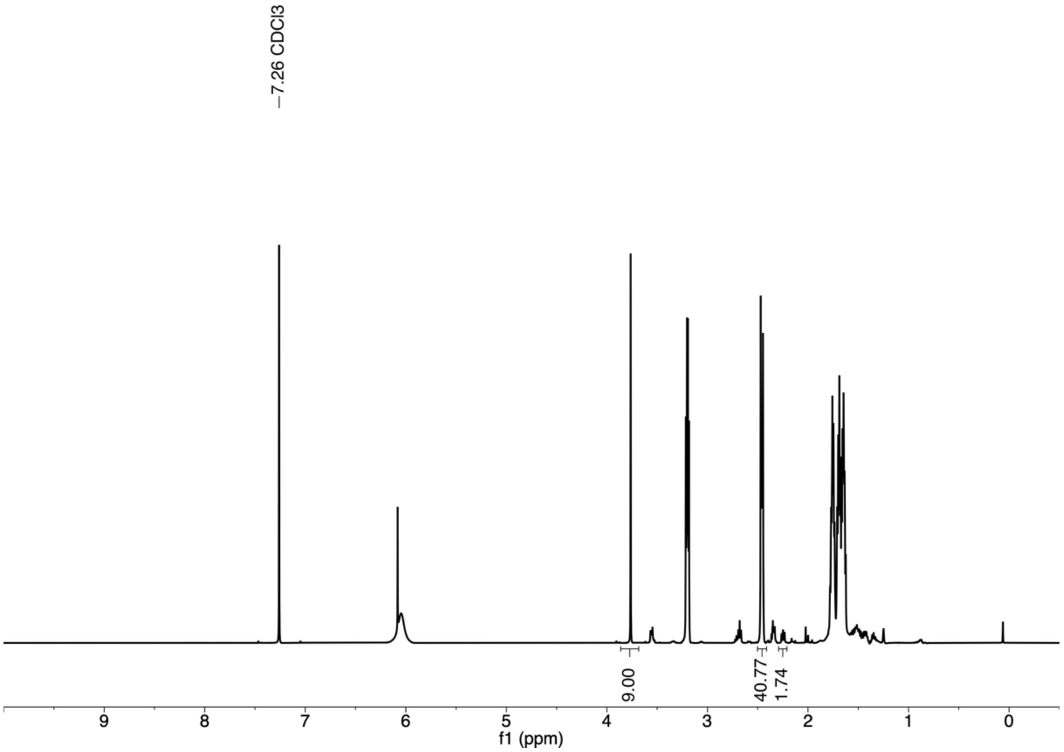

Figure S26. ^{1}H NMR of AcPA6 after photothermal ring-closing depolymerization using optimized depolymerization conditions.

AcPA6 Photothermal Ring-Closing Depolymerization using 14 W Light intensity

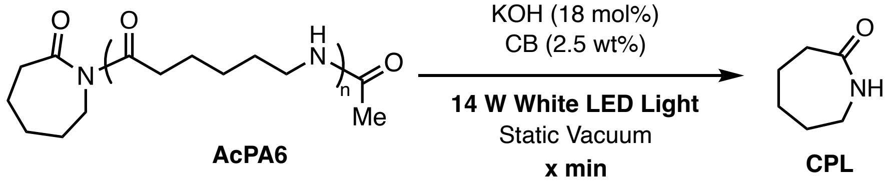

The procedure was slightly modified from the general PA6 ring-closing depolymerization procedure, where the AcPA6 was used instead of the commercially purchased PA6 powder. The light intensity was adjusted to 14 W, and the reaction time was varied to be either 2.5, 5, 7.5, or 30 minutes. ^{1}H NMR analysis was performed in $CDCl_3$, and leftover PA6 was not quantified. The remaining AcPA6 in the 2.5-minute and 5-minute reactions was characterized using MALDI-TOF MS to confirm chain end fidelity during the photothermal depolymerization reaction. The reaction workup was the same as the general PA6 photothermal depolymerization procedure. The depolymerization results are summarized below.

To ensure all starting material for the 14 W kinetic reactions were the same, the AcPA6 used as starting material was synthesized using the same procedure outlined in the section entitled "Anionic Ring-Opening Polymerization for AcPA6 synthesis" in a larger scale (8.84 mmol CPL, 77.1% conversion, 97.1% purity, 742.1 mg isolated polymer). The AcPA6 starting material was characterized *via* ^{1}H NMR and MALDI-TOF MS shown below.

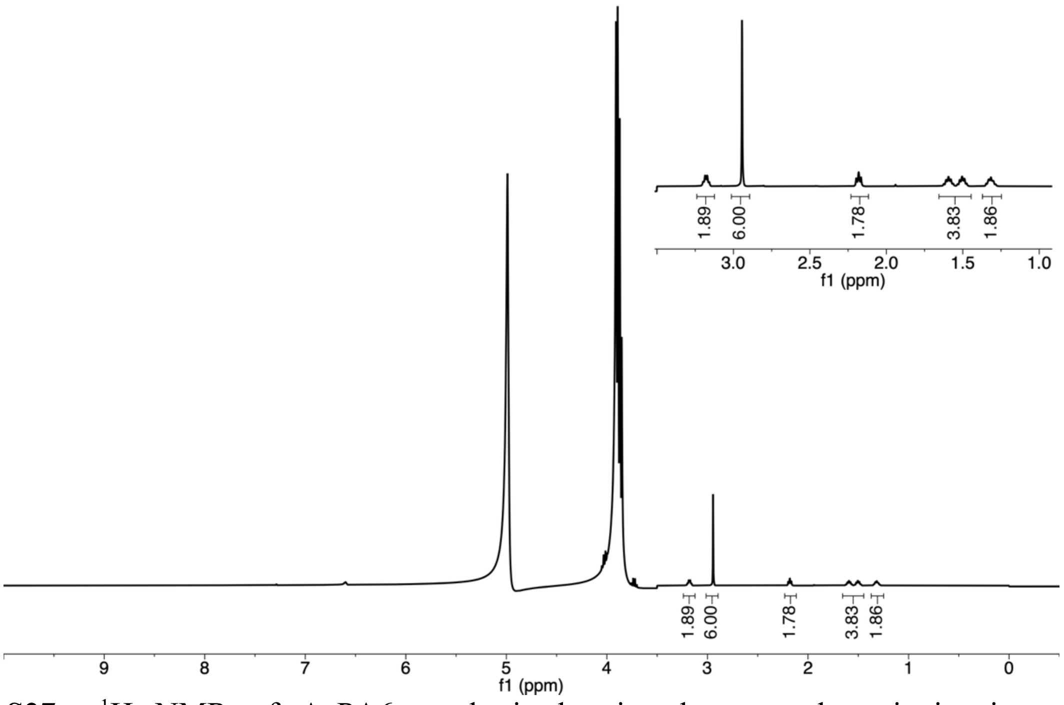

Figure S27. ^{1}H NMR of AcPA6 synthesized using larger scale anionic ring opening polymerization. The spectrum was baseline corrected between the 3.5 to 0 ppm region to ensure that protonated TFE signals appearing at 3.8 ppm and 4.9 ppm would not interfere with integrations.

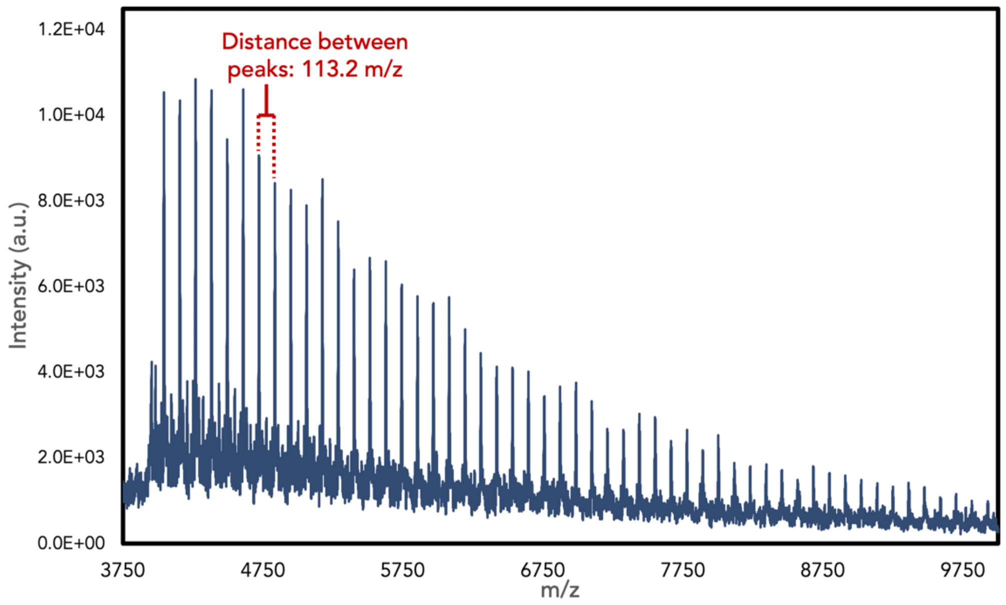

Figure S28. MALDI-TOF mass spectrum of AcPA6 synthesized *via* larger scale anionic ring opening polymerization between 3750 and 10000 m/z (signal suppression up to 4000 m/z). The spacing between peaks correlates well to the PA6 repeating unit mass (113.2 g/mol).

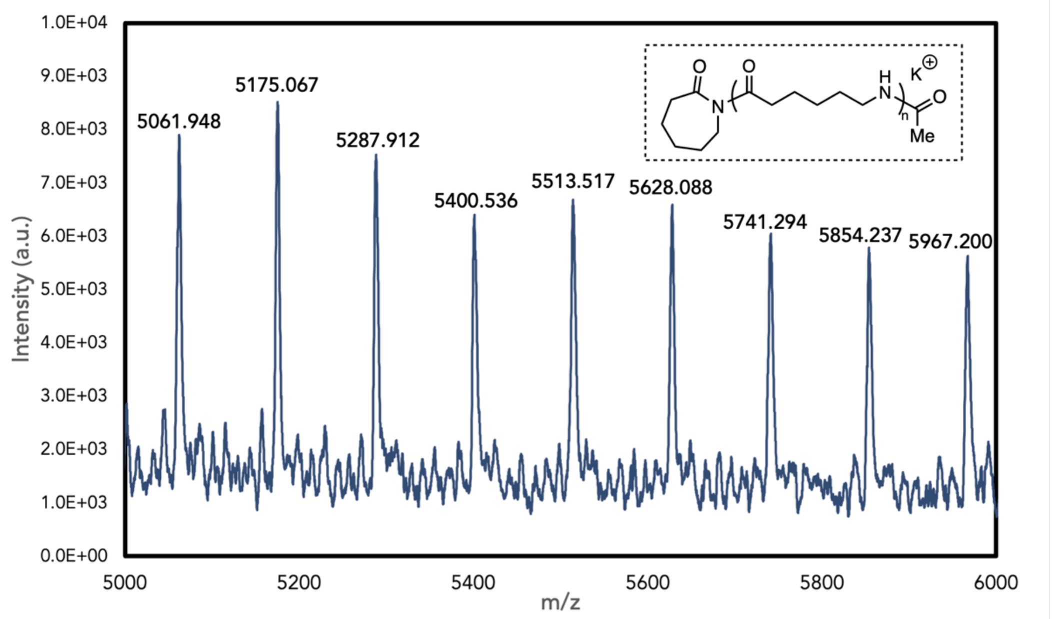

Figure S29. MALDI-TOF mass spectrum of AcPA6 synthesized *via* larger scale anionic ring opening polymerization between 5000 and 6000 m/z. The spacing between peaks correlates well to the PA6 repeating unit mass (113.2 g/mol), with a combined cation and chain end mass of 82 g/mol (Acetyl chain end, 43 g/mol; Potassium cation, 39 g/mol; Caprolactam chain end, 113 g/mol, is accounted for in the repeating units).

Table S15. Results of AcPA6 after photothermal ring-closing depolymerization kinetics study with 14 W light intensity.

Entry	Time (min)	CPL Yield$_{NMR}$(%)	Dimer Yield$_{NMR}$ (%)
1	2.5	31.4 (± 4.2)	0.8 (± 0.5)
2	5	47.6 (± 3.5)	1.8 (± 4.0)
3	7.5	58.6 (± 2.5)	2.3 (± 0.7)
4	30	61.9 (± 2.8)	1.9 (± 1.1)

Each entry is an average of 2 trials. Error represented is the standard deviation between trials.

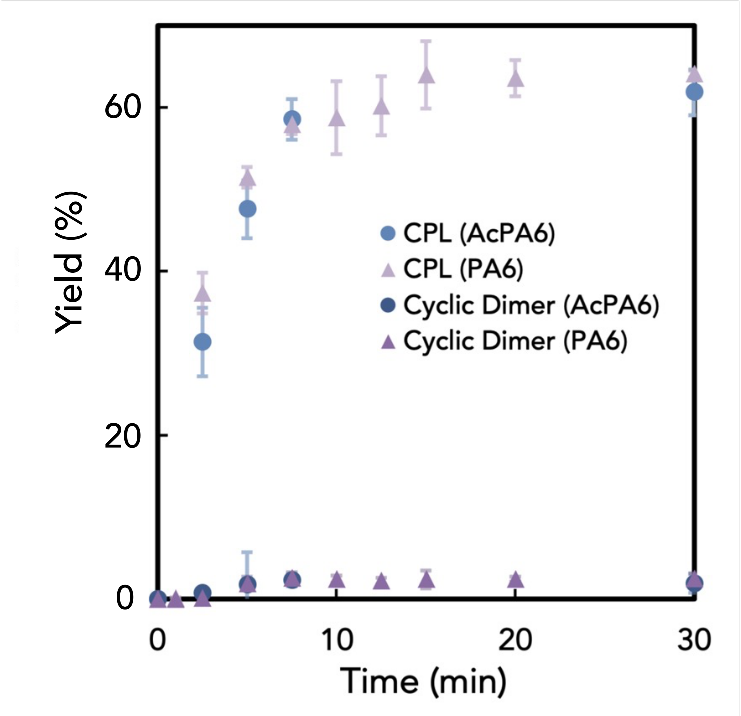

Figure S30. Scatter plot of AcPA6 and purchased PA6 photothermal depolymerization products over time using 14 W light intensity. Triangle markers are representative of purchased PA6 depolymerization products, and circle markers are representative of AcPA6 depolymerization products. Error bars represent the standard deviation across two or three trials for AcPA6 and PA6 data points, respectively.

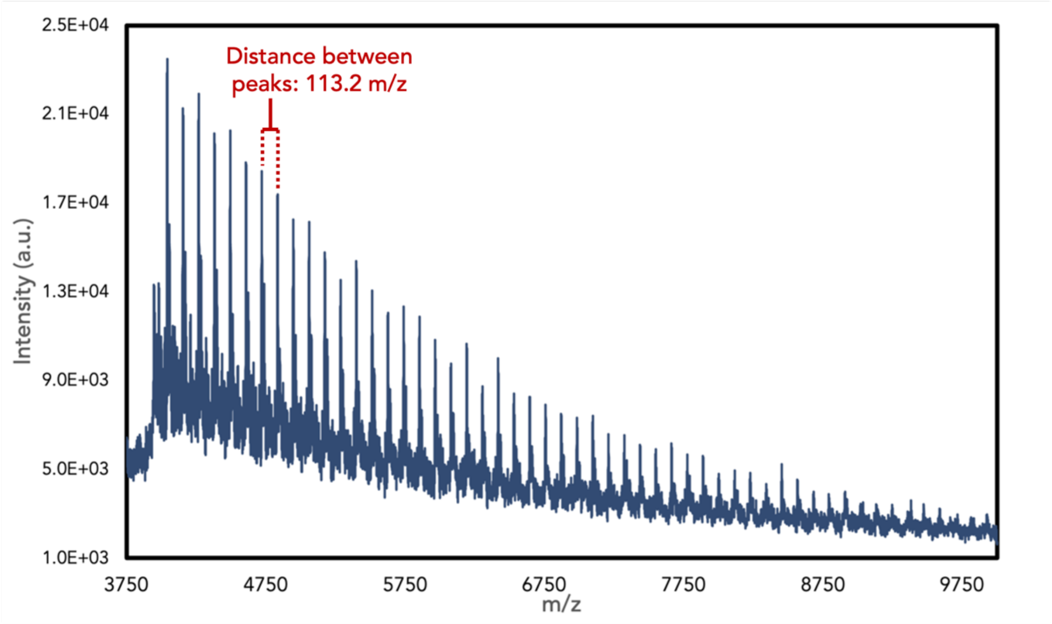

Figure S31. MALDI-TOF mass spectrum of AcPA6 after 2.5 minutes of photothermal depolymerization using 14 W light between 3750 and 10000 m/z (signal suppression up to 4000 m/z). The spacing between peaks correlates well to the PA6 repeating unit mass (113.2 g/mol).

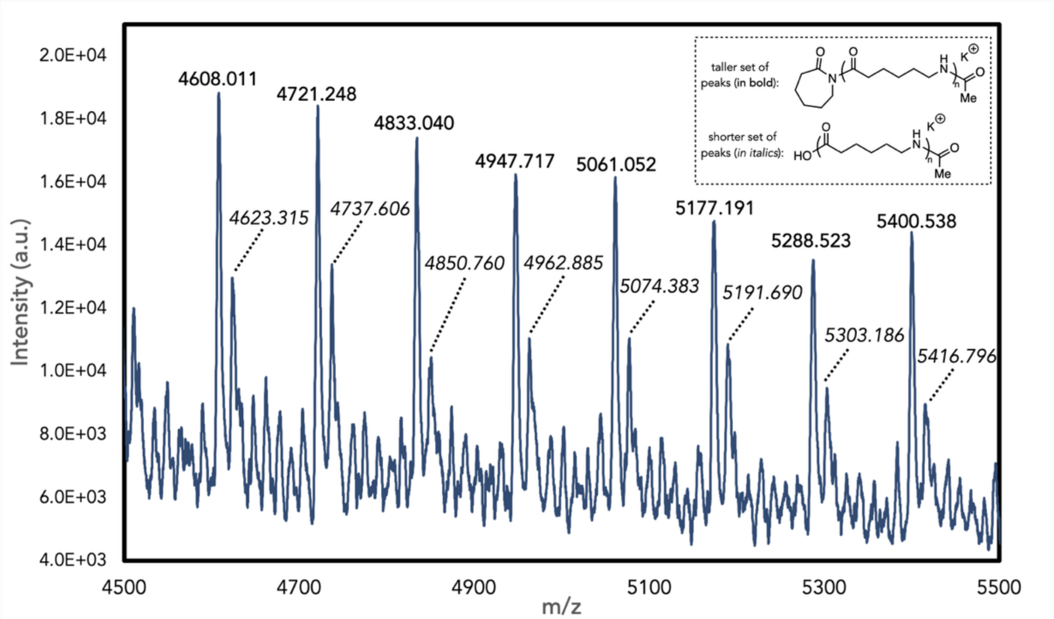

Figure S32. MALDI-TOF mass spectrum of AcPA6 after 2.5 minutes of photothermal depolymerization using 14 W light between 4500 and 5500 m/z. The spacing between peaks correlates well to the PA6 repeating unit mass (113.2 g/mol). The first series of peaks (bolded) have a combined cation and chain end mass of 82 g/mol (Acetyl chain end, 43 g/mol; Potassium cation, 39 g/mol; Caprolactam chain end, 113 g/mol, is accounted for in the repeating units). The second series of peaks (italicized) have a combined cation and chain end mass of 99 g/mol (Acetyl chain end, 43 g/mol; Potassium cation, 39 g/mol; carboxylic acid chain end, 17 g/ mol).

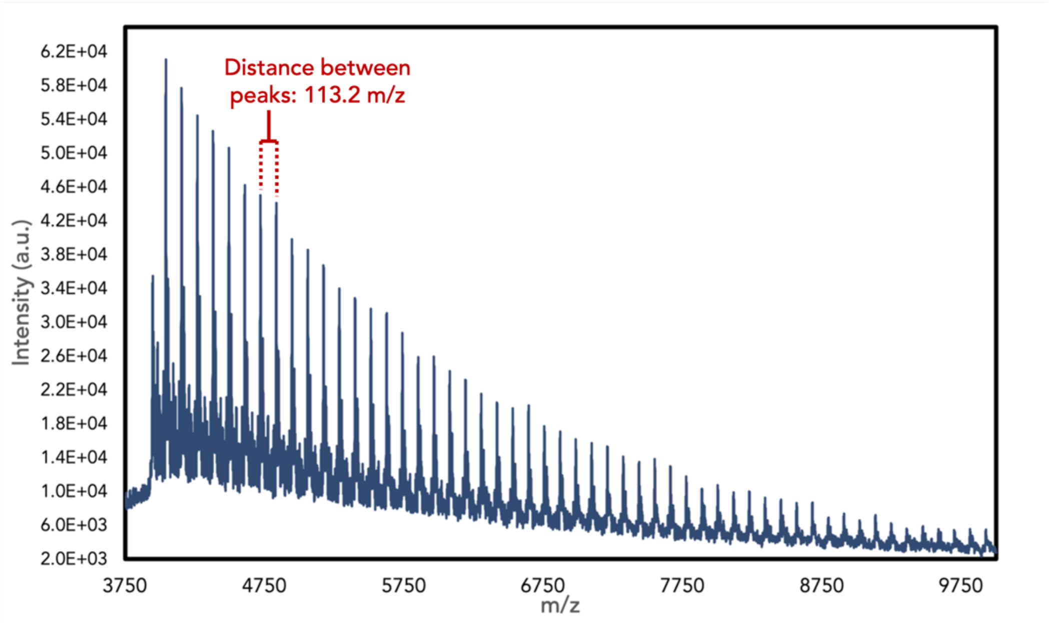

Figure S33. MALDI-TOF mass spectrum of AcPA6 after 5 minutes of photothermal depolymerization using 14 W light between 3750 and 10000 m/z (signal suppression up to 4000 m/z). The spacing between peaks correlates well to the PA6 repeating unit mass (113.2 g/mol).

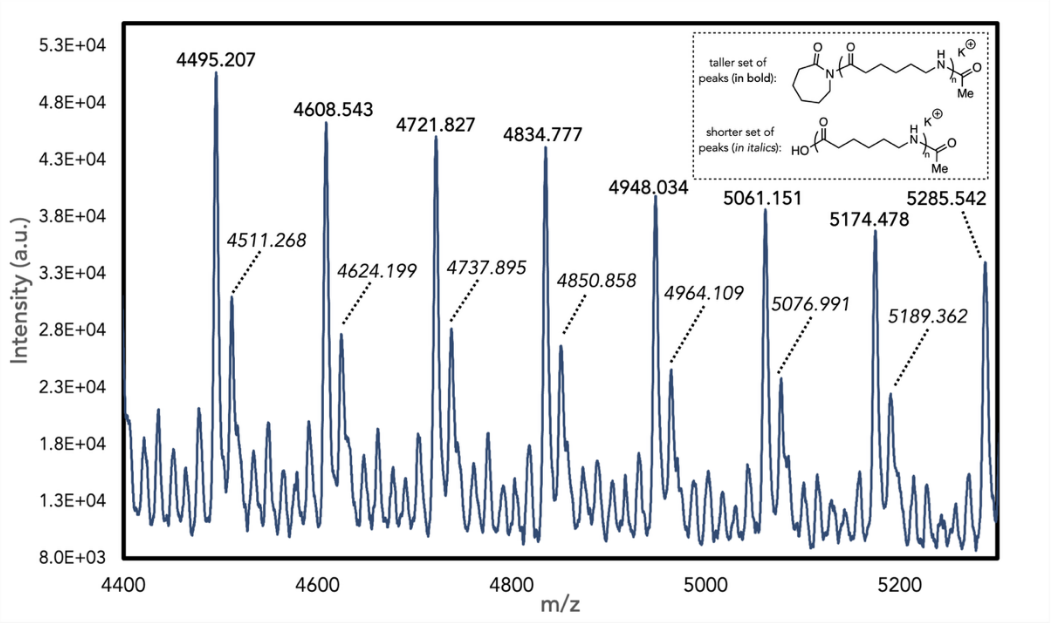

Figure S34. MALDI-TOF mass spectrum of AcPA6 after 5 minutes of photothermal depolymerization using 14 W light between 4400 and 5300 m/z. The spacing between peaks correlates well to the PA6 repeating unit mass (113.2 g/mol). The first series of peaks (bolded) have a combined cation and chain end mass of 82 g/mol (Acetyl chain end, 43 g/mol; Potassium cation, 39 g/mol; Caprolactam chain end, 113 g/mol, is accounted for in the repeating units). The second series of peaks (italicized) have a combined cation and chain end mass of 99 g/mol (Acetyl chain end, 43 g/mol; Potassium cation, 39 g/mol; carboxylic acid chain end, 17 g/ mol).

From the MALDI-TOF MS results after 2.5- and 5- minutes of photothermal depolymerization, we observed two series of peaks corresponding to PA6 with different chain ends. The dominant series of peaks in both samples can be assigned to AcPA6 as synthesized, with a cyclic CPL on one chain end and the acetylated amine on the other chain end. The minor series of peaks in both samples corresponds to AcPA6 with a carboxylic acid chain end and an acetylated chain end. The carboxylic chain end likely arises from the hydrolysis of the cyclic lactam chain end from the potassium hydroxide in the used in depolymerization.

Proposed Mechanism for CPL and Cyclic Dimer formation from PA6

Using the kinetic, molecular weight, and chain-end studies results, we concluded that the photothermal ring closing depolymerization of PA6 likely proceeds primarily *via* a mid-chain deprotonation followed by chain unzipping, supported by prior literature.[4] Possible mechanisms for CPL and cyclic dimer formation from PA6 can be seen below.

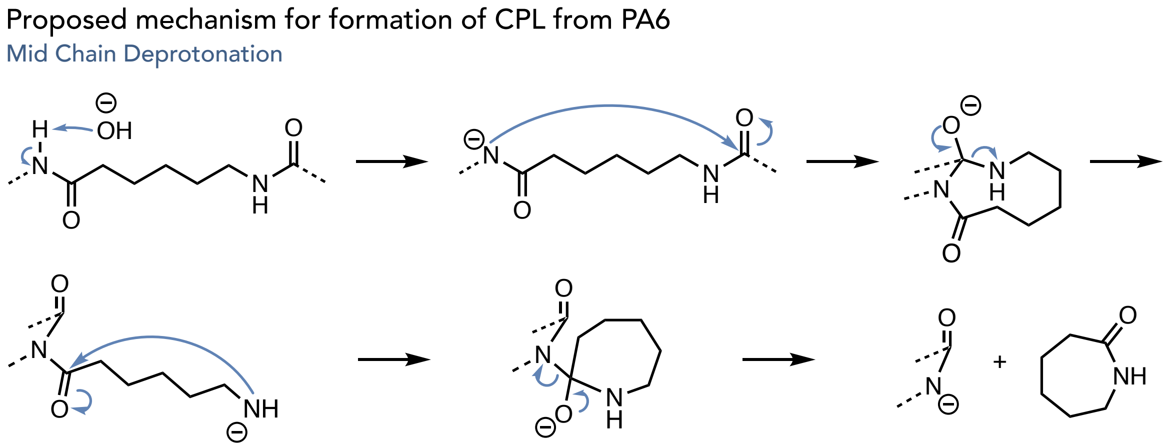

Figure S35. Possible Mechanism for CPL formation from PA6.

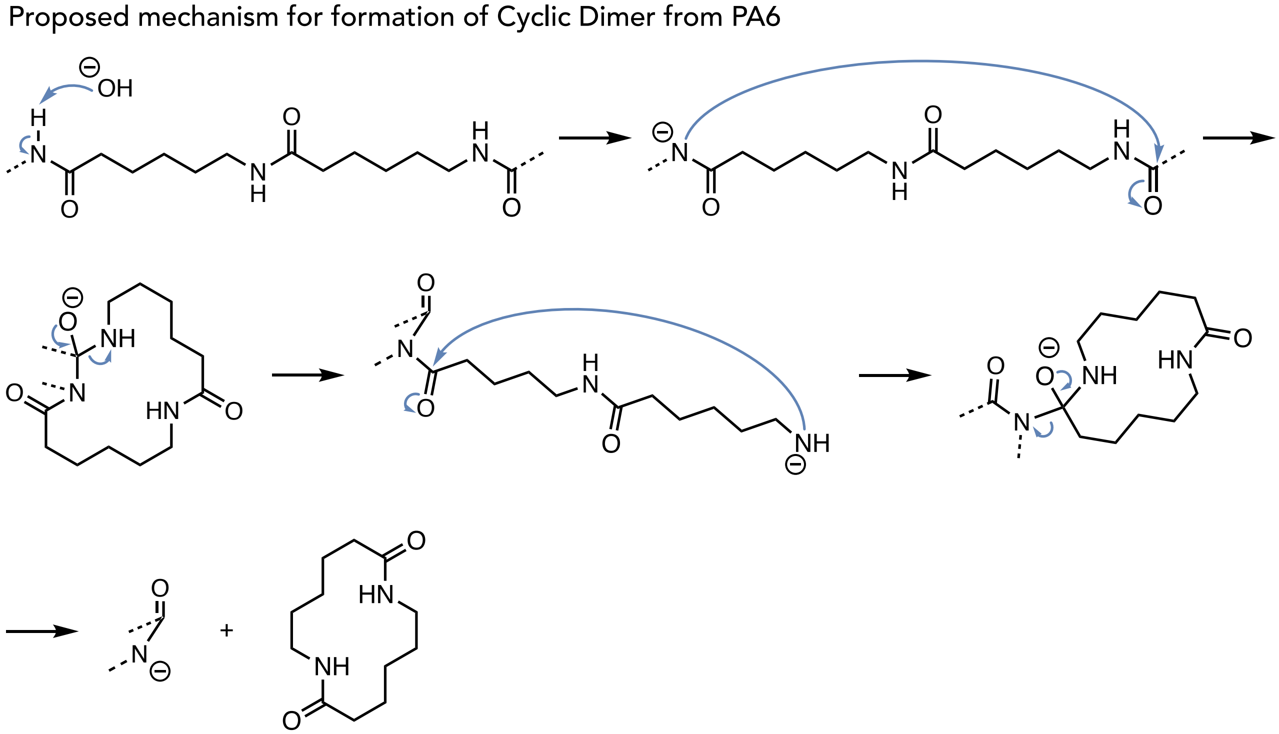

Figure S36. Possible Mechanism for Cyclic Dimer formation from PA6.

PA6 Photothermal Depolymerization Using Focused Sunlight as a Light Source

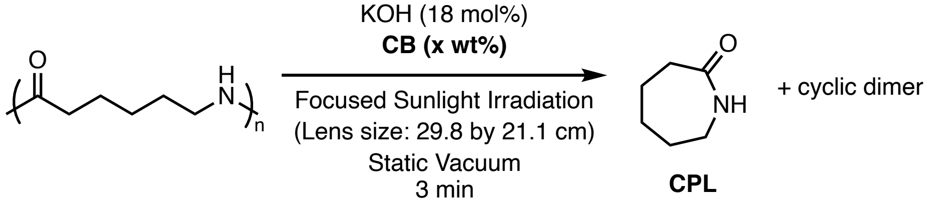

The procedure was slightly modified from the general PA6 ring-closing depolymerization procedure, where focused sunlight was used instead of a white LED light. On a sunny day, a plastic Fresnel lens (29.8 cm x 21.1 cm) was angled and clamped, such that the focal point was approximately the same area as the 1-dram reaction vial (177 mm^2). The focused sunlight light intensity per unit area was determined to be 28.1 W/cm^2 (see section entitled "Photothermal Reaction Setup" for calculation). The focused sunlight depolymerization procedure and setup was adapted from Oh et al., 2024.[5] The reaction workup was the same as the general PA6 photothermal depolymerization procedure. The depolymerization results are summarized below.

Table S16. Results of focused sunlight as an irradiation source for PA6 photothermal ring-closing depolymerization.

Entry	CB Loading (wt%)	CPL Yield$_{NMR}$(%)	Dimer Yield$_{NMR}$ (%)
1	2.5	60.0	1.3
2	0	0	0

Each entry is an average of 3 trials.

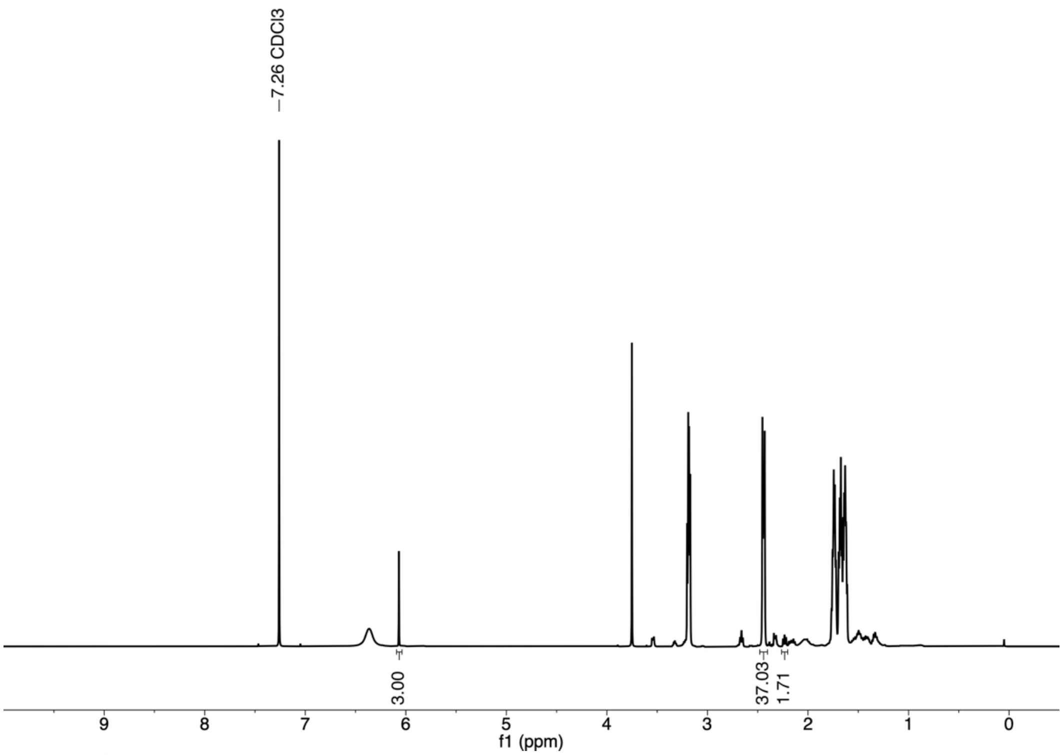

Figure S37. ^{1}H NMR of PA6 after photothermal ring-closing depolymerization using focused sunlight as a light source and 2.5 wt% CB.

PA6 Photothermal Depolymerization under Dynamic Vacuum

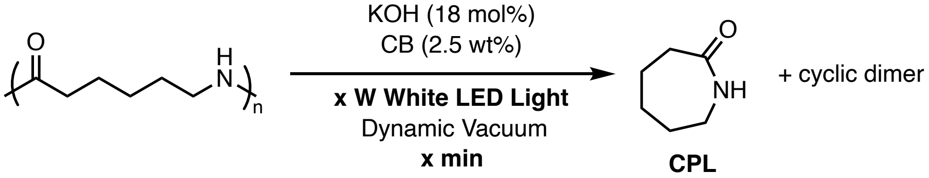

In a 1-dram glass vial, PA6 (77.4 mg, 0.684 mmol), KOH (6.9 mg, 0.123 mmol, 0.18 equiv.), and CB (2.2 mg, 2.5 wt% of total reaction volume) were added and mixed well by vortexing. The vial was partially wrapped with aluminum foil, leaving about 0.1 mm of the bottom of the vial unwrapped. The vial was then equipped with a vacuum adapter and connected to a Schlenk line. The reaction apparatus was clamped such that the bottom of the vial was 0.2 mm above a 6000K white LED light (14 or 21 W), and the apparatus was evacuated and backfilled with nitrogen three times and left under dynamic vacuum after a fourth vacuum pull. The reaction was irradiated for 10 or 60 minutes. After the reaction, the light was turned off, and the entire apparatus was backfilled with nitrogen. The vial was disconnected from the apparatus and cooled in a dry ice-acetone bath for 1 minute and defrosted to room temperature. $CDCl_3$ (1 mL) and a stock solution of 1,3,5-trimethoxybenzene stock solution (0.2 mL, 0.12 M in $CDCl_3$) were added to the reaction vial. The vacuum adapter was rinsed with $CDCl_3$ (2 mL) a stock solution of 1,3,5-trimethoxybenzene stock solution (0.2 mL, 0.12 M in $CDCl_3$) was added. Aliquots of the reaction vial and the vacuum adapter were taken for ^{1}H NMR analysis (100 µL of the dissolved reaction mixture was diluted with 0.35 mL $CDCl_3$).

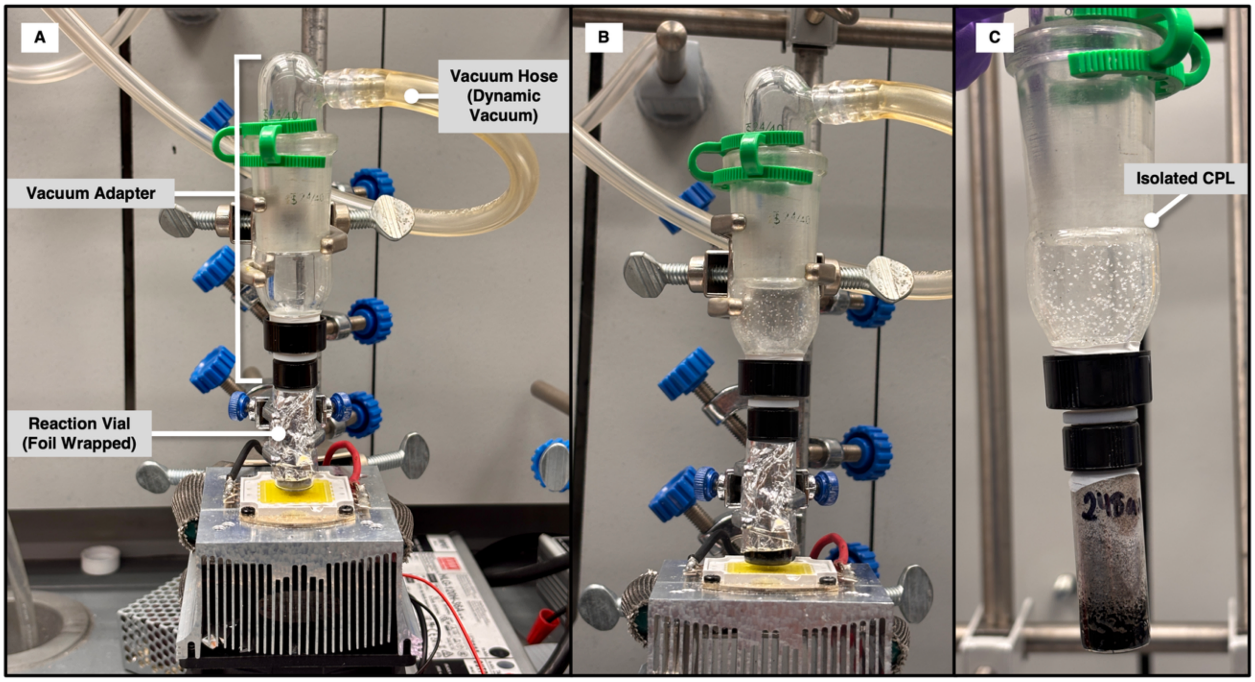

Figure S38. A) Dynamic vacuum reaction setup. B) Photo after PA6 photothermal depolymerization under dynamic vacuum. C) Close up image of unwrapped reaction vial and vacuum adapter after PA6 photothermal depolymerization under dynamic vacuum.

Table S17. Results of PA6 photothermal ring-closing depolymerization under dynamic vacuum.

Entry	Light Intensity (W)	Time (min)	Reaction Apparatus Part	CPL $Yield_{NMR}$(%)	Dimer $Yield_{NMR}$ (%)
1	14	60	Reaction Vial	0.1	2.0
			Vacuum Adapter	68.2	0.0
2	21	10	Reaction Vial	13.7	2.4
			Vacuum Adapter	50.9	0.4

Each entry is an average of 2 trials.

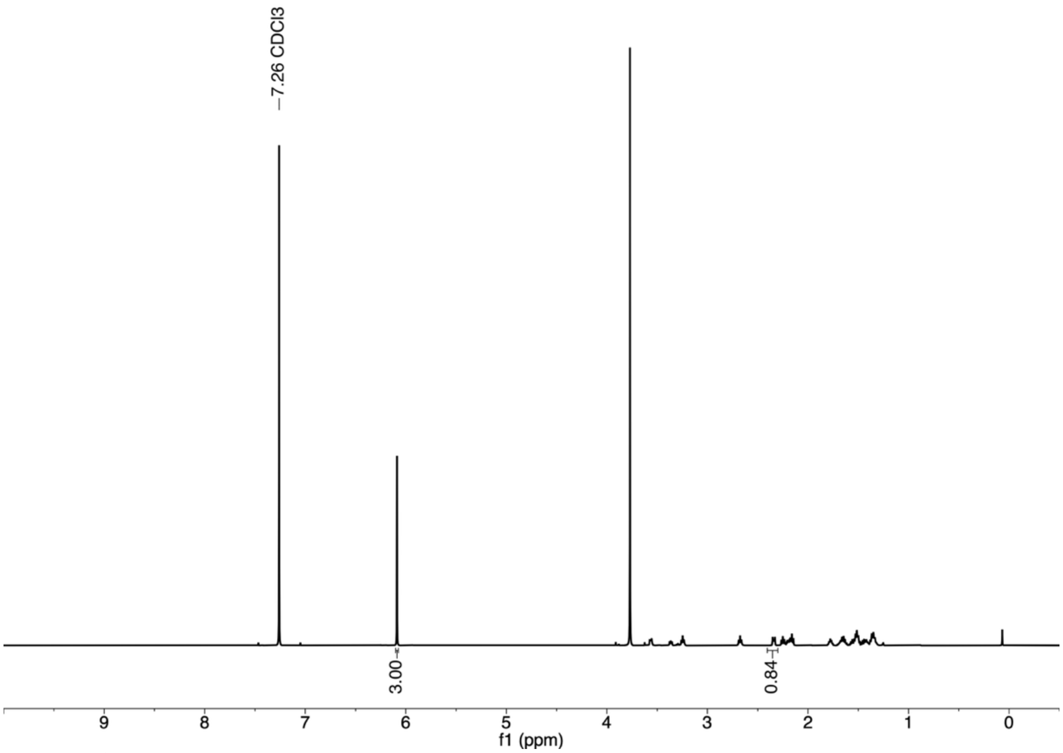

Figure S39. ^1H NMR of PA6 after photothermal ring-closing depolymerization under dynamic vacuum (reaction vial) using a 14 W White LED light.

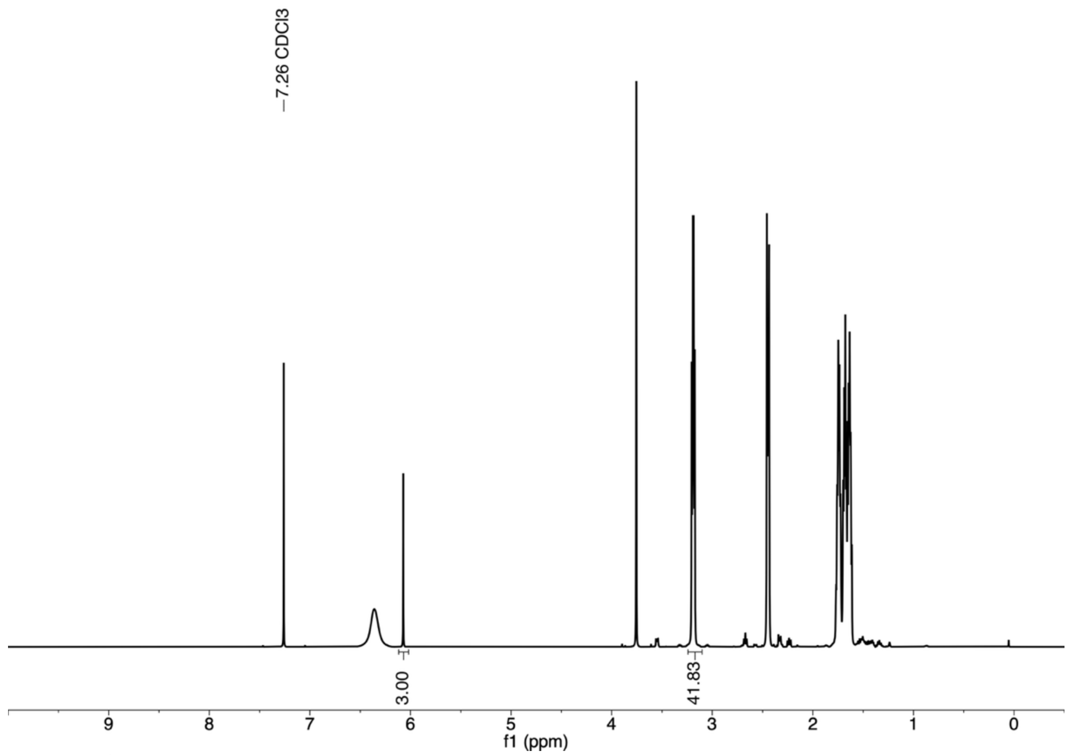

Figure S40. ^{1}H NMR of PA6 after photothermal ring-closing depolymerization under dynamic vacuum (vacuum adapter) using a 14 W White LED light.

Repolymerization of CPL after PA6 Photothermal Depolymerization

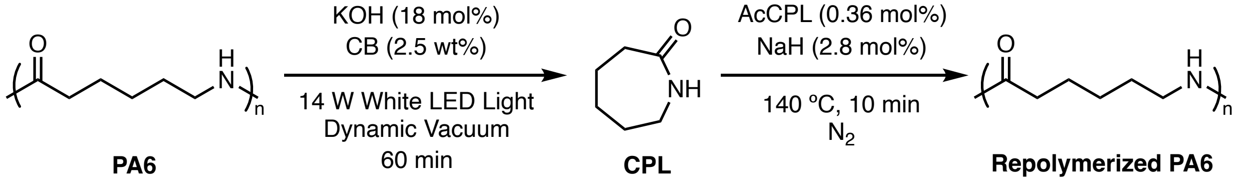

CPL collection was performed after a modified version of the PA6 ring-closing depolymerization using dynamic vacuum. After the reaction finished, the vacuum adapter was rinsed with MeOH (3 mL) and collected in a glass 1-dram vial. A small aliquot was taken for ^{1}H NMR analysis (100 µL of the solution was dried to remove solvent, the redissolved in 0.35 mL $CDCl_3$) shown below. The MeOH/CPL solution was passed through a cotton plug to remove any insoluble material. The solvent was evaporated from the reaction mixture, and the resulting solid dried under vacuum, weighed, and characterized using ^{1}H NMR analysis.

A total of 7 dynamic vacuum experiments were performed, each on a 100 mg PA6 scale (0.884 mmol PA6, 0.159 mmol KOH, 2.5 wt% CB), to collect CPL (519.5 mg, 4.95 mmol, 98.7% purity), with an average CPL yield of 74.2% across the 7 reactions. The collected CPL from each reaction was combined and purity was determined with ^{1}H NMR analysis. The collected CPL was subsequently transferred into a N_2 glovebox. A portion of the collected CPL (261 mg, 2.31 mmol) was polymerized using anionic-ring opening polymerization with the procedure previously mentioned (see section "Anionic Ring-Opening Polymerization for AcPA6 synthesis"). The resulting solid polymer product was isolated (203 mg, 1.79 mmol, 89.1% conversion, 94% purity) and characterized using ^{1}H NMR and ^{13}C NMR analysis in 3:1 TFE/$CDCl_3$. $DMSO_2$ (4.3 mg, 0.046 mmol) was added to the ^{1}H NMR sample to determine the purity of the synthesized polymer.

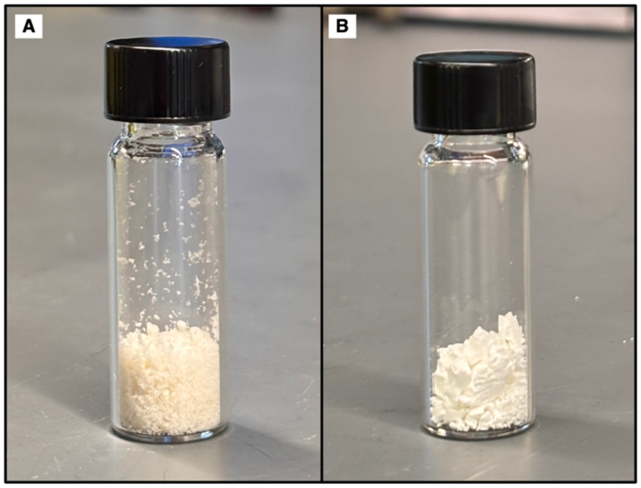

Figure S41. Collected CPL after depolymerization (A) and repolymerized PA6 from collected CPL (B).

Table S18. Results of repolymerization of CPL after PA6 photothermal depolymerization.

Entry	CPL (mg)	Conversion (%)	Isolated PA6 (mg)	Purity (%)
1	261	89.1	203.1	94.0

Conversion was measured using the ratio between monomer signal and PA6 signal in the crude ^{1}H NMR.

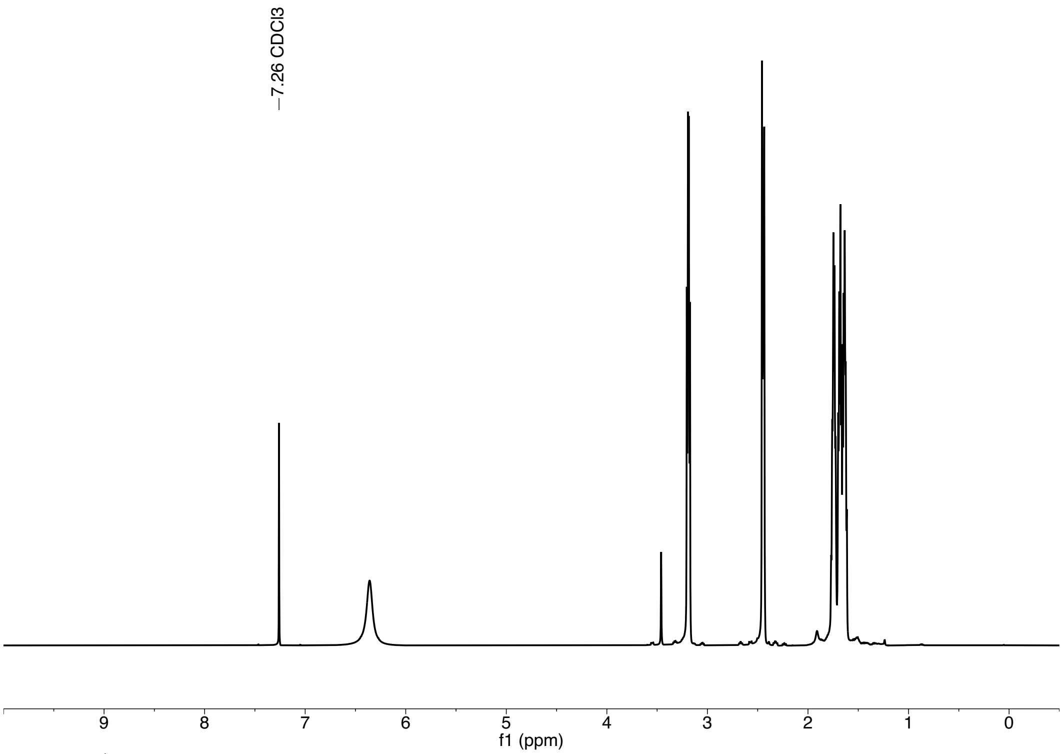

Figure S42. ^{1}H NMR of collected CPL from PA6 photothermal ring-closing depolymerization under dynamic vacuum experiments. A methanol impurity can be seen at 3.49 ppm.

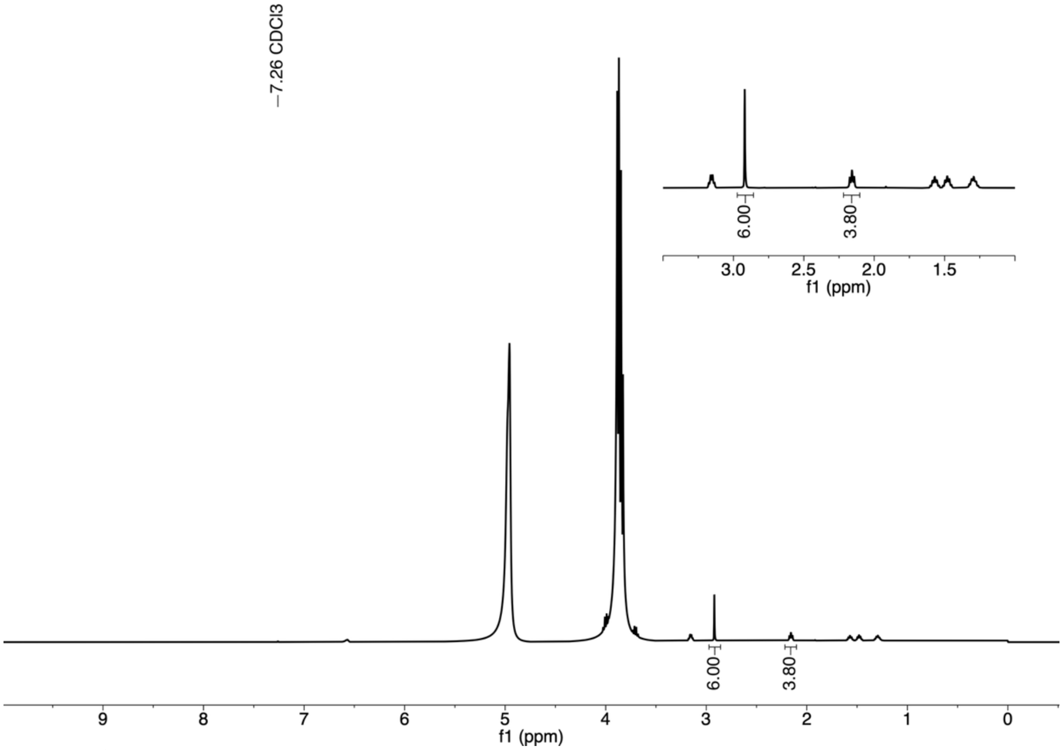

Figure S43. ^{1}H NMR of PA6 synthesized from collected CPL during dynamic vacuum depolymerization experiments in a 3:1 mixture of TFE/$CDCl_3$. Protonated TFE peaks appear at 3.8 ppm and 4.9 ppm, and $DMSO_2$ signal appears at 2.9 ppm.

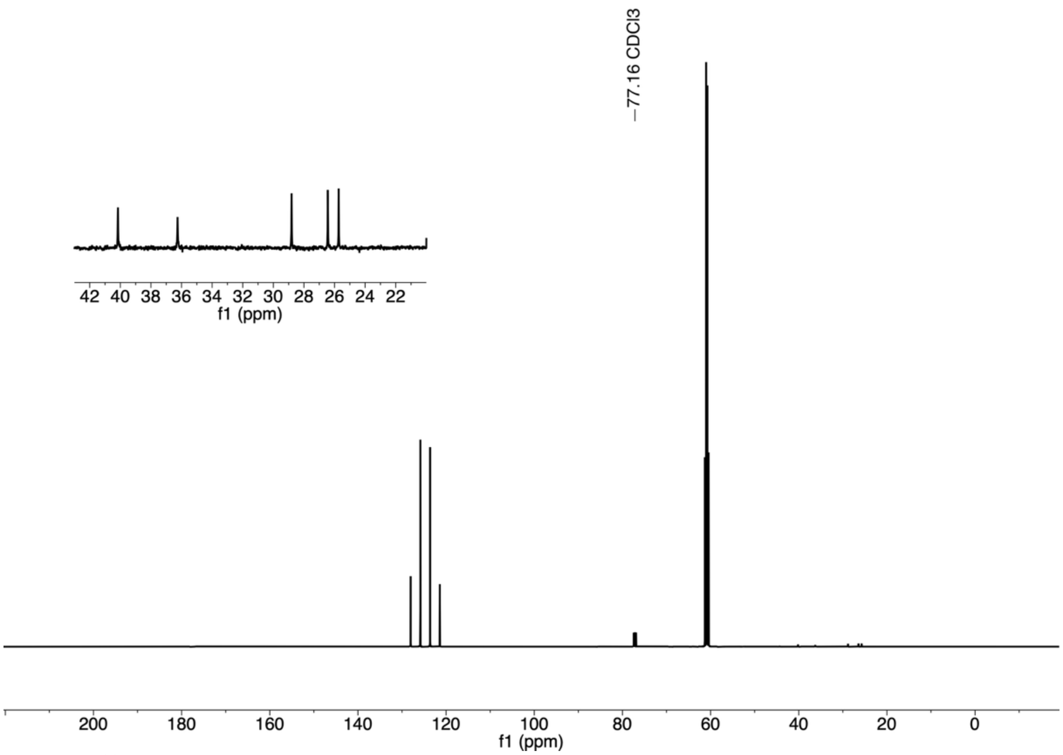

Figure S44. ^{13}C NMR of PA6 synthesized from collected CPL during dynamic vacuum depolymerization experiments. in a 3:1 mixture of TFE/$CDCl_3$. Protonated TFE peaks appear at 124 ppm and 61 ppm.

PA66 Photothermal Acidic Hydrolysis Optimization

General PA66 Photothermal Acidic Hydrolysis Conditions

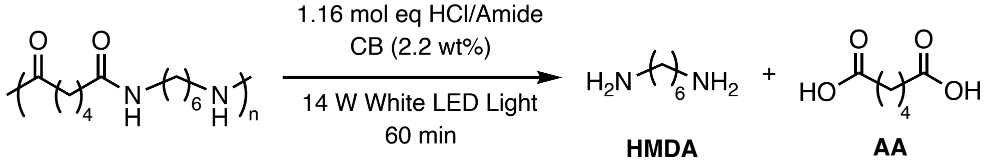

PA66 (100 mg, 0.442 mmol), CB (2.2 mg, 2.2 wt%), and 5.56 M HCl (184 µL, 1.02 mmol HCl) were added to a 1-dram vial and sealed with a pressure relief cap and gently vortexed to mix. The vial was subsequently placed 0.2 mm above a 6000K white LED light (14 W) and irradiated for 60 minutes. After the reaction, the vial left to cool to room temperature, then dried under high vacuum overnight to remove any residual water. After drying, 1,3,5-trimethoxybenzene (3 to 8 mg, 0.018 to 0.048 mmol) and DMSO-d_6 (0.75 mL) were added to the reaction vial and left to sit for at least 1 hour to completely dissolve. Aliquots were taken for ^{1}H NMR analysis (≤ 20 µL of the dissolved reaction mixture was diluted with 0.35 mL DMSO-d_6) shown below. All PA66 hydrolysis NMR data was collected on an NMR instrument with a room temperature probe to avoid peak broadening and uneven baselines caused by high ionic concentrations within the sample.

To isolate out the monomers from the reaction mixture, a modified workup procedure was used. After the reaction, without drying overnight, warm H_2O (30 mL, ~80 ºC) was added to the reaction vial, vortexed well to dissolve monomers, then passed through a cotton plug to remove CB and water-insoluble polymer and oligomers. The filtrate was cooled to 0 ºC to precipitate out the diacid monomer and collected *via* filtration (58.7 mg, 0.402 mmol, 90.9% AA isolated yield). The remaining filtrate was dried under vacuum to obtain the fully protonated HMDA monomer (49.8 mg, 0.421 mmol, 95.3% fully protonated HMDA isolated yield). Both monomers were analyzed using ^{1}H NMR analysis, with spectra shown below.

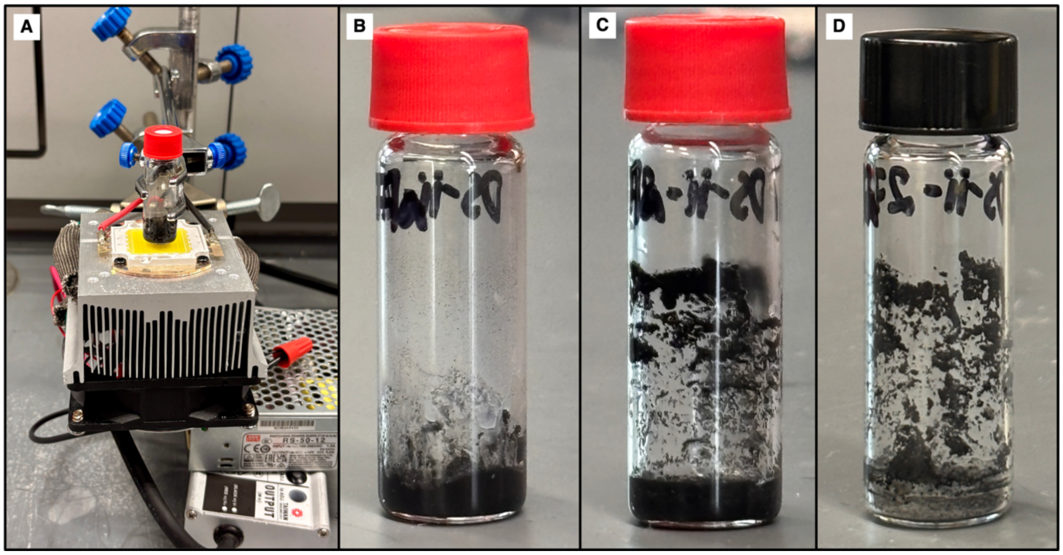

Figure S45. A) Reaction setup for PA66 photothermal acidic hydrolysis. B) PA66 photothermal hydrolysis reaction mixture before light irradiation. C) PA66 photothermal hydrolysis reaction mixture after light irradiation. D) PA66 photothermal hydrolysis reaction mixture after light irradiation and drying under vacuum overnight.

Table S19. Results of PA66 photothermal acidic hydrolysis using optimized conditions.

Entry	HMDA $Yield_{NMR}$(%)	AA $Yield_{NMR}$ (%)	HMDA $Yield_{isolated}$ (%)	AA $Yield_{isolated}$ (%)
1	97.3 (± 6.8)	96.2 (± 3.5)	95.3	90.9

NMR yields are averages of 3 trials. Error represented is the standard deviation between trials. Isolated yields were not replicated.

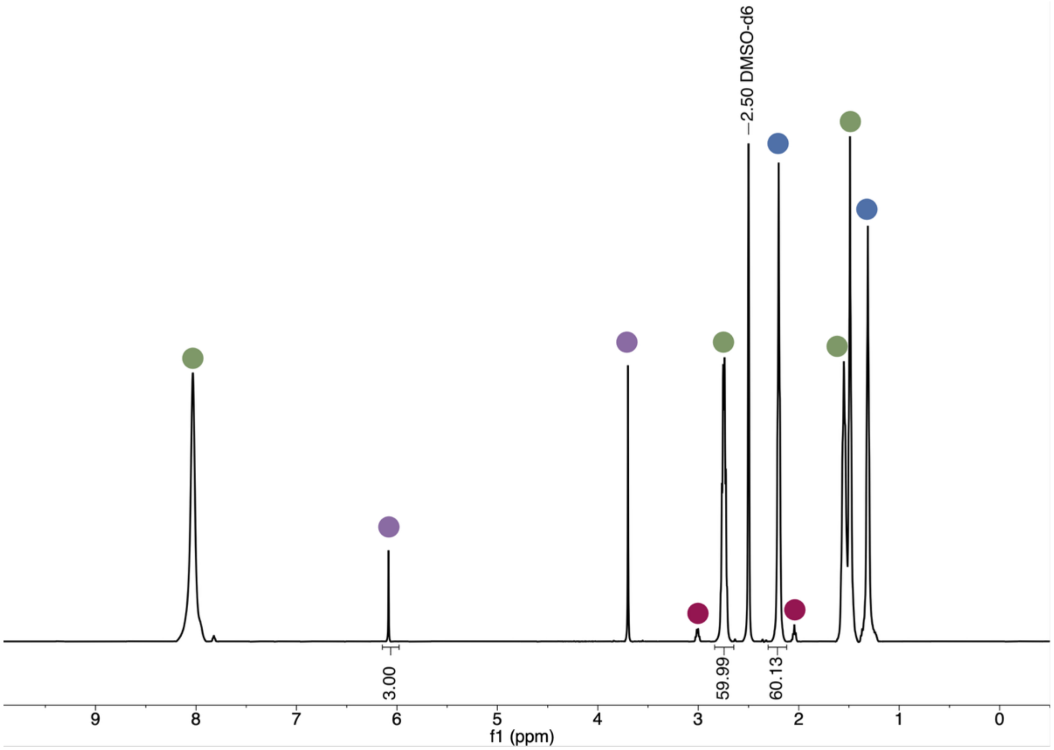

Figure S46. ^{1}H NMR of PA66 after photothermal acidic hydrolysis. TMB (purple circles), HMDA (green circles), AA (blue circles), and oligomer (maroon circles) signals are labeled.

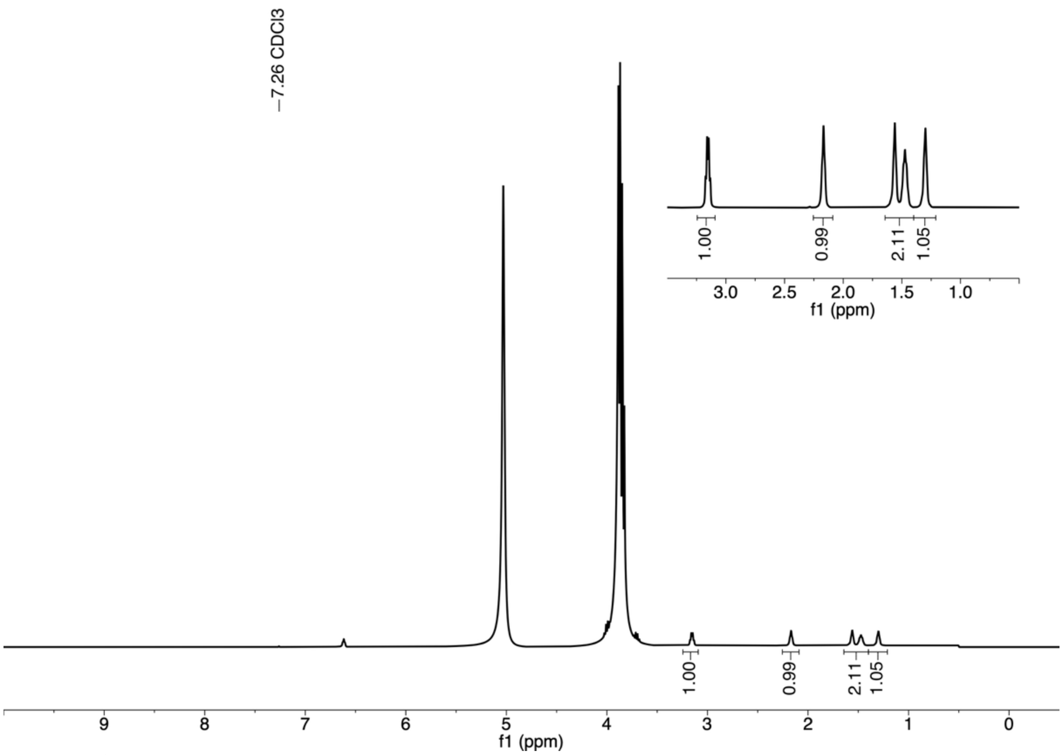

Figure S47. ^{1}H NMR of PA66 in 3:1 TFE/$CDCl_3$. The spectrum was baseline corrected between the 3.5 to 0.5 ppm region to ensure that protonated TFE signals appearing at 3.8 ppm and 4.9 ppm would not interfere with integrations.

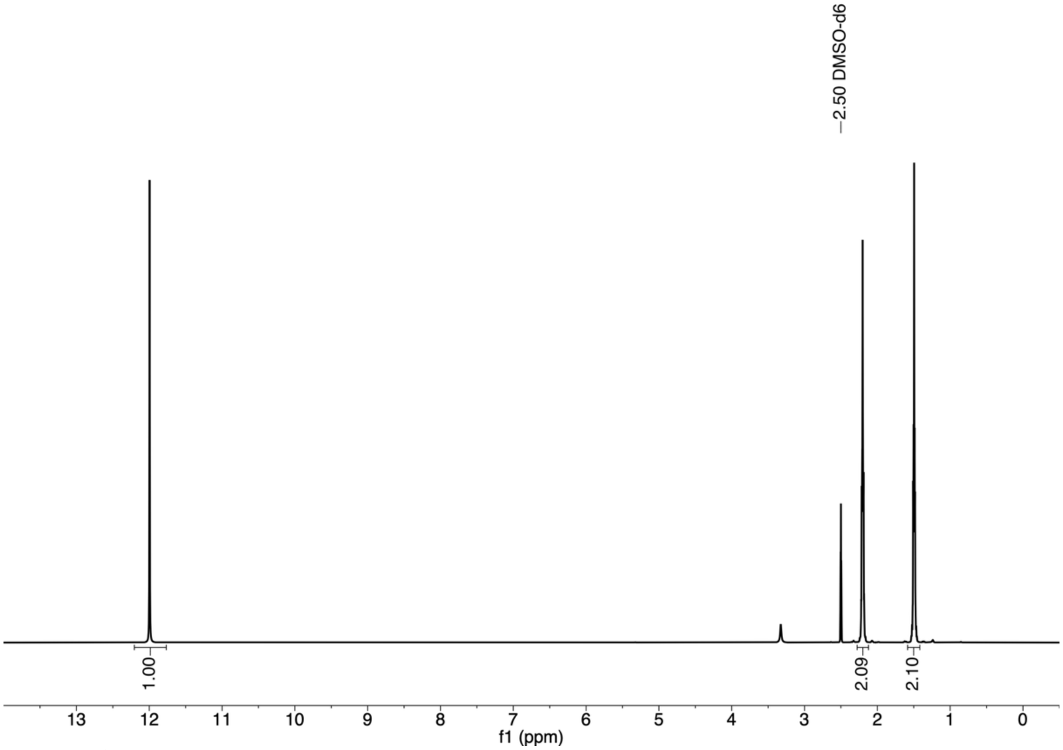

Figure S48. ^{1}H NMR of isolated AA from 100-mg scale photothermal acidic hydrolysis in DMSO-d_6.

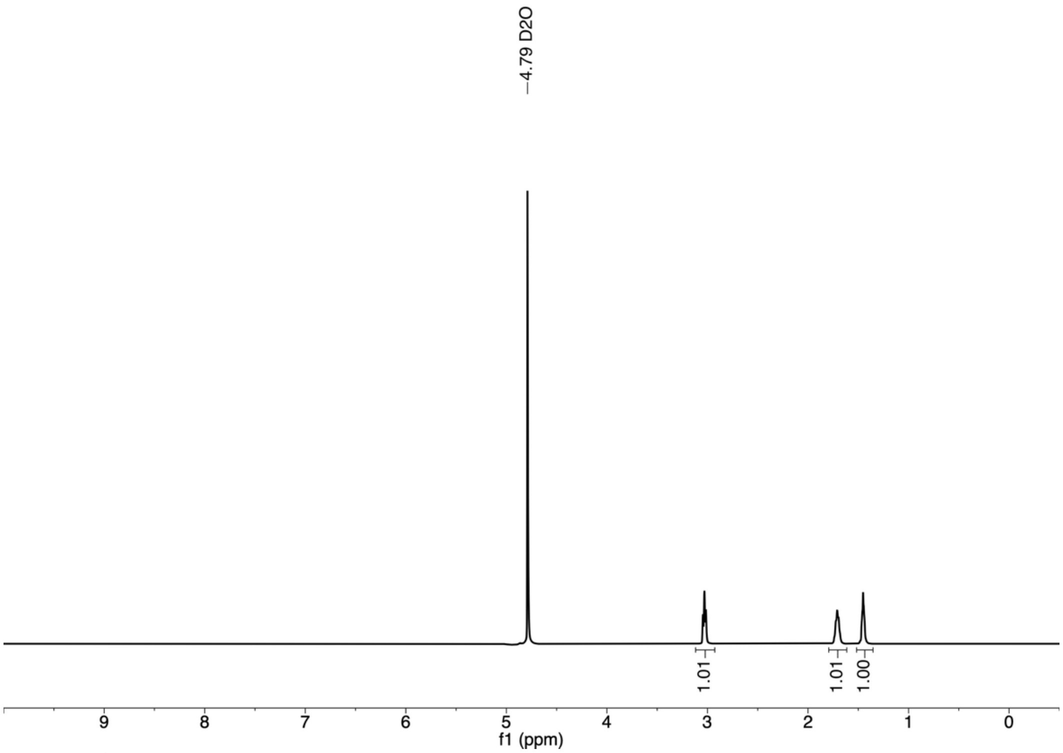

Figure S49. ^{1}H NMR of isolated fully protonated HMDA from 100-mg scale photothermal acidic hydrolysis in D_2O.

Control Experiments for PA66 Photothermal Acidic Hydrolysis

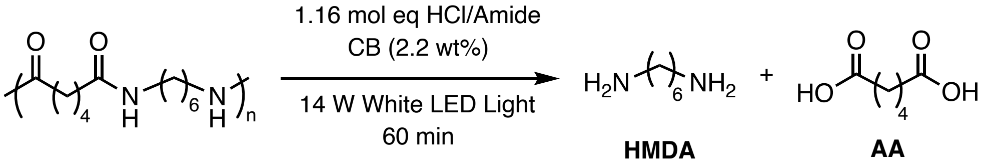

The procedure was slightly modified from the general PA66 acidic hydrolysis depolymerization procedure, and the deviations are listed below. ^{1}H NMR analysis was performed in DMSO-d_6 and leftover PA66 was not quantified. The reaction workup was the same as the general PA66 photothermal acidic hydrolysis procedure. The depolymerization results are summarized below.

Table S20. Results of PA66 photothermal acidic hydrolysis control experiments.

Entry	Variation	HMDA Yield$_{NMR}$(%)	AA Yield$_{NMR}$ (%)
1	Foil Wrapped	0	0
2	No light	0	0
3	2-dram vial instead of 1-dram vial	94.1	96.6
4	Water instead of aq. HCl	0	0

each entry is an average of 2 trials

Varied Carbon Black Loading for PA66 Photothermal Acidic Hydrolysis

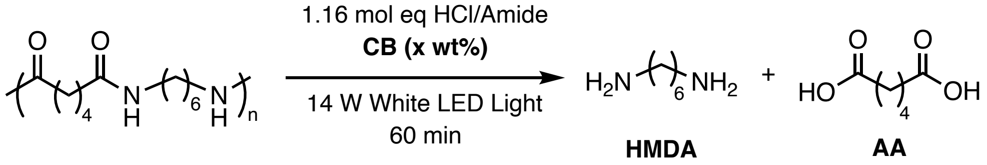

The procedure was slightly modified from the general PA66 acidic hydrolysis depolymerization procedure, where different loadings of CB were used. ^{1}H NMR analysis was performed in DMSO-d_6 and leftover PA66 was not quantified. The reaction workup was the same as the general PA66 photothermal acidic hydrolysis procedure. The depolymerization results are summarized below.

Table S21. Results of varied CB loading on PA66 photothermal acidic hydrolysis.

Entry	CB (wt%)	HMDA Yield$_{NMR}$(%)	AA Yield$_{NMR}$ (%)
1	0	0	0
2	0.4	86.9	86.3
3	0.9	90.8	91.6
4	2.2	97.3	96.2
5	2.7	93.2	92.1
6	3.5	92.6	98.3
7	4.4	93.8	96.0

each entry is an average of 2 trials

Varied Acid Loading (constant volume) for PA66 Photothermal Acidic Hydrolysis

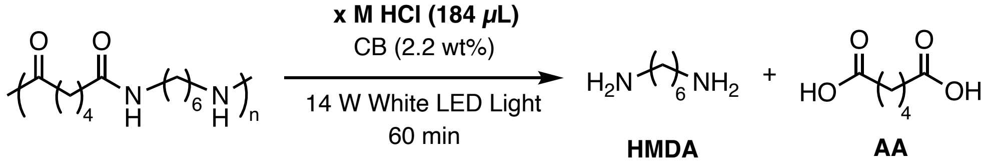

The procedure was slightly modified from the general PA66 acidic hydrolysis depolymerization procedure, where different concentrations of HCl to vary the HCl/Amide molar equivalents were used while keeping the total volume of liquid added the same (184 μL). ^{1}H NMR analysis was performed in DMSO-d_6 and leftover PA66 was not quantified. The reaction workup was the same as the general PA66 photothermal acidic hydrolysis procedure. The depolymerization results are summarized below.

Table S22. Results of varied acid loading (constant volume added) on PA66 photothermal acidic hydrolysis.

Entry	X	HCl/Amide	HMDA $Yield_{NMR}$(%)	AA $Yield_{NMR}$ (%)
1	7.2	1.50	95.3	97.7
2	6.0	1.25	99.1	90.5
3	5.56	1.16	97.3	96.2
4	4.8	1.00	79.2	96.1
5	3.6	0.75	42.3	48.7
6	2.4	0.50	23.9	24.2

each entry is an average of 2 trials

Varied Acid Loading (constant HCl/Amide molar ratio) for PA66 Photothermal Acidic Hydrolysis

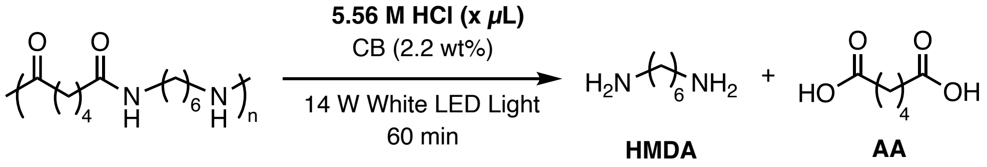

The procedure was slightly modified from the general PA66 acidic hydrolysis depolymerization procedure, where different volumes of 5.56 M HCl were used. ^{1}H NMR analysis was performed in DMSO-d_6 and leftover PA66 was not quantified. The reaction workup was the same as the general PA66 photothermal acidic hydrolysis procedure. The depolymerization results are summarized below.

Table S23. Results of varied acid loading (constant HCl/Amide ratio) on PA66 photothermal acidic hydrolysis.

Entry	X	HCl/Amide	HMDA Yield$_{NMR}$(%)	AA Yield$_{NMR}$ (%)
1	238	1.50	92.4	>99
2	199	1.25	95.1	>99
3	184	1.16	97.3	96.2
4	147	1.00	72.3	77.6
5	120	0.75	6.2	5.5
6	79	0.50	6.2	4.2

each entry is an average of 2 trials

Thermal Controls for PA66 Acidic Hydrolysis

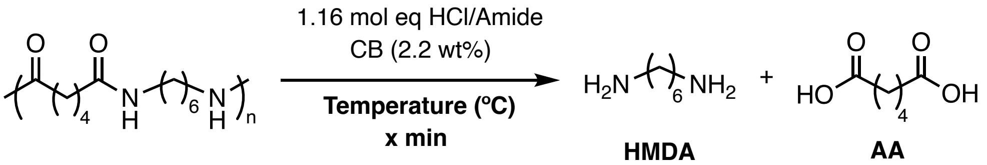

The procedure was modified from the general PA66 acidic hydrolysis depolymerization procedure, where the reaction proceeded via traditional thermal heating instead of photothermal conditions. A sand bath was preheated and equilibrated to 150 and 200 ºC, using a thermocouple to measure the temperature. Once the desired temperature stabilized, the reaction vial was inserted into the sand bath at a 2-cm depth for 60 or 120 minutes. ^{1}H NMR analysis was performed in DMSO-d_6 and leftover PA66 was not quantified. The reaction workup was the same as the general PA66 photothermal acidic hydrolysis procedure. The depolymerization results are summarized below. Attempts to run thermal control reactions at temperatures above 200 ºC resulted in the reaction vial breaking, likely due to a buildup of pressure from the aqueous acid.

Table S24. Results of thermal controls on PA66 acidic hydrolysis.

Entry	Temperature (ºC)	Time (min)	HMDA $Yield_{NMR}$(%)	AA $Yield_{NMR}$ (%)
1	150	60	67.7	77.1
2	150	120	72.1	73.6
3	200	60	66.5	72.1
4	200	120	96.2	98.3

each entry is an average of 2 trials

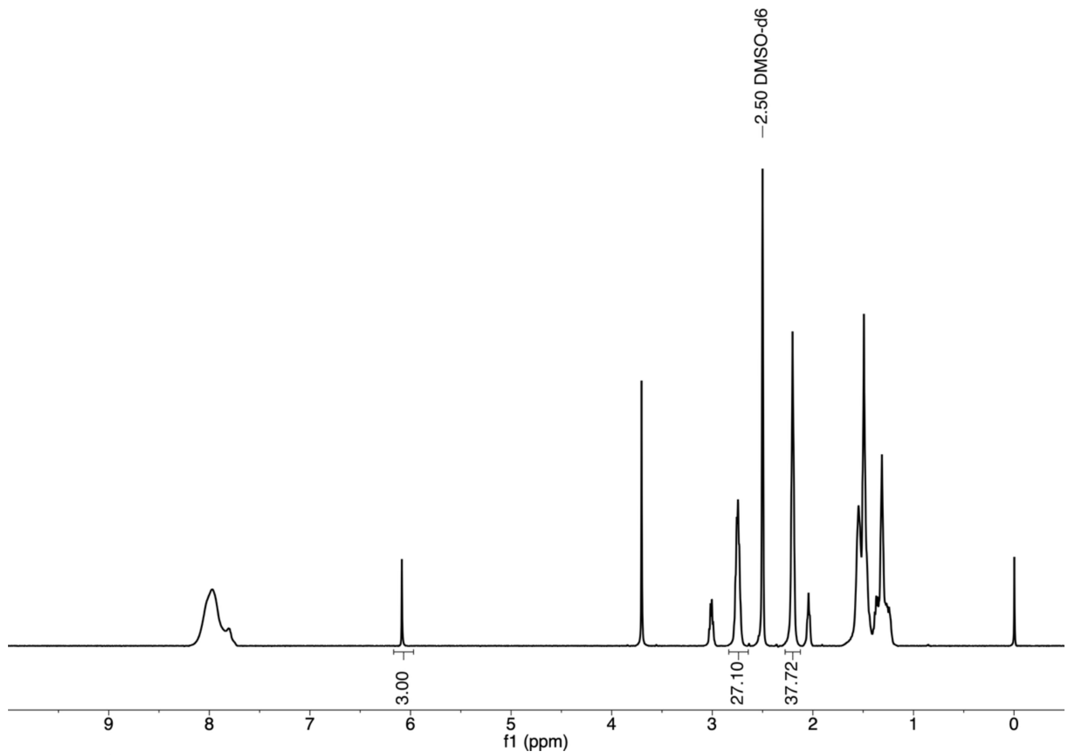

Figure S50. ^{1}H NMR of PA66 after thermal acidic hydrolysis at 200 ºC for 60 minutes.

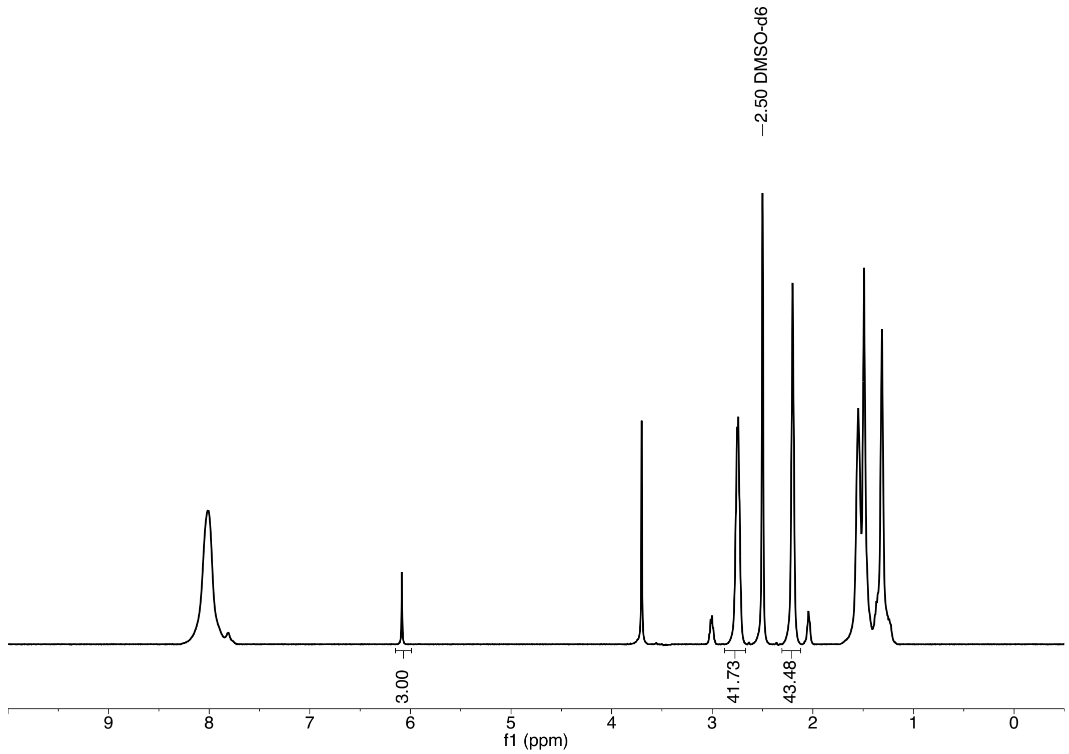

Figure S51. ^{1}H NMR of PA66 after thermal acidic hydrolysis at 200 ºC for 120 minutes.

Light Intensity Screen for PA66 Photothermal Acidic Hydrolysis

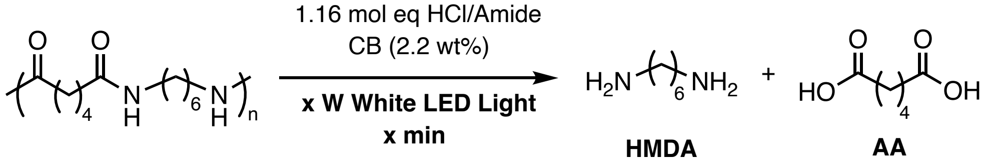

The procedure was slightly modified from the general PA66 acidic hydrolysis depolymerization procedure, where the light intensity was varied from 10 W to 21 W and the reaction time was either 10 or 60 minutes. ^{1}H NMR analysis was performed in DMSO-d_6 and leftover PA66 was not quantified. The reaction workup was the same as the general PA66 photothermal acidic hydrolysis procedure. The depolymerization results are summarized below.

Table S25. Results of light intensity screen for PA66 photothermal acidic hydrolysis.

Entry	Light Intensity (W)	Time (min)	HMDA Yield$_{NMR}$(%)	AA Yield$_{NMR}$ (%)
1	17	60	5.0	4.7
2	16	60	6.4	7.0
3	15	60	20.3	25.7
4	14	60	97.3	96.2
5	13	60	76.1	98.5
6	12	60	66.5	68.8
7	11	60	68.6	71.7
8	10	60	66.7	67.9
9	21	10	0.0	0.0

each entry is an average of 2 trials

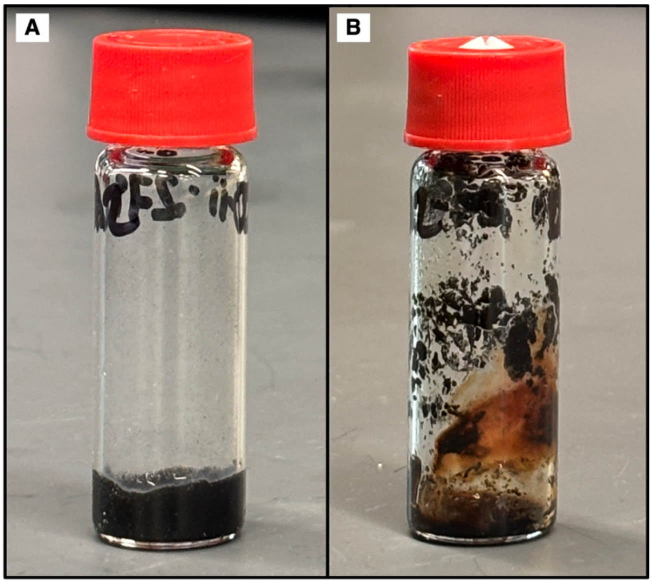

Figure S52. A) PA66 photothermal hydrolysis reaction vial before 21 W white light irradiation. B) PA66 photothermal hydrolysis reaction vial immediately after 10 minutes of 21 W white light irradiation.

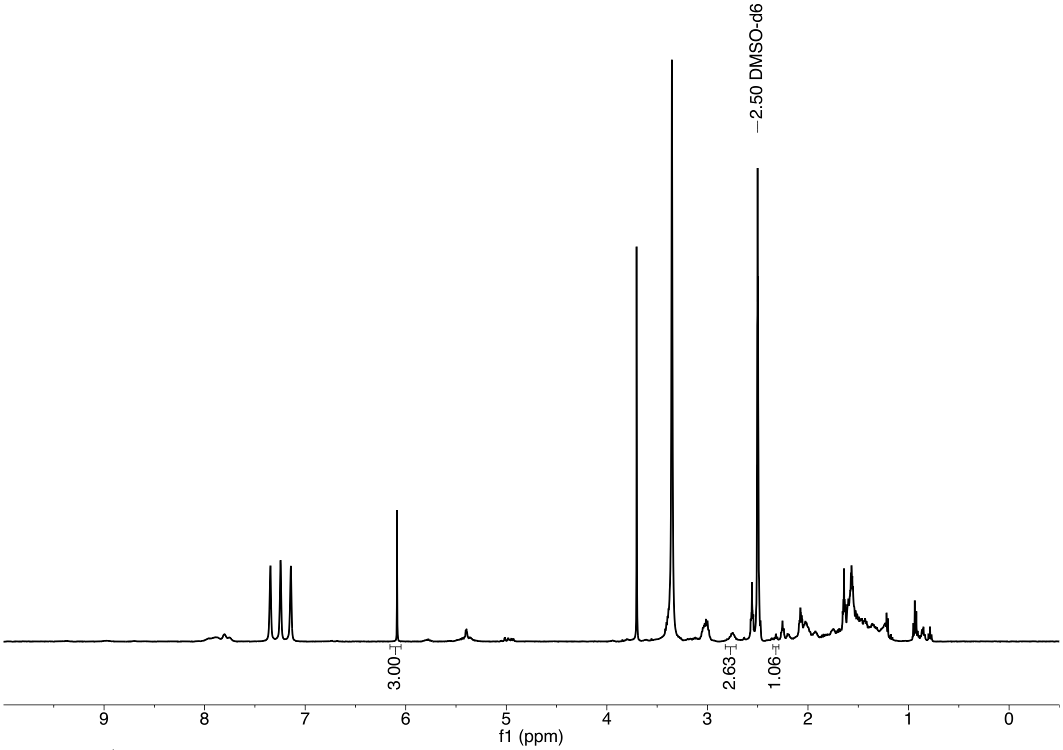

Figure S53. ^1H NMR of PA66 after photothermal acidic hydrolysis with 10 minutes of 21 W white light irradiation.

The use of higher light intensities (15 W and above) proved to be detrimental to reactivity, owing to the higher PTA surface temperatures, produced when irradiating with higher intensity light, leading to polymer degradation. At elevated temperatures, PA66 decomposes into a variety of degradation products, none of which were our desired target monomers AA and HMDA.[6] Thus,

we wanted to avoid using higher light intensities to limit the temperature near the PTA surface and hinder polymer degradation and charring into unwanted products.

Additional characterization was performed following PA66 photothermal hydrolysis using 10 minutes of 21 W light irradiation. After the reaction had ended, the solid residue inside the vial was rinsed with DI H_2O (3 x 1 mL) and acetone (3 x 1 mL), then dried under vacuum overnight to remove any moisture. The dried solid was ground with a mortar and pestle prior to FTIR characterization.

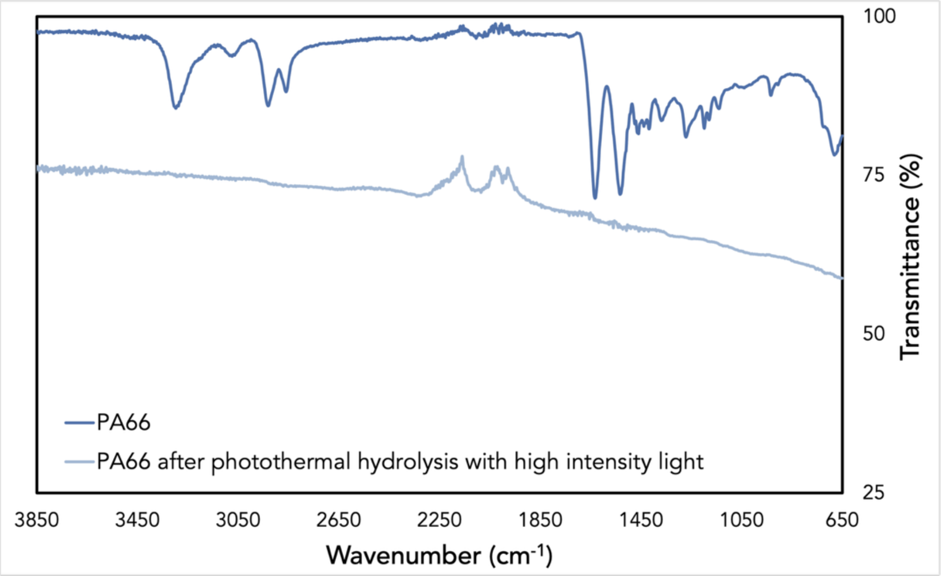

Figure S54. FTIR spectra of PA66 before (dark blue) and after (light blue) photothermal acidic hydrolysis with 10 minutes of 21 W white light irradiation.

Acid Screen for PA66 Photothermal Acidic Hydrolysis

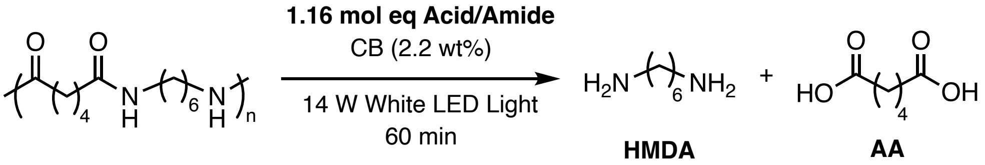

The procedure was slightly modified from the general PA66 acidic hydrolysis depolymerization procedure, where H_2SO_4, TsOH monohydrate, acetic acid, and H_3PO_4 were used instead of HCl (184 µL of a 5.56M acid solution was added to each reaction, for 1.16 mol equiv. HCl/Amide). ^{1}H NMR analysis was performed in DMSO-d_6 and leftover PA66 was not quantified. The reaction workup was the same as the general PA66 photothermal acidic hydrolysis procedure. The depolymerization results are summarized below.

Table S26. Results of acid screen for PA66 photothermal acidic hydrolysis.

Entry	Acid	HMDA Yield$_{NMR}$(%)	AA Yield$_{NMR}$ (%)
1	H_2SO_4	84.2	88.1
2	TsOH · H_2O	0.0	0.7
3	CH_3COOH	0.0	0.0
4	H_3PO_4	27.4	33.8

each entry is an average of 2 trials

PA66 Photothermal Acidic Hydrolysis Kinetics

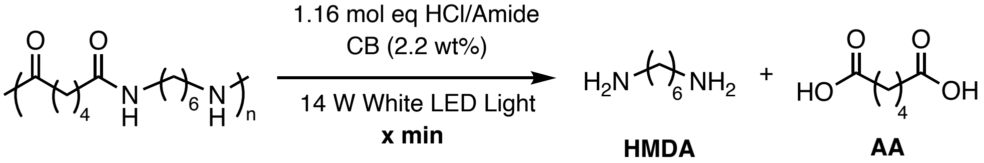

The procedure was the same as the general PA66 acidic hydrolysis depolymerization procedure, except that the depolymerization time was varied from 0 minutes to 120 minutes. ^{1}H NMR analysis was performed in DMSO-d_6 and leftover PA66 was not quantified. The reaction workup was the same as the general PA66 photothermal acidic hydrolysis procedure. The depolymerization results are summarized below.

Table S27. Results of PA66 photothermal acidic hydrolysis kinetics.

Entry	Time (min)	HMDA Yield$_{NMR}$(%)	AA Yield$_{NMR}$ (%)
1	0	0	0
2	1	0	0
3	2.5	8.8	9.0
4	5	44.6	46.7
5	7.5	70.6	72.9
6	10	77.6	78.5
7	15	85.7	84.4
8	30	81.7	81.3
9	60	97.3	96.2
10	120	94.5	>99

each entry is an average of 2 trials

Large Scale PA66 Photothermal Acidic Hydrolysis

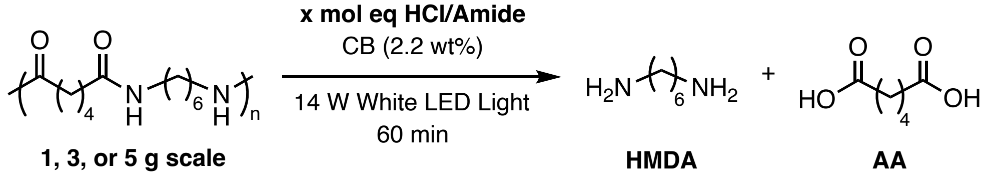

For the 1-gram scale, the procedure was slightly modified from the general PA66 acidic hydrolysis depolymerization procedure, where a 20 mL scintillation vial was used instead of a 1-dram vial and 1.25 molar equivalents HCl per amide were used (using 5.56 M HCl). Following the reaction, the reaction mixture was dried for 24 hours under vacuum instead of overnight to account for the additional liquid added.

For the 3- and 5-gram scales, the procedure was slightly modified from the general PA66 acidic hydrolysis depolymerization procedure. Instead of a 1-dram vial and 1.16 molar equivalents of HCl, a 40 mL scintillation vial and 1.5 molar equivalents HCl per amide were used (using 7.2 M HCl). The reaction setup was also modified and used two 14 W lights (one on the bottom of the vial, and one on the side of the vial) instead of just one light at the bottom of the vial. Following the reaction, the reaction mixture was dried for 48 hours under vacuum instead of overnight to account for the additional liquid added. The depolymerization results are summarized below.

To isolate out the monomers from the reaction mixture, a modified workup procedure was used. After the large-scale reaction (5000 mg PA66, 22.09 mmol), the reaction vial was cooled to room temperature then placed in an ice bath for 5 minutes. A NaOH solution (1.0 M, 18 mL) was added to the reaction vial in 6 mL increments, swirling the mixture in-between. Then, the contents of the vial were transferred to a centrifuge tube along with DI H_2O (20 mL), vortexed to mix, centrifuged, and the supernatant was collected in a separate flask. HCl (6.0 M, 3 mL) was added to the supernatant, which was then cooled to 0 ºC to precipitate out the diacid monomer and collected *via* filtration (2942.0 mg, 20.13 mmol, 91.9% AA isolated yield, > 99% purity). The remaining filtrate was basified using NaOH (1.0 M, 25 mL) then concentrated on a rotary evaporator and dried under vacuum to obtain the diamine monomer (2327.6 mg, 20.03 mmol, 90.7% HMDA isolated yield, > 99% purity). Both monomers were characterized using ^{1}H and ^{13}C NMR analysis. Purity was also determined using ^{1}H NMR against a TMB or maleic acid internal standard for AA and HMDA, respectively. The NMR spectra and photos of the monomers are shown below.

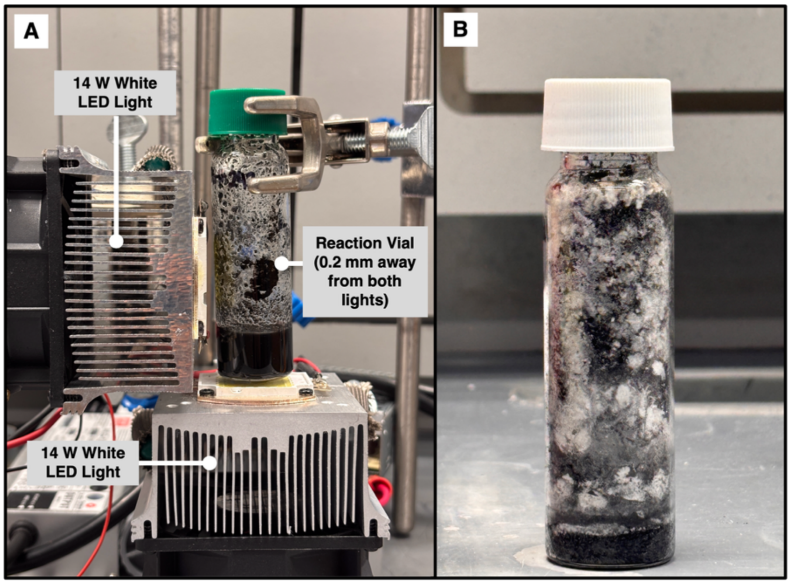

Figure S55. A) Reaction setup for the 3- and 5-gram large scale PA66 photothermal acidic hydrolysis. B) Reaction vial from the 5-gram large scale PA66 photothermal acidic hydrolysis after drying overnight.

Table S28. Results of large scale PA66 photothermal acidic hydrolysis.

Entry	# of Lights	Scale (g)	HCl/Amide	HMDA $Yield_{NMR}$(%)	AA $Yield_{NMR}$ (%)
1	1	1	1.25	>99	>99
2	2	3	1.5	94.3	>99
3	2	5	1.5	96.4	97.2

each entry is an average of 2 trials

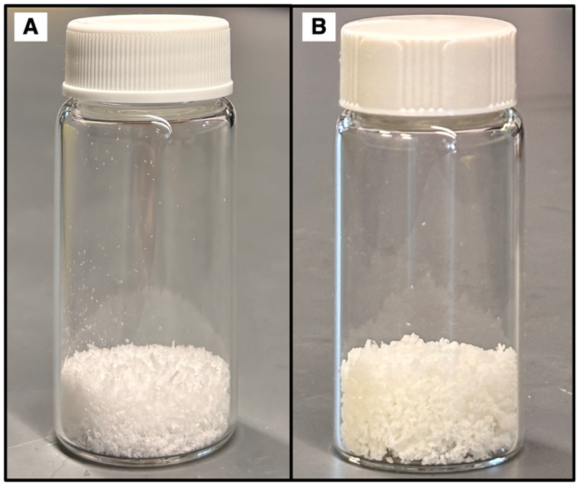

Figure S56. A) Isolated AA from 5-gram large scale PA66 photothermal acidic hydrolysis. B) Isolated HMDA from 5-gram large scale PA66 photothermal acidic hydrolysis.

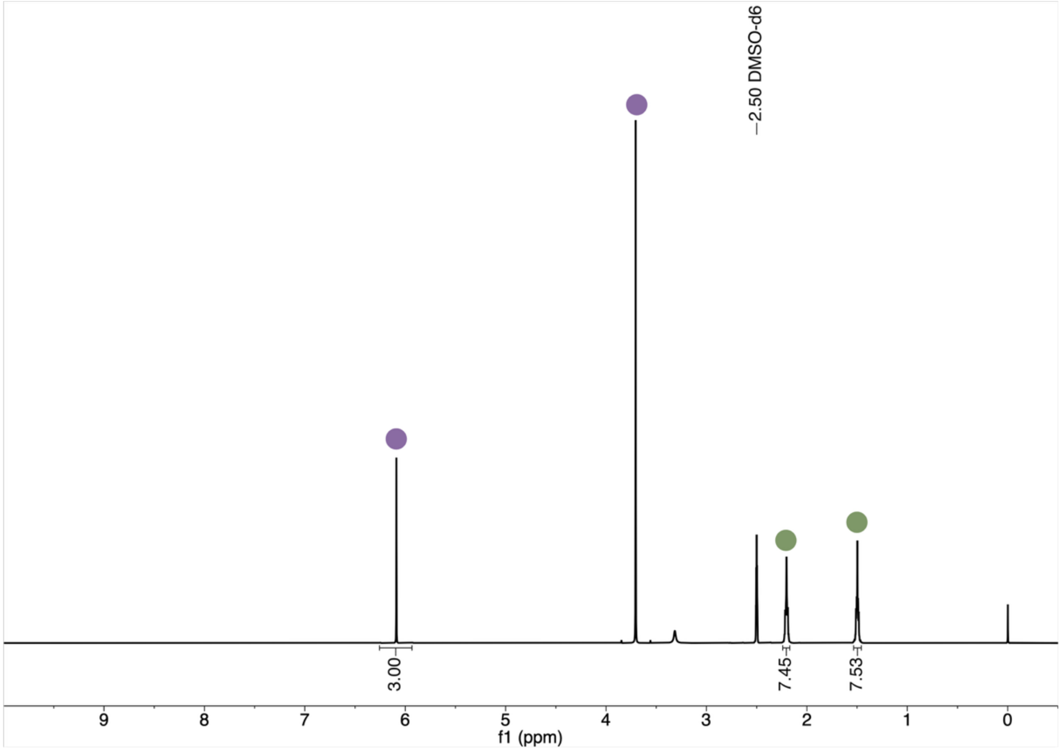

Figure S57. ^{1}H NMR of isolated AA from 5-gram large scale PA66 photothermal acidic hydrolysis. TMB (purple circles) and AA (green circles) signals are labeled. A water impurity can be seen at 3.3 ppm, likely from the NMR solvent.

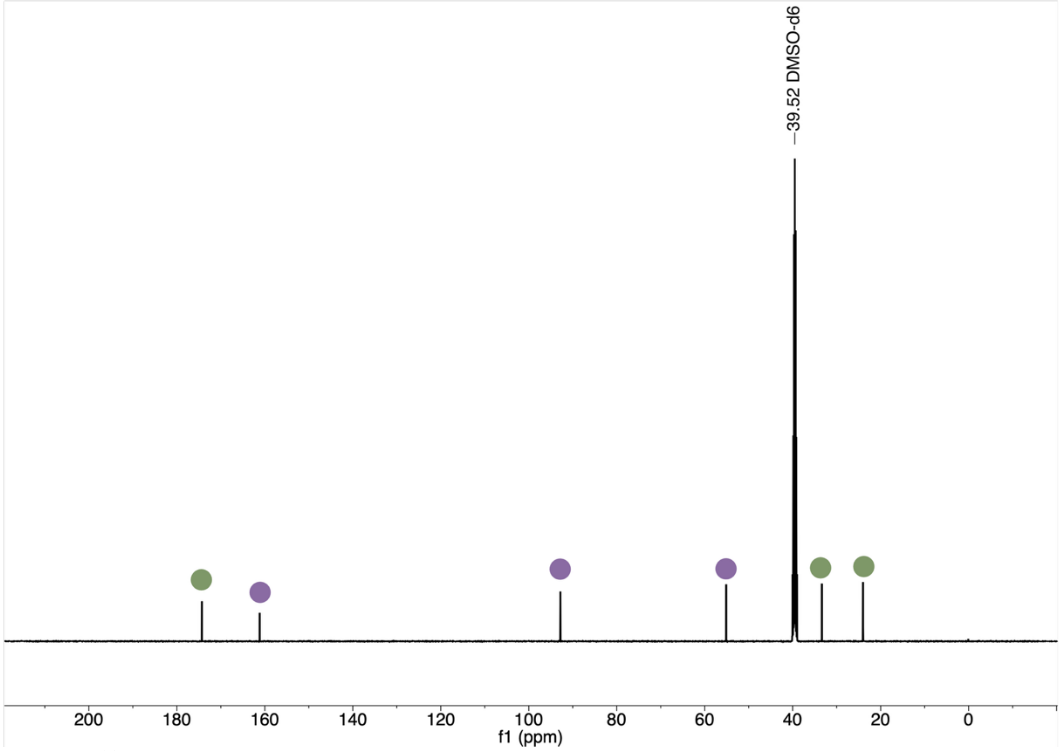

Figure S58. ^{13}C NMR of isolated AA from 5-gram large scale PA66 photothermal acidic hydrolysis. TMB (purple circles) and AA (green circles) signals are labeled.

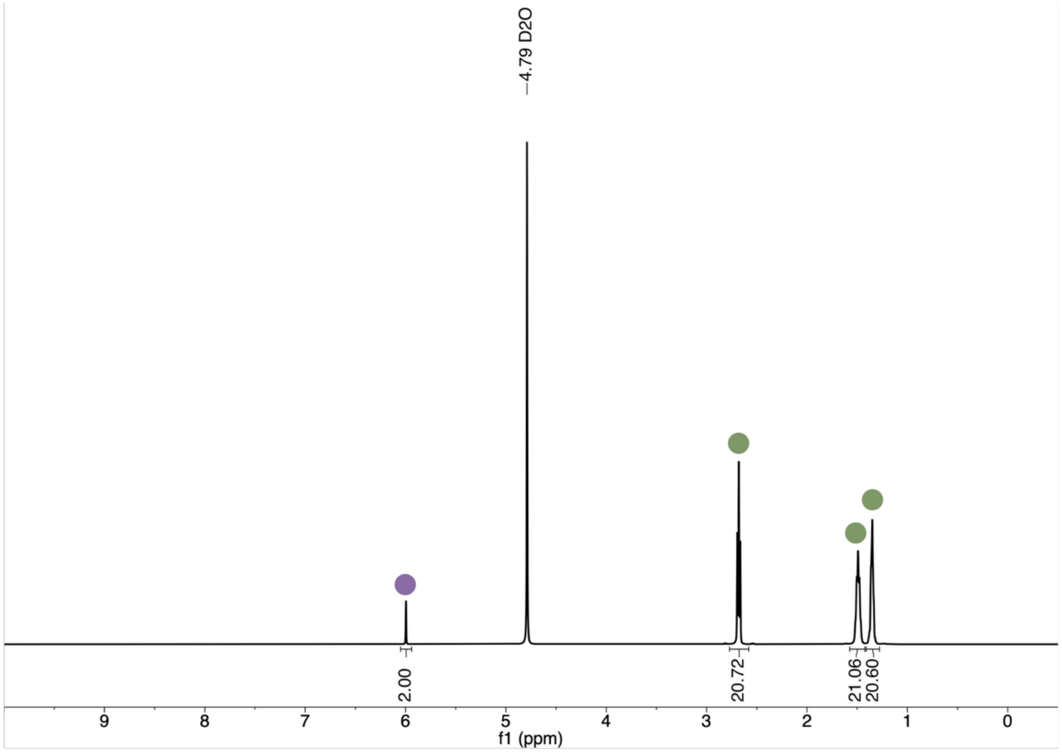

Figure S59. ^{1}H NMR of isolated HMDA from 5-gram large scale PA66 photothermal acidic hydrolysis. Maleic acid (purple circles) and HMDA (green circles) signals are labeled.

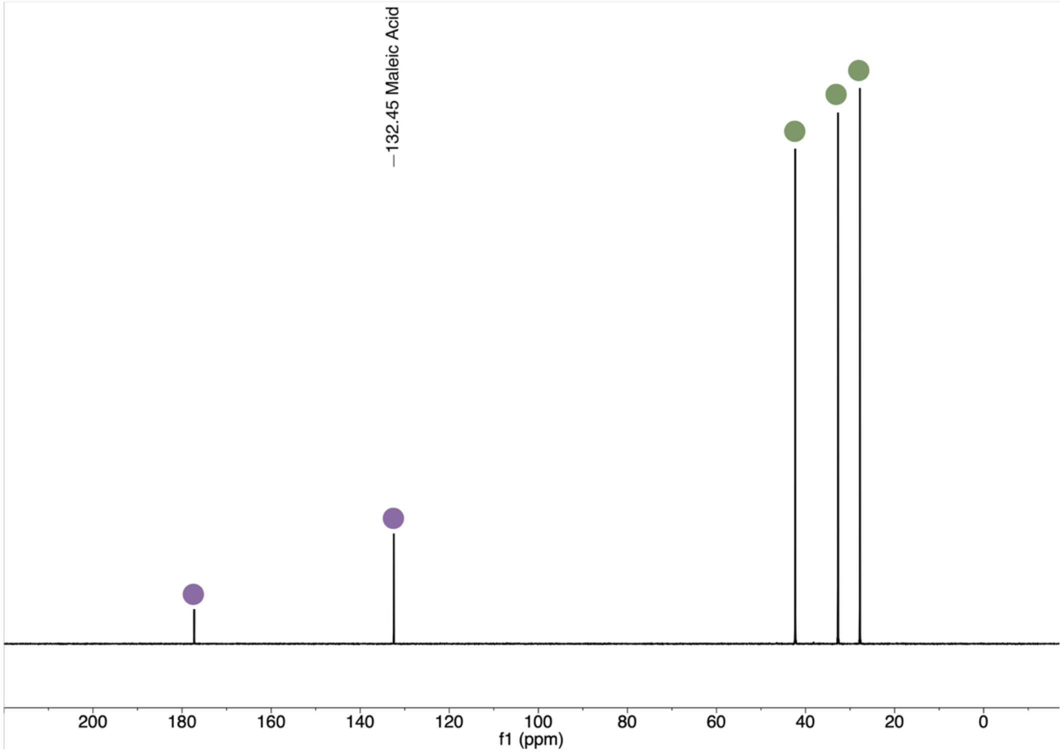

Figure S60. ^{13}C NMR of isolated HMDA from 5-gram large scale PA66 photothermal acidic hydrolysis. Maleic acid (purple circles) and HMDA (green circles) signals are labeled.

PA Photothermal Acidic Hydrolysis Scope

PA11 Photothermal Acidic Hydrolysis

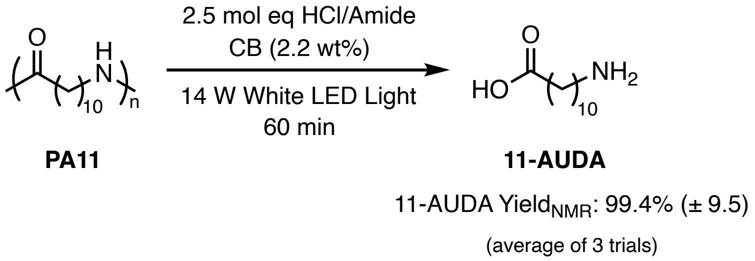

Prior to depolymerization, purchased PA11 pellets were melt pressed into a 1-mm thick film at 195 ºC, then cut into small 1 mm pieces. PA11 (100 mg, 0.546 mmol), CB (2.2 mg, 2.2 wt%), and 5.56 M HCl (245 µL, 1.36 mmol HCl, 2.5 mol equiv. per amide) were added to a 1-dram vial and sealed with a pressure relief cap and gently vortexed to mix. The vial was subsequently placed 0.2 mm above a 6000K white LED light (14 W) and irradiated for 60 minutes. After the reaction, the vial left to cool to room temperature, then dried under high vacuum overnight to remove any residual water. After drying, TMB (3 to 8 mg, 0.018 to 0.048 mmol) and DMSO-d_6 (0.75 mL) were added to the reaction vial and left to sit for at least 1 hour to completely dissolve. Aliquots were taken for ^{1}H NMR analysis (≤ 20 µL of the dissolved reaction mixture was diluted with 0.35 mL DMSO-d_6) shown below. Residual PA11 was not quantified.

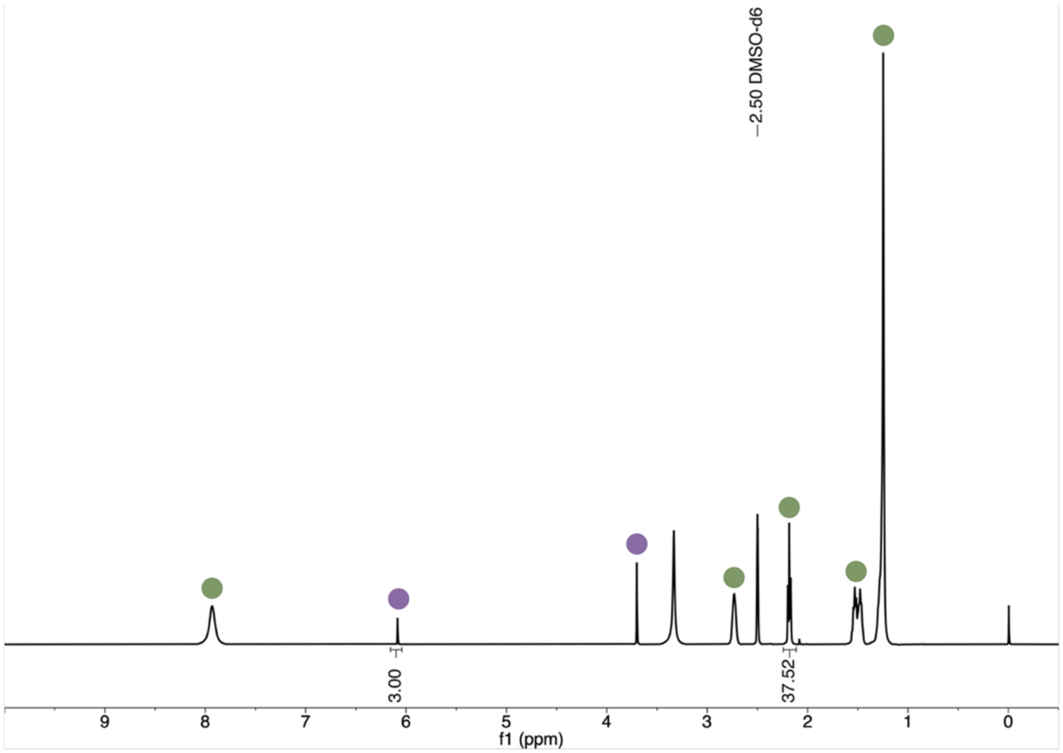

Figure S61. ^{1}H NMR of PA11 after photothermal acidic hydrolysis. TMB (purple circles) and 11-AUDA (green circles) signals are labeled. A water impurity can be seen at 3.33 ppm.

PA12 Photothermal Acidic Hydrolysis

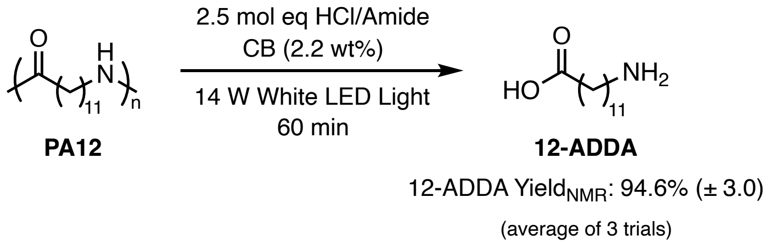

Prior to depolymerization, purchased PA12 pellets were melt pressed into a 1-mm thick film at 185 ºC using a Dabpress heat press (12-TON 4x7''), then cut into small 1 mm pieces. PA12 (100 mg, 0.507 mmol), CB (2.2 mg, 2.2 wt%), and 5.56 M HCl (227 µL, 1.26 mmol HCl, 2.5 mol equiv. per amide) were added to a 1-dram vial and sealed with a pressure relief cap and gently vortexed to mix. The vial was subsequently placed 0.2 mm above a 6000K white LED light (14 W) and irradiated for 60 minutes. After the reaction, the vial left to cool to room temperature, then dried under high vacuum overnight to remove any residual water. After drying, TMB (3 to 8 mg, 0.018 to 0.048 mmol) and DMSO-d_6 (0.75 mL) were added to the reaction vial and left to sit for at least 1 hour to completely dissolve. Aliquots were taken for ^{1}H NMR analysis (≤ 20 µL of the dissolved reaction mixture was diluted with 0.35 mL DMSO-d_6) shown below. Residual PA12 was not quantified.

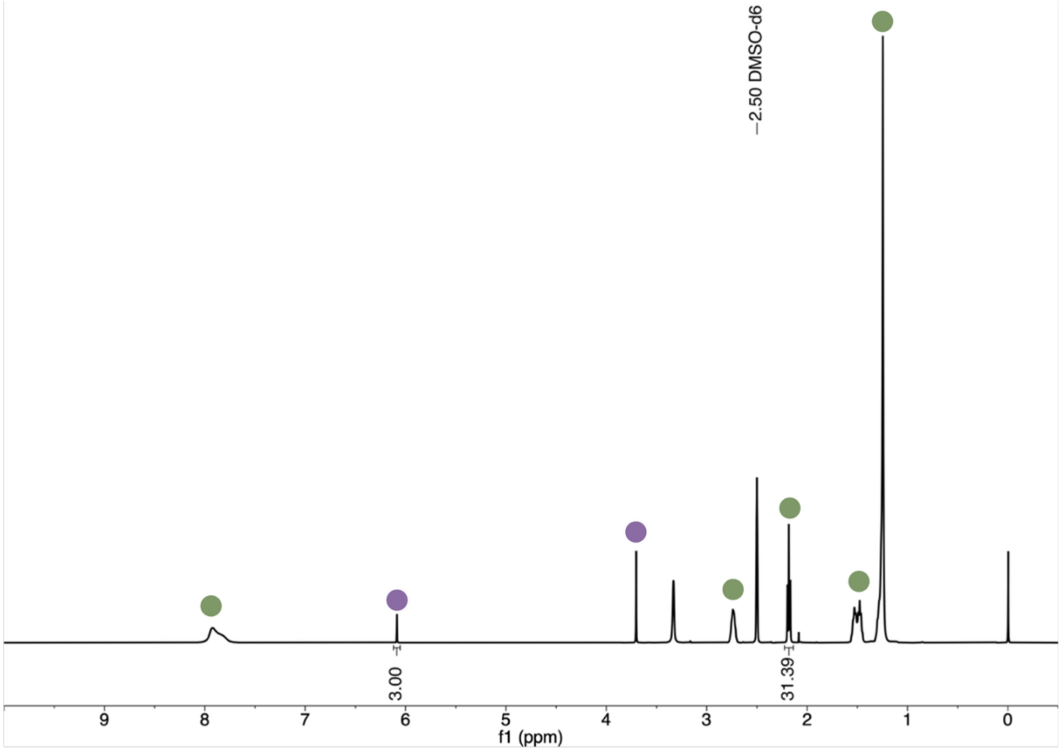

Figure S62. ^{1}H NMR of PA12 after photothermal acidic hydrolysis. TMB (purple circles) and 12-ADDA (green circles) signals are labeled. A water impurity can be seen at 3.33 ppm.

PA6 Photothermal Acidic Hydrolysis

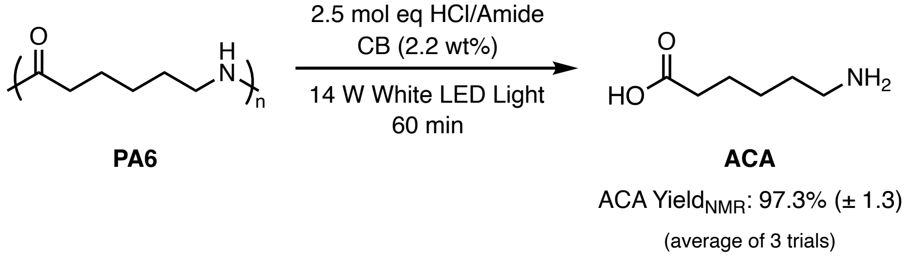

PA6 (100 mg, 0.884 mmol), CB (2.2 mg, 2.2 wt%), and 8.8 M HCl (251 µL, 2.21 mmol HCl, 2.5 mol equiv. per amide) were added to a 1-dram vial and sealed with a pressure relief cap and gently vortexed to mix. The vial was subsequently placed 0.2 mm above a 6000K white LED light (14 W) and irradiated for 60 minutes. After the reaction, the vial left to cool to room temperature, then dried under high vacuum overnight to remove any residual water. After drying, $DMSO_2$ (5-9 mg, 0.053-0.096 mmol) and D_2O (1 mL) were added to the reaction vial and left to sit for at least 1 hour to completely dissolve. Aliquots were taken for ^{1}H NMR analysis (≤ 20 µL of the dissolved reaction mixture was diluted with 0.35 mL D_2O) shown below. Residual PA6 was not quantified.

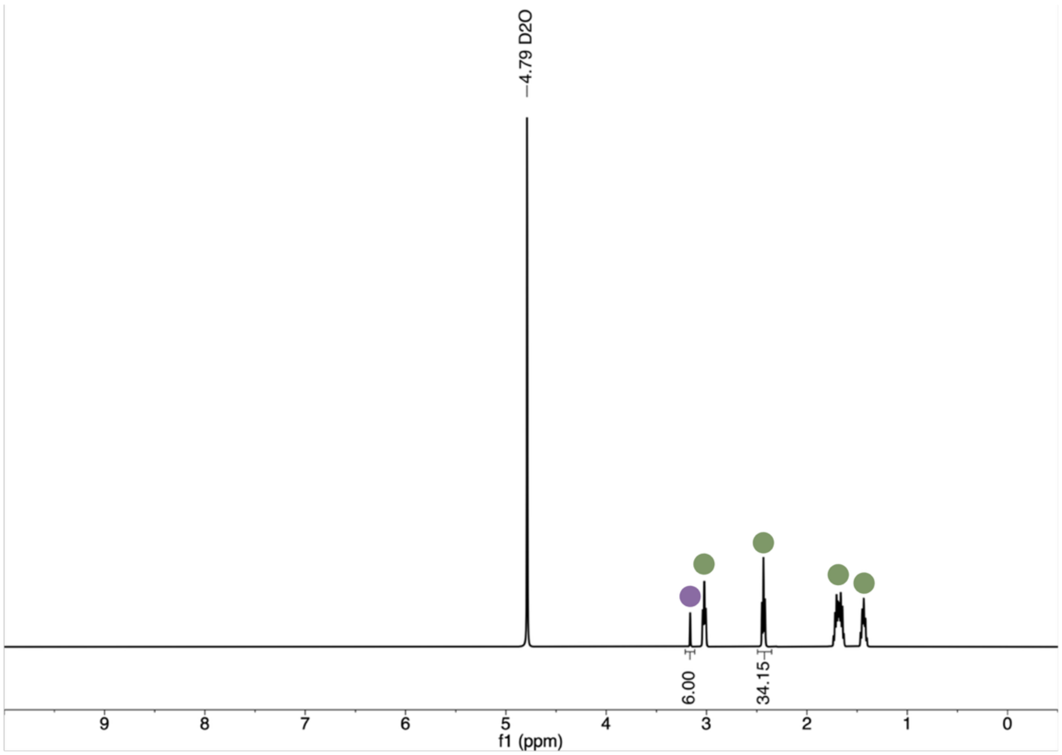

Figure S63. ^{1}H NMR of PA6 after photothermal acidic hydrolysis. $DMSO_2$ (purple circle) and ACA (green circles) signals are labeled.

PA610 Photothermal Acidic Hydrolysis

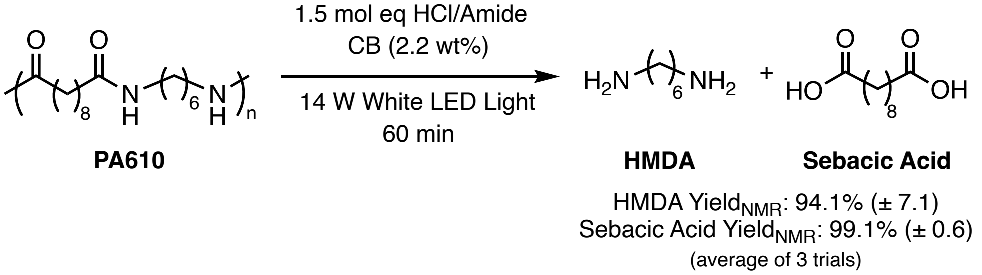

PA610 (100 mg, 0.354 mmol), CB (2.2 mg, 2.2 wt%), and 5.56 M HCl (191 µL, 1.06 mmol HCl, 1.5 mol equiv. per amide) were added to a 1-dram vial and sealed with a pressure relief cap and gently vortexed to mix. The vial was subsequently placed 0.2 mm above a 6000K white LED light (14 W) and irradiated for 60 minutes. After the reaction, the vial left to cool to room temperature, then dried under high vacuum overnight to remove any residual water. After drying, TMB (3 to 8 mg, 0.018 to 0.048 mmol) and DMSO-d$_6$ (0.75 mL) were added to the reaction vial and left to sit for at least 1 hour to completely dissolve. Aliquots were taken for ^{1}H NMR analysis (≤ 20 µL of the dissolved reaction mixture was diluted with 0.35 mL DMSO-d$_6$) shown below. Residual PA610 was not quantified.

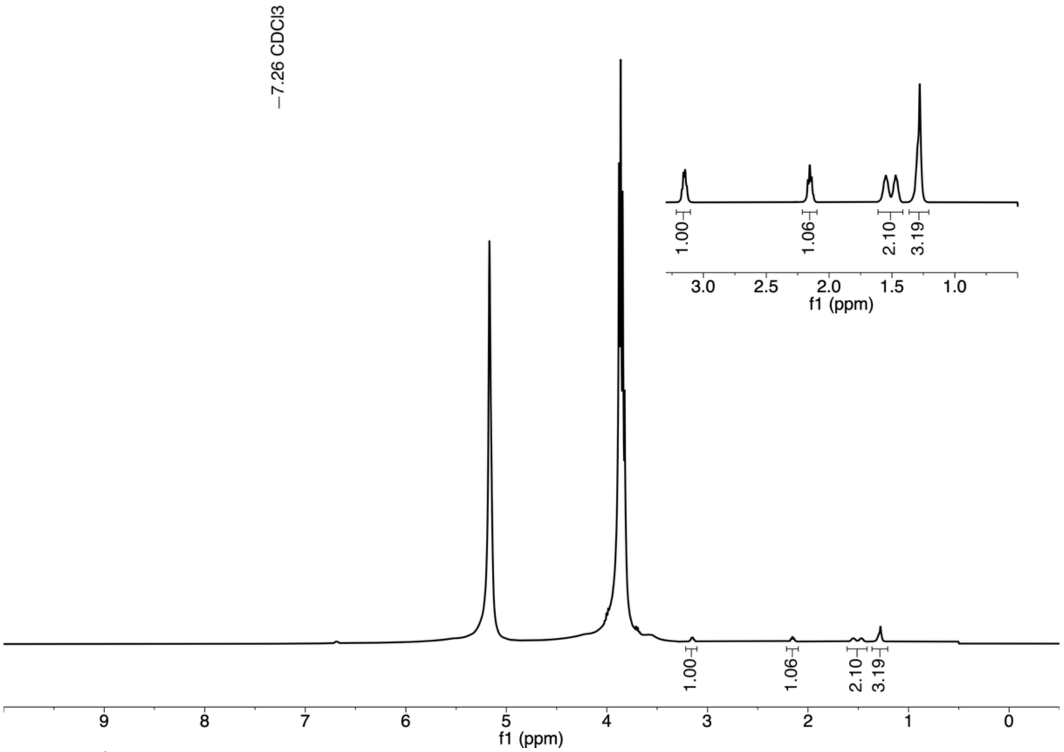

Figure S64. ^{1}H NMR of PA610 in 3:1 TFE/CDCl$_3$. The spectrum was baseline corrected between the 3.3 to 0.5 ppm region to ensure that protonated TFE signals appearing at 3.8 ppm and 5.1 ppm would not interfere with integrations.

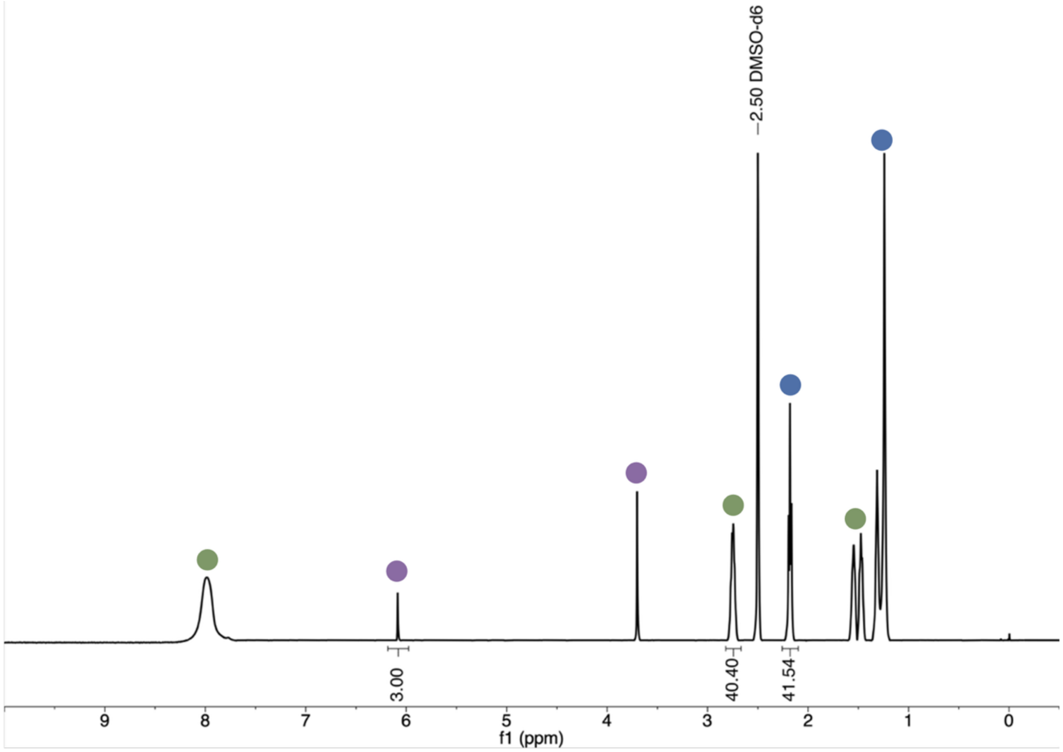

Figure S65. ^{1}H NMR of PA610 after photothermal acidic hydrolysis. TMB (purple circles), HMDA (green circles), and sebacic acid (blue circles) signals are labeled.

PPTA Photothermal Acidic Hydrolysis

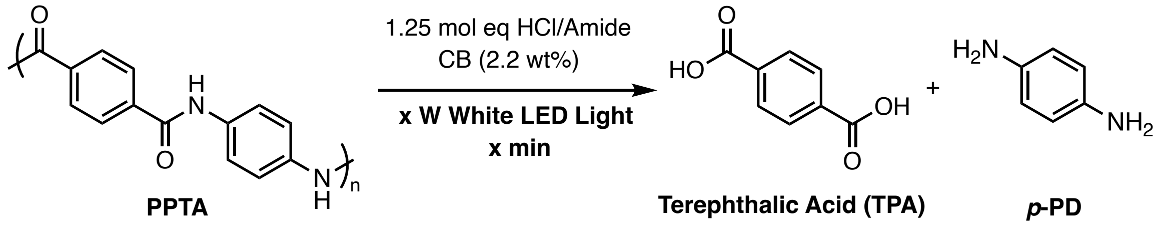

PPTA (100 mg, 0.420 mmol), CB (2.2 mg, 2.2 wt%), and 5.56 M HCl (188 μL, 1.05 mmol HCl, 1.25 molar equivalents per amide) were added to a 1-dram vial and sealed with a pressure relief cap and gently vortexed to mix. The vial was subsequently placed 0.2 mm above a 6000K white LED light (14 or 21 W) and irradiated for 10 to 240 minutes. After the reaction, the vial left to cool to room temperature, then dried under high vacuum overnight to remove any residual water. After drying, TMB (3 to 8 mg, 0.018 to 0.048 mmol) and DMSO-d_6 (0.75 mL) were added to the reaction vial and left to sit for at least 1 hour to completely dissolve. Aliquots were taken for ^{1}H NMR analysis (≤ 20 μL of the dissolved reaction mixture was diluted with 0.35 mL DMSO-d_6) shown below. *p*-Phenylenediamine (*p*-PD) was not quantified using NMR as it is light sensitive and likely degraded during irradiation.[7] Residual PPTA was not quantified.

The PPTA used in this work was Fibre Glast Kevlar pulp filler binder. As this is a commercial product purchased from Amazon.com, the purity is unknown and therefore yields are not scaled for purity.

Table S29. Results of PPTA photothermal acidic hydrolysis.

Entry	Light Intensity (W)	Time (min)	TPA Yield$_{NMR}$(%)
1	14	60	4.2
2	14	120	12.4
3	14	120	24.0
4	14	180	23.1
5	21	10	0.0
6	21	30	0.0
7	21	60	0.0

each entry is an average of 3 trials

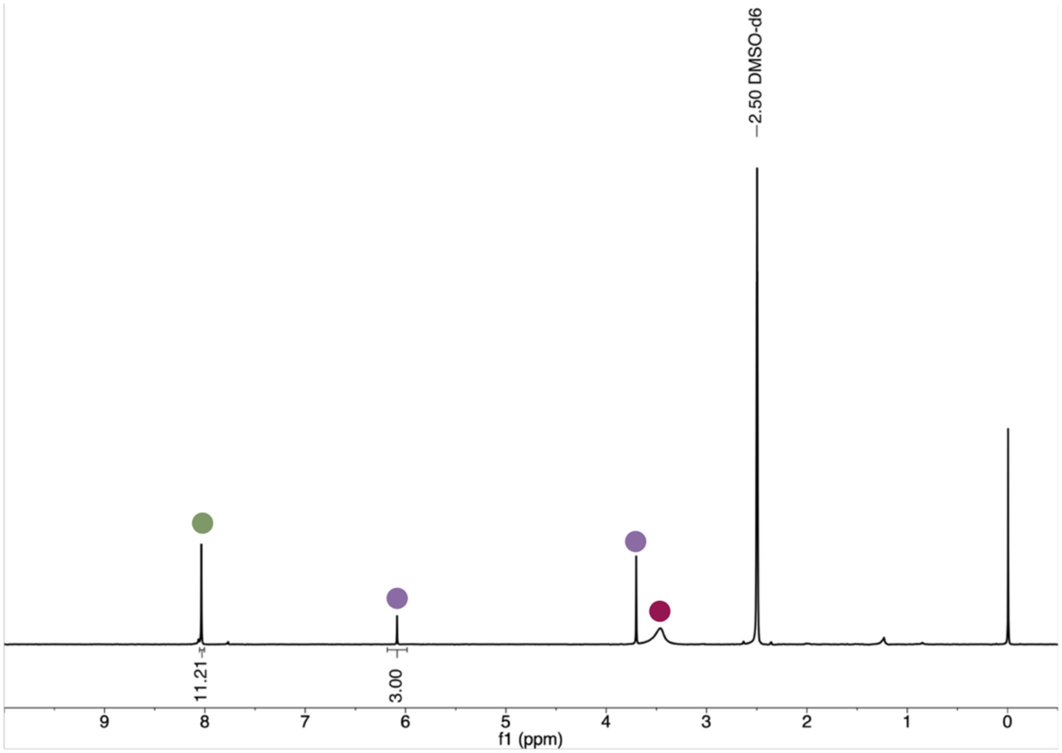

Figure S66. ^{1}H NMR of PPTA after photothermal acidic hydrolysis using 180 minutes of 14 W white LED light irradiation. TMB (purple circles), TPA (green circle), and oligomers (maroon circle) signals are labeled.

PA63T Photothermal Acidic Hydrolysis

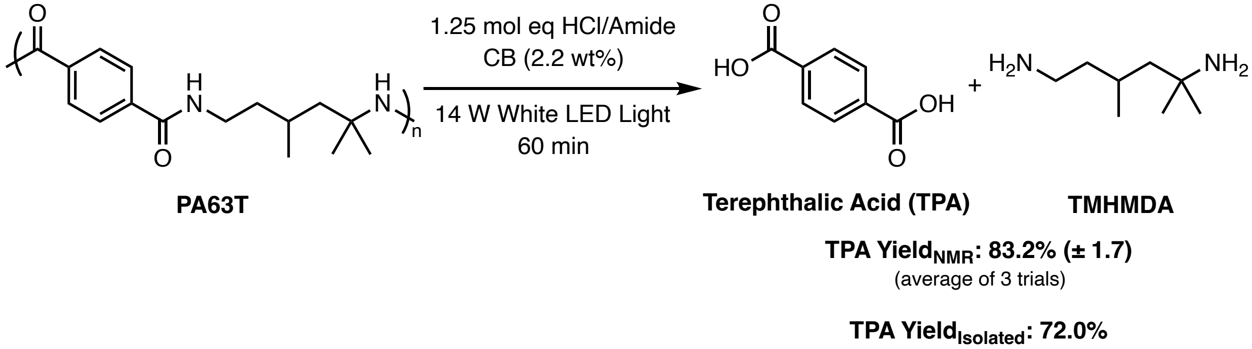

PA63T (100 mg, 0.364 mmol), CB (2.2 mg, 2.2 wt%), and 5.56 M HCl (164 µL, 0.912 mmol HCl, 1.25 mol equivalents per amide) were added to a 1-dram vial and sealed with a pressure relief cap and gently vortexed to mix. The vial was subsequently placed 0.2 mm above a 6000K white LED light (14 W) and irradiated for 60 minutes. After the reaction, the vial left to cool to room temperature, then dried under high vacuum overnight to remove any residual water. After drying, TMB (3 to 8 mg, 0.018 to 0.048 mmol) and DMSO-d_6 (0.75 mL) were added to the reaction vial and left to sit for at least 1 hour to completely dissolve. Aliquots were taken for ^{1}H NMR analysis (≤ 20 µL of the dissolved reaction mixture was diluted with 0.35 mL DMSO-d_6) shown below. Trimethyl hexamethylenediamine (TMHMDA) was not quantified using NMR due to overlapping peaks with oligomers. Residual PA63T was not quantified.

To isolate TPA from solution, a modified workup procedure was performed. After the reaction, the reaction vial was dried under high vacuum overnight to remove any residual water. Once dry, KOH (40 mg, 0.713 mmol) and H_2O (1 mL) were added to the reaction vial, and the solution was vortexed to mix. The vial was then centrifuged, and the supernatant was collected. A white solid, TPA, precipitated immediately upon the addition of HCl (200 µL, 1 mmol, 5 M) to the reaction vial. The solid was dried under vacuum overnight, weighed (43.59 mg, 0.262 mmol, 72% TPA isolated yield), and characterized *via* ^{1}H NMR analysis shown below.

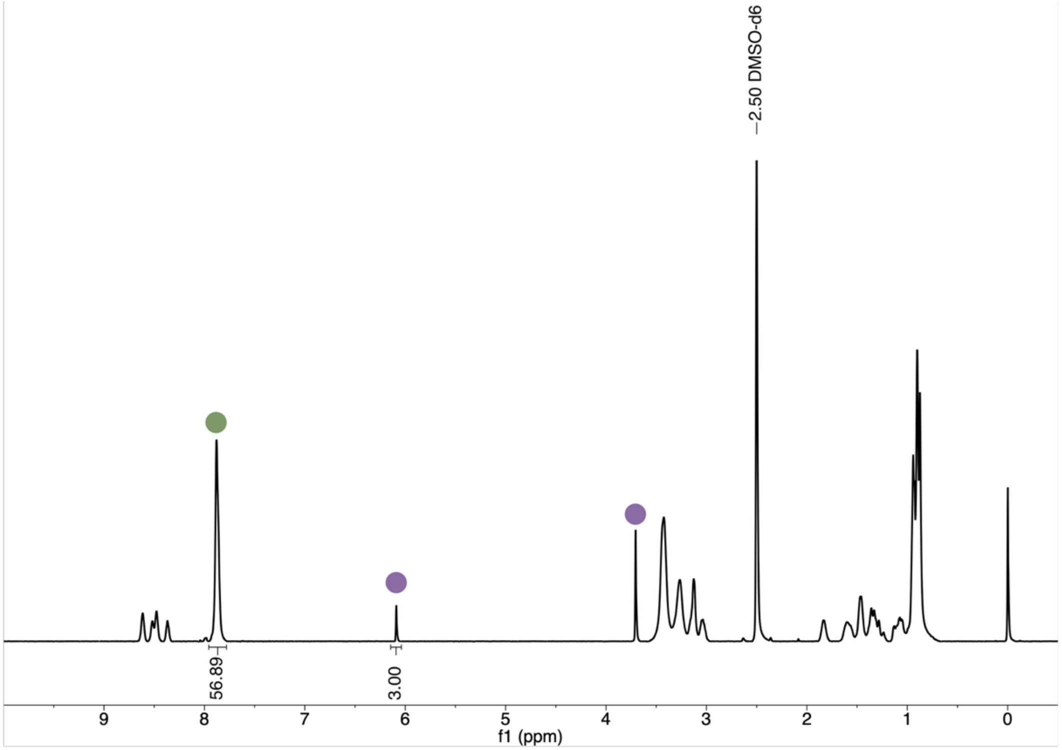

Figure S67. ^{1}H NMR of PA63T after photothermal acidic hydrolysis. TPA (green circle) and TMB (purple circles) signals are labeled. Signals between 8.8 to 8.2 ppm and 3.6 to 2.7 ppm correspond to oligomers. Signals between 2.0 to 0.5 ppm correlate to oligomers and TMHMDA.

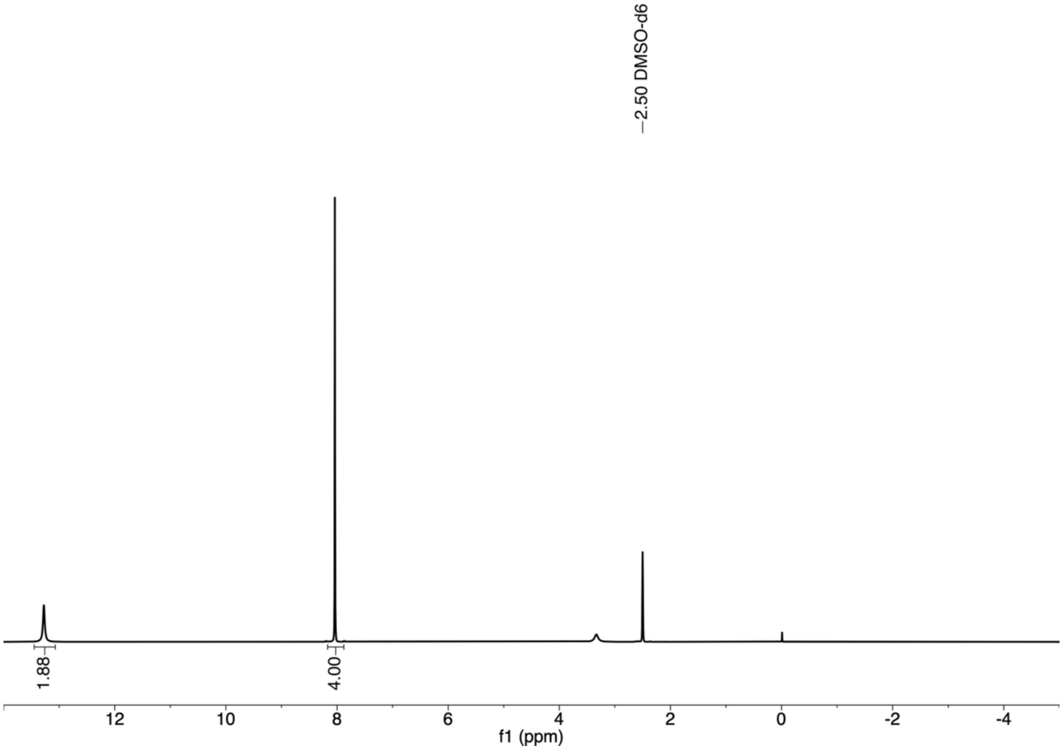

Figure S68. ^{1}H NMR of isolated TPA collected from PA63T after photothermal acidic hydrolysis. A water impurity can be seen at 3.3 ppm.

Mixed PA Photothermal Acidic Hydrolysis

Mixed PA6 and PA66 Photothermal Acidic Hydrolysis

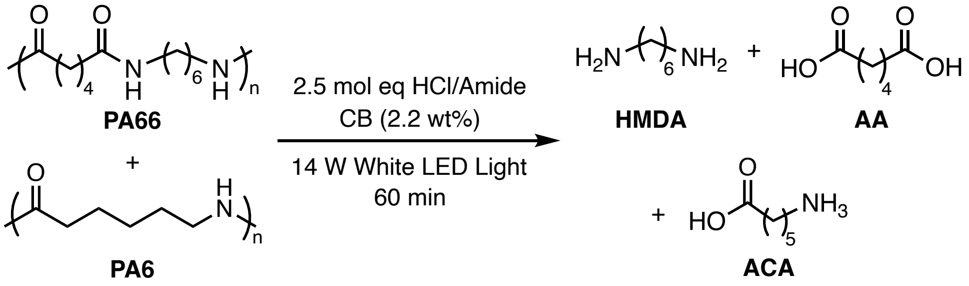

Total Monomer Recovery: 99.6% (± 4.7)

(average of 3 trials)

PA6 (50 mg, 0.442 mmol), PA66 (50 mg, 0.221 mmol), CB (2.2 mg, 2.2 wt%), and 8.8 M HCl (251 µL, 2.21 mmol HCl, 2.5 mol equiv. HCl per amide) were added to a 1-dram vial and sealed with a pressure relief cap and gently vortexed to mix. The vial was subsequently placed 0.2 mm above a 6000K white LED light (14 W) and irradiated for 60 minutes. After the reaction, the vial left to cool to room temperature, then dried under high vacuum overnight to remove any residual water. After drying, maleic acid (3 to 9 mg, 0.026 to 0.069 mmol) and D_2O (2 mL) were added to the reaction vial and left to sit for at least 1 hour to completely dissolve. Aliquots were taken for ^{1}H NMR analysis (≤ 20 µL of the dissolved reaction mixture was diluted with 0.35 mL D_2O) shown below. Residual PA6 and PA66 were not quantified. Total monomer recovery was reported instead of yields since ACA, HMDA, and AA signals overlap with each other in ^{1}H NMR analysis.

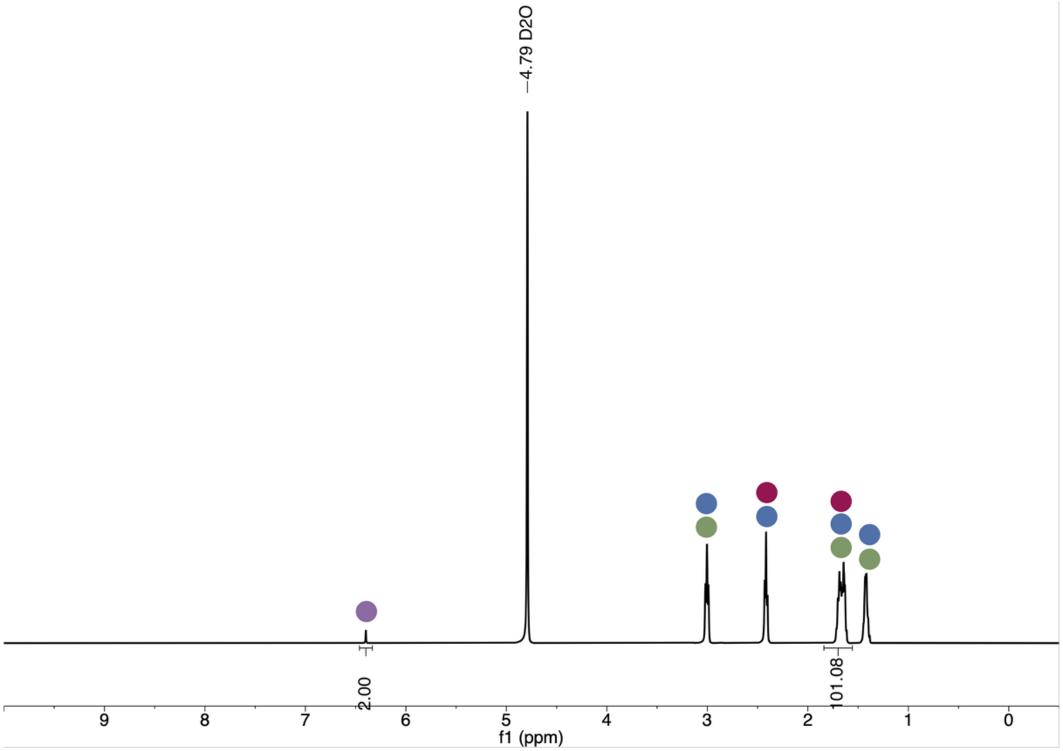

Figure S69. ^{1}H NMR of PA6 and PA66 mixture after photothermal acidic hydrolysis. Maleic acid (purple circle), HMDA (green circles), ACA (blue circles), and AA (maroon circles) signals are labeled.

Polycondensation of HMDA, AA, and ACA from Mixed PA6 and PA66 Photothermal Acidic Hydrolysis

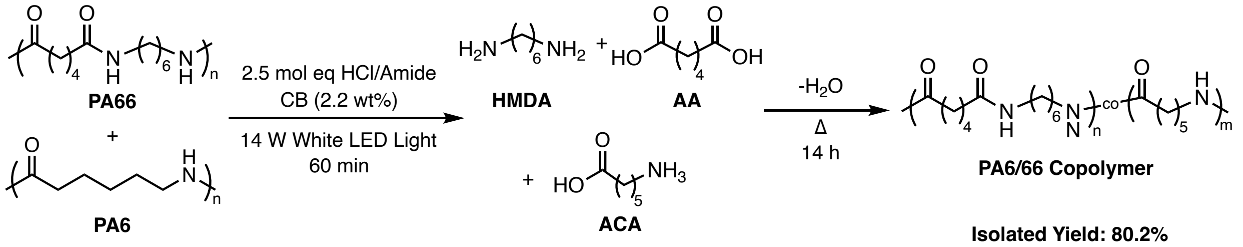

Polycondensation was performed to repolymerize monomers formed during the photothermal acidic hydrolysis of PA6 and PA66.

The photothermal acidic hydrolysis of PA6 (50 mg, 0.442 mmol) and PA66 (50 mg, 0.221 mmol) using HCl (251 µL, 2.21 mmol HCl, 2.5 mol equiv. HCl per amide) was performed using the aforementioned procedure (see section "Mixed PA6 and PA66 Photothermal Acidic Hydrolysis").

After the reaction, the vial was dried overnight under vacuum to remove any residual water. KOH (124 mg, 2.21 mmol) and H_2O (0.5 mL) were added to the dried reaction mixture to neutralize residual acid and deprotonate HMDA and ACA. The mixture was vortexed well and dried under nitrogen. To remove CB and KCl salts, hot MeOH (3 mL) was added to the mixture, then the vial was heated to 60 ºC for 10 minutes to solubilize monomers. The reaction mixture was then centrifuged for 30 seconds while still hot, and the supernatant containing the monomers was collected in a new 2 -dram glass vial then subsequently dried under vacuum. A stir bar was added to the new vial containing the dried supernatant and fitted with a PTFE septa cap. The vial was then heated under a nitrogen at 160 °C for 30 min while stirring, and 190 °C for 45 min to initiate the polycondensation. Vacuum was gradually applied for the next 30 minutes to remove any water formed during the reaction, after which the temperature was increased to 220 ºC for 12 hours to improve molecular weight.

Once the reaction was complete, the vial was backfilled with nitrogen and cooled using a dry ice-acetone bath. The vial was allowed to thaw to room temperature, and HFIP (3 mL) was added and mixed well to dissolve polymer. The remaining polymerization reaction mixture was precipitated in cold MeOH (75 mL) and the precipitate was collected and dried under vacuum overnight. The resulting solid polymer product was weighed (80.2 mg, 80.2% isolated yield) and characterized using ^{1}H and ^{13}C NMR analysis in 3:1 TFE/$CDCl_3$.

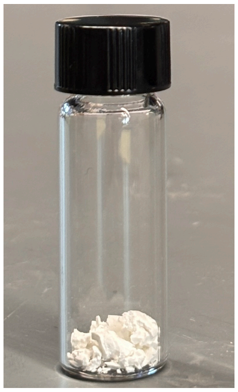

Figure S70. Isolated PA6/66 copolymer from the polycondensation reaction using AA, HMDA, and ACA from the mixed PA6 and PA66 photothermal hydrolysis.

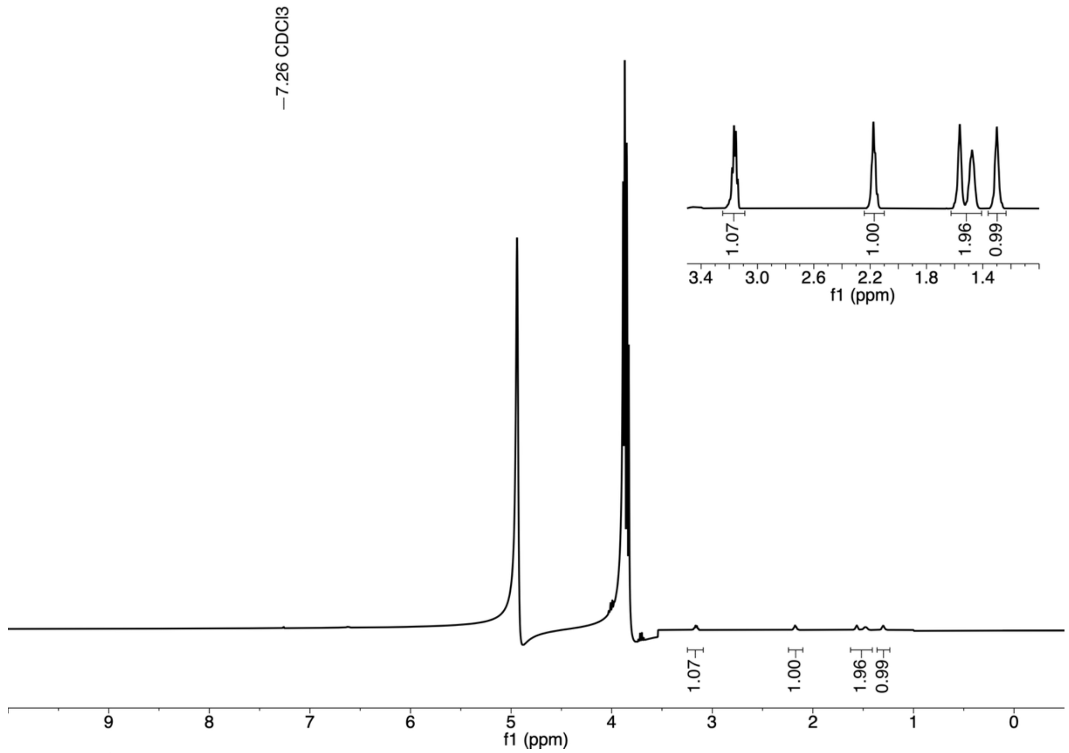

Figure S71. ^{1}H NMR of synthesized PA6/66 copolymer *via* polycondensation of HMDA, AA, and ACA from the mixed PA6 and PA66 photothermal hydrolysis. The spectrum was baseline corrected between the 3.5 to 1.0 ppm region to ensure that protonated TFE signals appearing at 3.9 ppm and 4.9 ppm would not interfere with integrations.

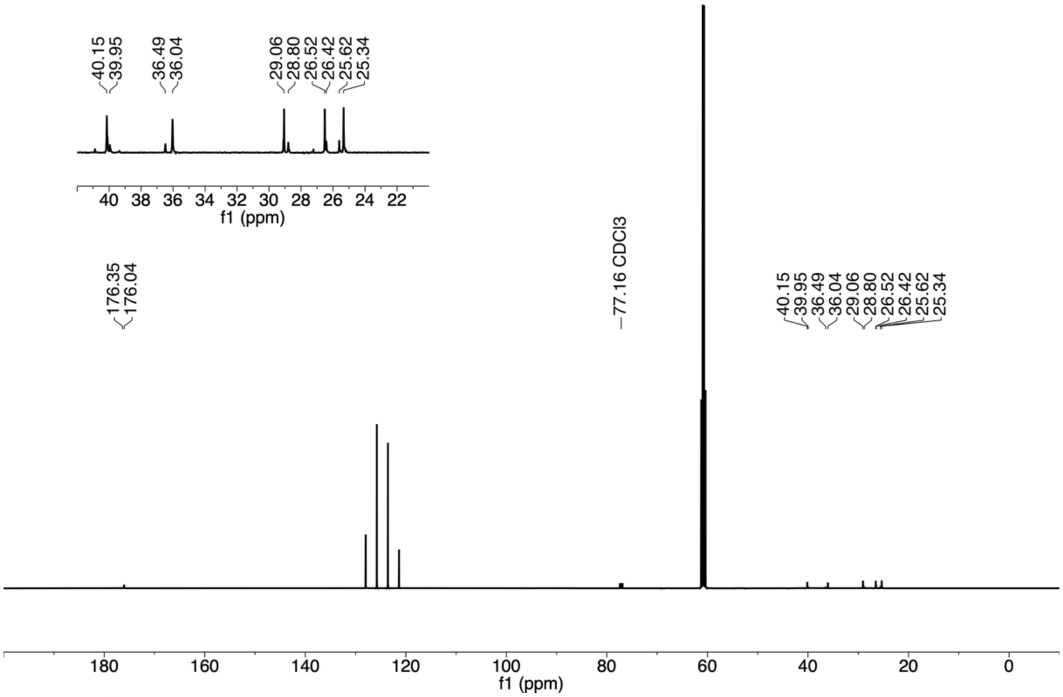

Figure S72. ^{13}C NMR of synthesized PA6/66 copolymer *via* polycondensation of HMDA, AA, and ACA from the mixed PA6 and PA66 photothermal hydrolysis. TFE peaks can be seen at 124 and 60 ppm. PA6/66 signals match well with previous literature assignments.[8]

Mixed PA11 and PA12 Photothermal Acidic Hydrolysis

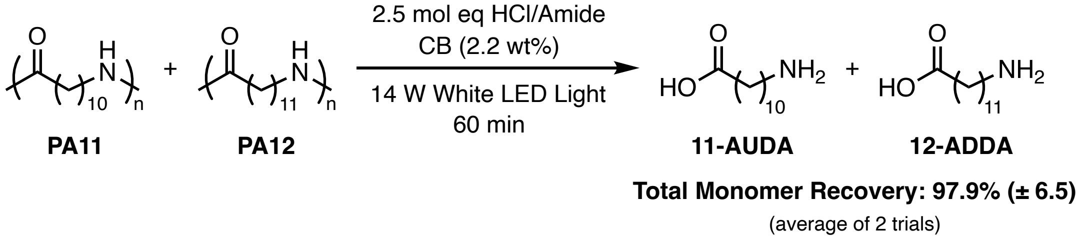

PA11 (50 mg, 0.273 mmol), PA12 (50 mg, 0.253 mmol), CB (2.2 mg, 2.2 wt%), and 8.8 M HCl (150 µL, 1.32 mmol HCl, 2.5 mol equiv. HCl per amide) were added to a 1-dram vial and sealed with a pressure relief cap and gently vortexed to mix. The vial was subsequently placed 0.2 mm above a 6000K white LED light (14 W) and irradiated for 60 minutes. After the reaction, the vial left to cool to room temperature, then dried under high vacuum overnight to remove any residual water. After drying, (3 to 8 mg, 0.018 to 0.048 mmol) and DMSO-d_6 (0.75 mL) were added to the reaction vial and left to sit for at least 1 hour to completely dissolve. Residual PA11 and PA12 were not quantified. Aliquots were taken for ^{1}H NMR analysis (≤ 20 µL of the dissolved reaction mixture was diluted with 0.35 mL DMSO-d_6) shown below.

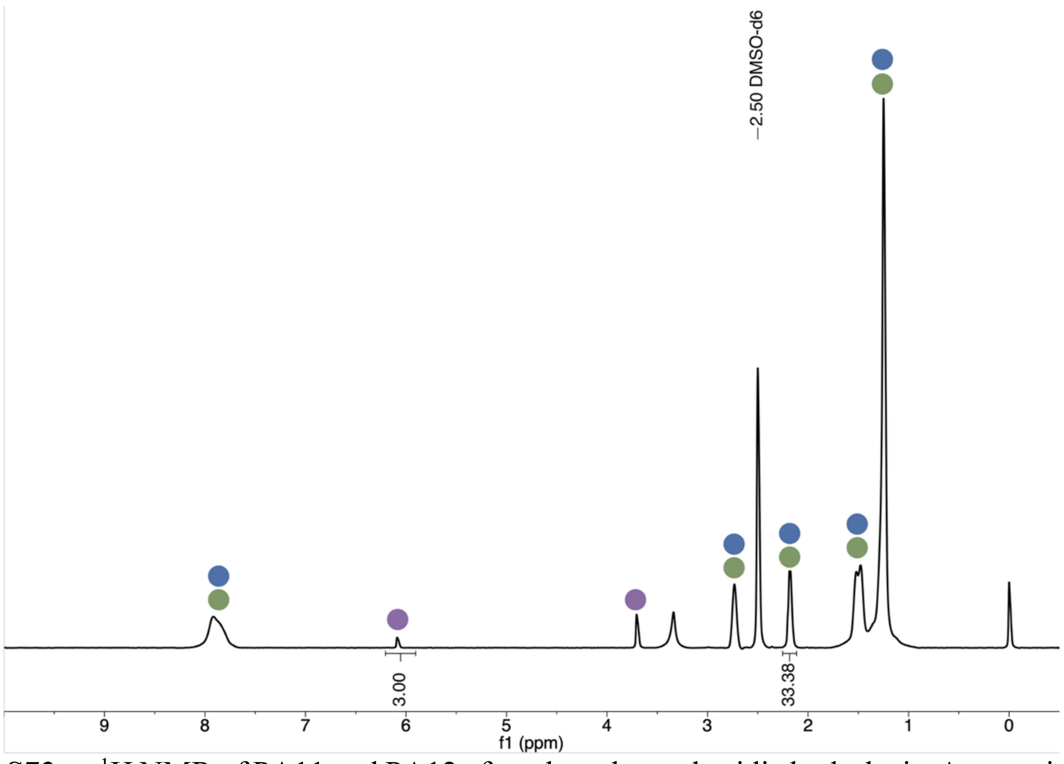

Figure S73. ^{1}H NMR of PA11 and PA12 after photothermal acidic hydrolysis. A water impurity can be seen at 3.3 ppm. 11-AUDA (blue circles), 12-ADDA (green circles), and TMB (purple circles) signals are labeled.

Mixed PA66 and PA610 Photothermal Acidic Hydrolysis

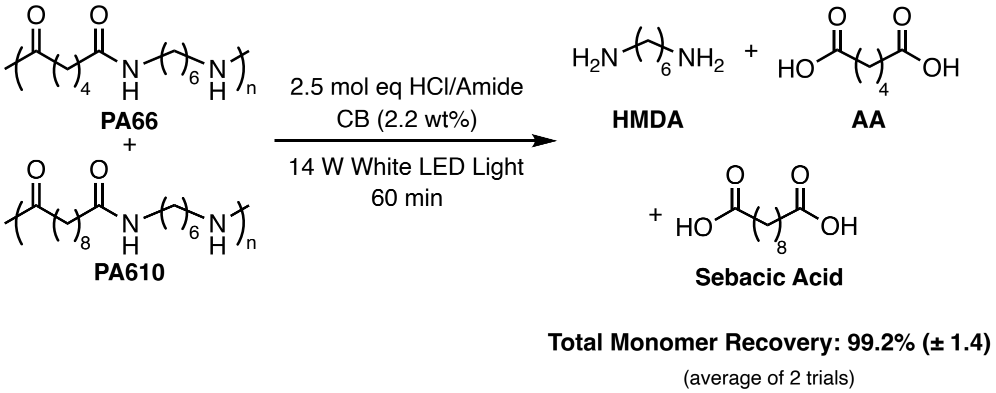

PA66 (50 mg, 0.221 mmol), PA610 (50 mg, 0.177 mmol), CB (2.2 mg, 2.2 wt%), and 8.8 M HCl (226 µL, 1.99 mmol HCl, 2.5 mol equiv. HCl per amide) were added to a 1-dram vial and sealed with a pressure relief cap and gently vortexed to mix. The vial was subsequently placed 0.2 mm above a 6000K white LED light (14 W) and irradiated for 60 minutes. After the reaction, the vial left to cool to room temperature, then dried under high vacuum overnight to remove any residual water. After drying, (3 to 8 mg, 0.018 to 0.048 mmol) and DMSO-d_6 (0.75 mL) were added to the reaction vial and left to sit for at least 1 hour to completely dissolve. Residual PA66 and PA610 were not quantified. Aliquots were taken for ^1H NMR analysis (≤ 20 µL of the dissolved reaction mixture was diluted with 0.35 mL DMSO-d_6) shown below.

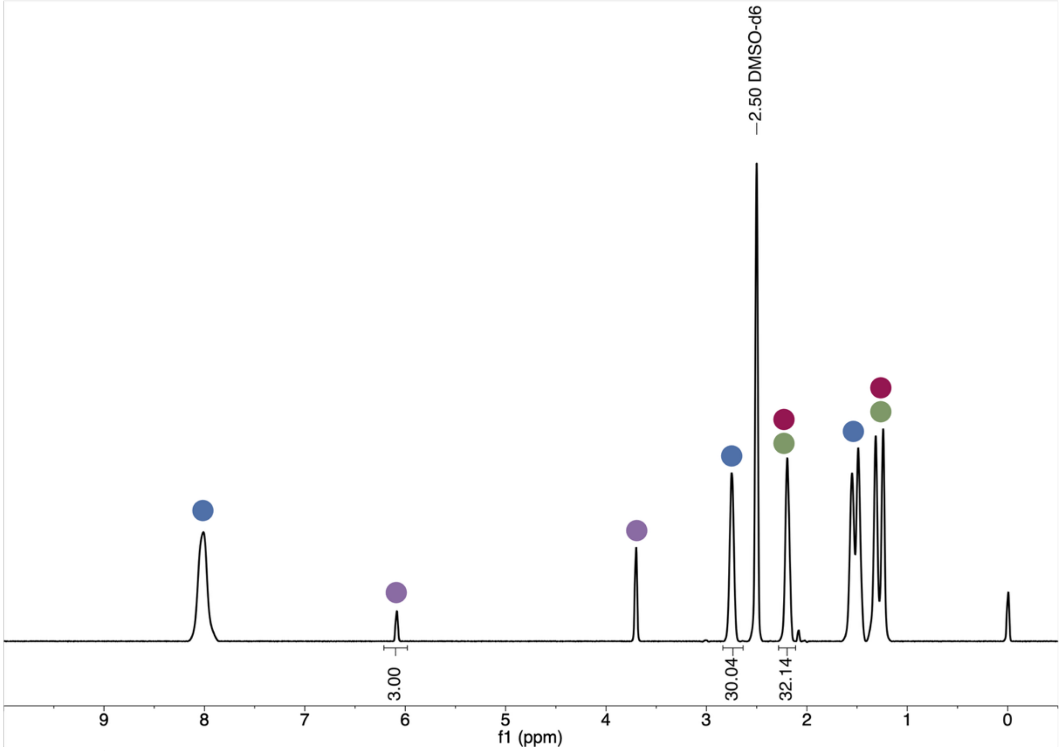

Figure S74. ^1H NMR of PA66 and PA610 after photothermal acidic hydrolysis. HMDA (blue circles), TMB (purple circles), AA (green circles), and sebacic acid (maroon circles) signals are labeled.

Post-Consumer PA6 Photothermal Ring-Closing Depolymerization

Purity Determination for PA6 Post-Consumer Samples

In a 1-dram glass vial, ~20 mg of post-consumer PA6 samples were dissolved in a mixture of 3:1 TFE/$CDCl_3$ alongside a known amount of dimethyl sulfone ($DMSO_2$) as an internal standard. Aliquots were taken for ^1H NMR, and purity results are shown below. ^1H NMR spectra were baseline corrected between the 3.5 to 0.5 ppm region to ensure that protonated TFE signals appearing between 3.8 and 5.3 ppm would not interfere with qNMR purity determinations.

Table S30. Sample purity information for post-consumer PA6 samples.

Entry	Sample Name	Sample Mass (mg)	Standard Mass (mg)	PA6 Purity (%)
1	PA6 T-shirt	19.6	4.6	79.2
2	PA6 Tubing	22.7	5.5	88.0
3	PA6 Black Carpet	21.1	5.8	88.7
4	PA6 Beige Carpet	19.6	4.6	85.8
5	PA6 Thread	18.0	5.2	83.5

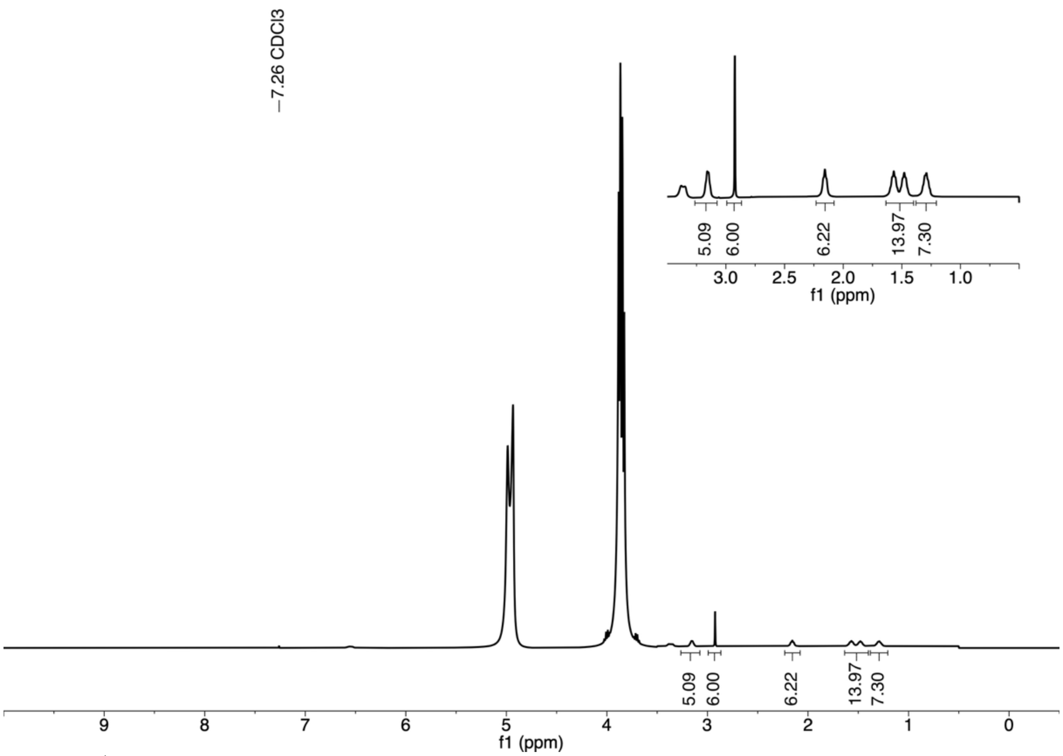

Figure S75. ^1H NMR of post-consumer PA6 T-shirt. Protonated TFE peaks appear at 3.8 ppm and 4.9 ppm. $DMSO_2$ signal appears at 2.9 ppm.

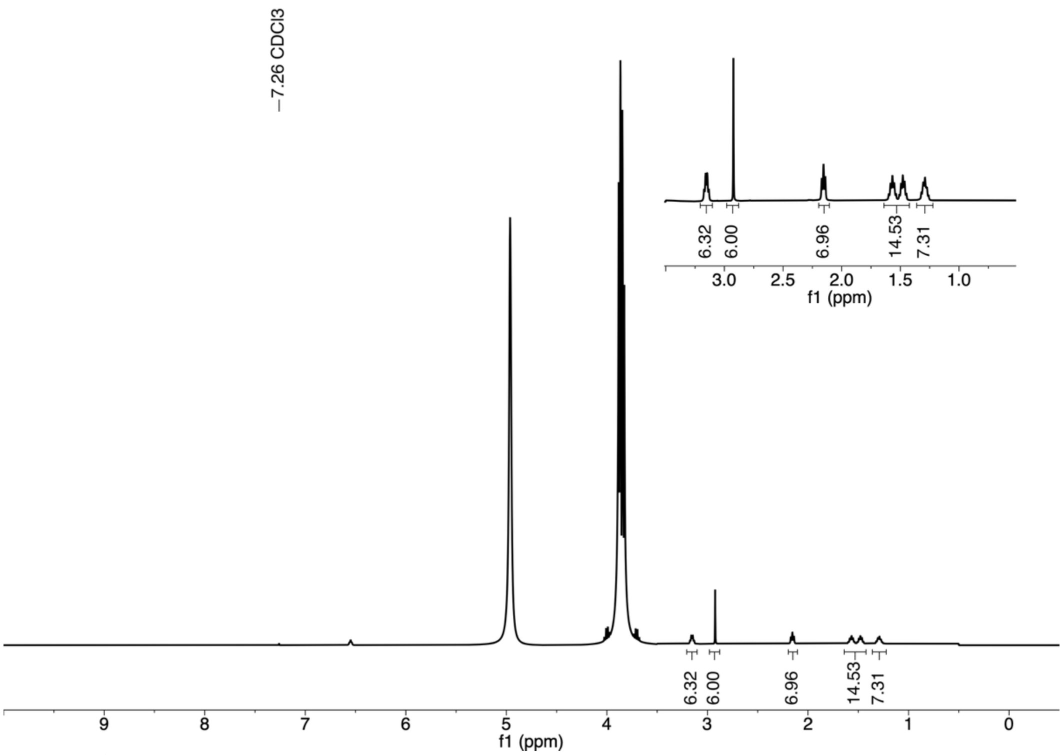

Figure S76. ^{1}H NMR of post-consumer PA6 tubing. Protonated TFE peaks appear at 3.8 ppm and 4.9 ppm. $DMSO_2$ signal appears at 2.9 ppm.

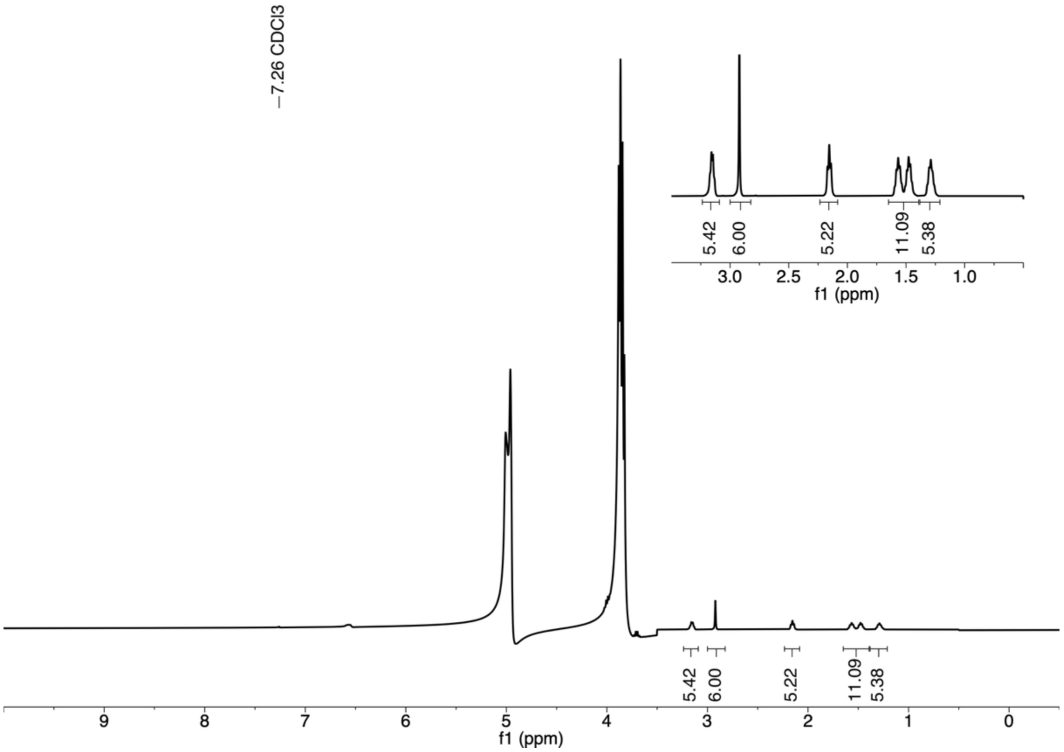

Figure S77. ^{1}H NMR of post-consumer PA6 black carpet. Protonated TFE peaks appear at 3.8 ppm and 4.9 ppm. $DMSO_2$ signal appears at 2.9 ppm.

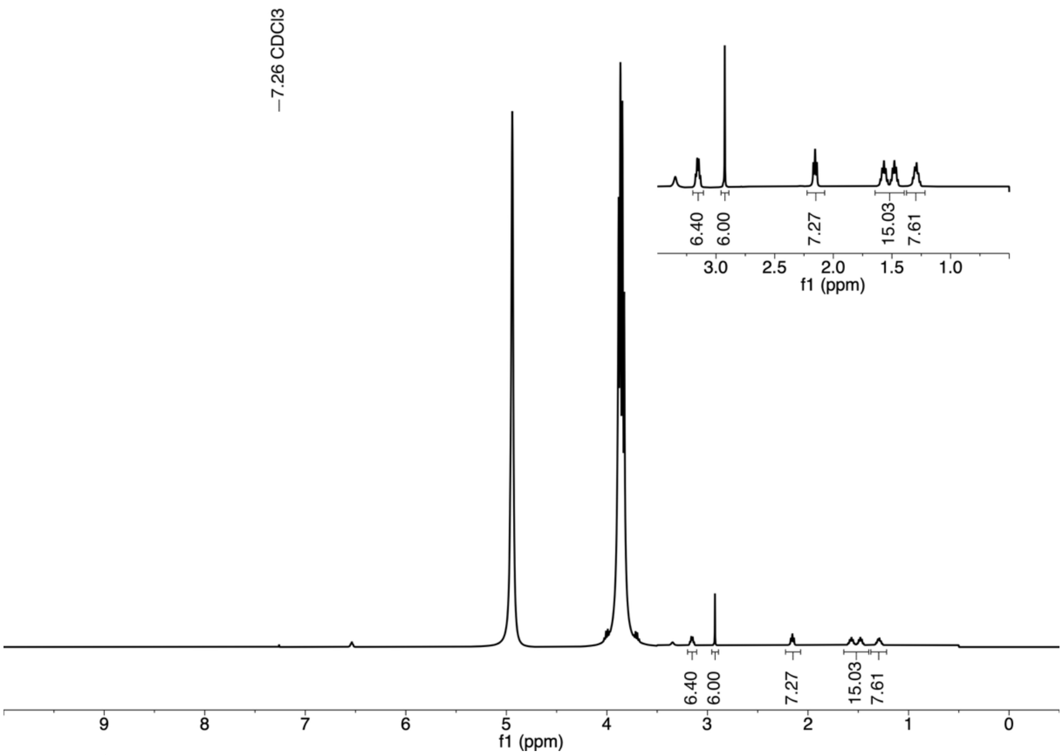

Figure S78. ^{1}H NMR of post-consumer PA6 beige carpet. Protonated TFE peaks appear at 3.8 ppm and 4.9 ppm. $DMSO_2$ signal appears at 2.9 ppm.

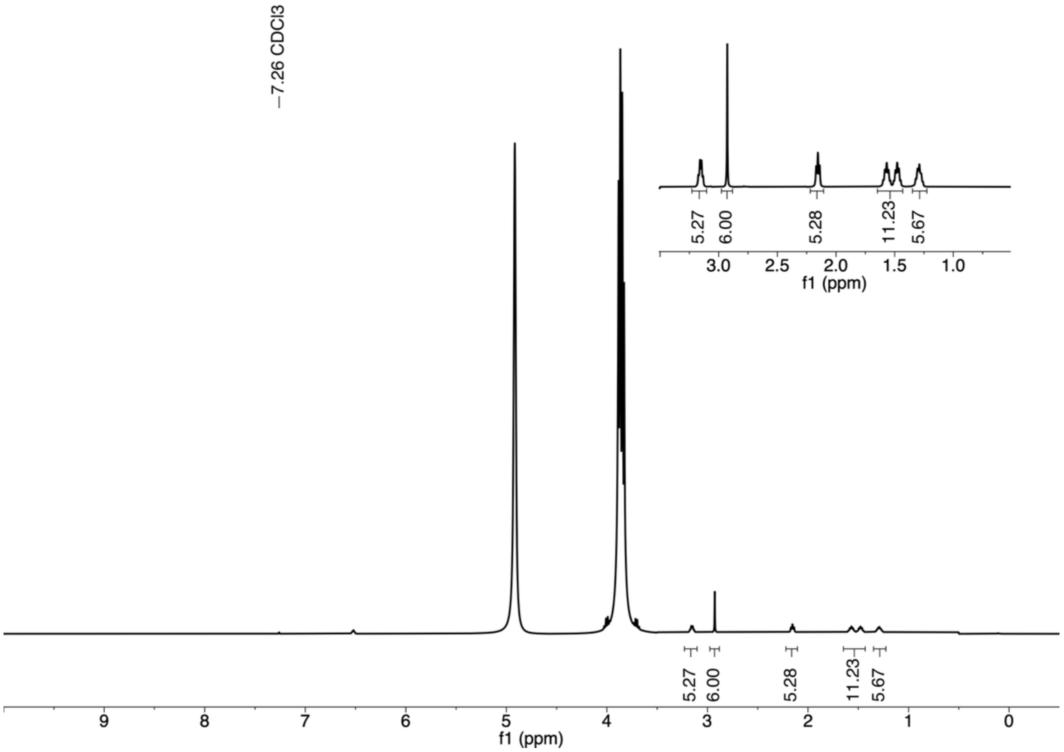

Figure S79. ^{1}H NMR of post-consumer PA6 thread. Protonated TFE peaks appear at 3.8 ppm and 4.9 ppm. $DMSO_2$ signal appears at 2.9 ppm.

Photothermal Ring-Closing Depolymerization of PA6 Post-Consumer Samples

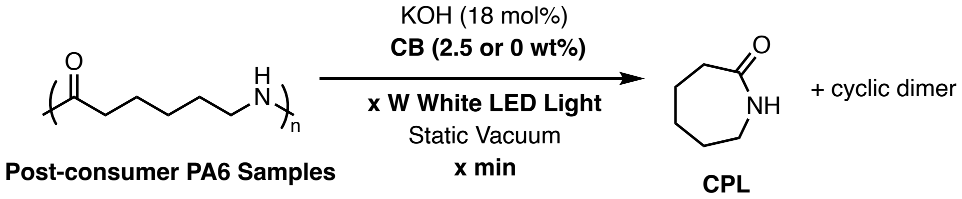

The procedure was modified from the general PA6 ring-closing depolymerization procedure, where post-consumer samples were used instead of purchased PA66 powder. All post-consumer samples were cut to approximately 1 to 2 mm pieces prior to depolymerization. Depolymerization reactions were performed at 21 W for 10 minutes or 14 W for 60 minutes and had either 0 or 2.5 wt% CB. ^{1}H NMR analysis was performed in $CDCl_3$, and leftover PA6 was not quantified. The reaction workup was the same as the general PA6 photothermal depolymerization procedure. The depolymerization results are summarized below.

Table S31. Results of post-consumer PA6 photothermal ring-closing depolymerization with 2.5 wt% CB.

Entry	PA6 Sample	Light Intensity (W)	Time (min)	PA6 Purity (%)	CPL $Yield_{NMR}$(%)	Dimer $Yield_{NMR}$ (%)
1	PA6 T-shirt	21	10	79.2	45.2	3.9
2	PA6 Tubing	21	10	88.0	63.4	2.1
3	PA6 Black Carpet	21	10	88.7	63.5	2.7
4	PA6 Beige Carpet	21	10	85.8	43.4	3.4
5	PA6 Thread	21	10	83.1	45.4	2.4
6	PA6 T-shirt	14	60	79.2	70.0	1.8
7	PA6 Tubing	14	60	88.0	33.8	1.2
8	PA6 Black Carpet	14	60	88.7	72.6	1.8
9	PA6 Beige Carpet	14	60	85.8	70.3	2.1
10	PA6 Thread	14	60	83.1	65.9	1.6

Each entry is an average of 2 trials. All yields are scaled with PA6 purity.

Table S32. Results of post-consumer PA6 photothermal ring-closing depolymerization with 0 wt% CB.

Entry	PA6 Sample	Light Intensity (W)	Time (min)	PA6 Purity (%)	CPL $Yield_{NMR}$(%)	Dimer $Yield_{NMR}$ (%)
1	PA6 T-shirt	14	60	79.2	48.6	1.0
2	PA6 Tubing	21	10	88.0	73.7	2.5
3	PA6 Black Carpet	14	60	88.7	19.8	0.7
4	PA6 Beige Carpet	14	60	85.8	1.4	0.1
5	PA6 Thread	14	60	83.1	0.0	0.0

Each entry is an average of 2 trials. All yields are scaled with PA6 purity.

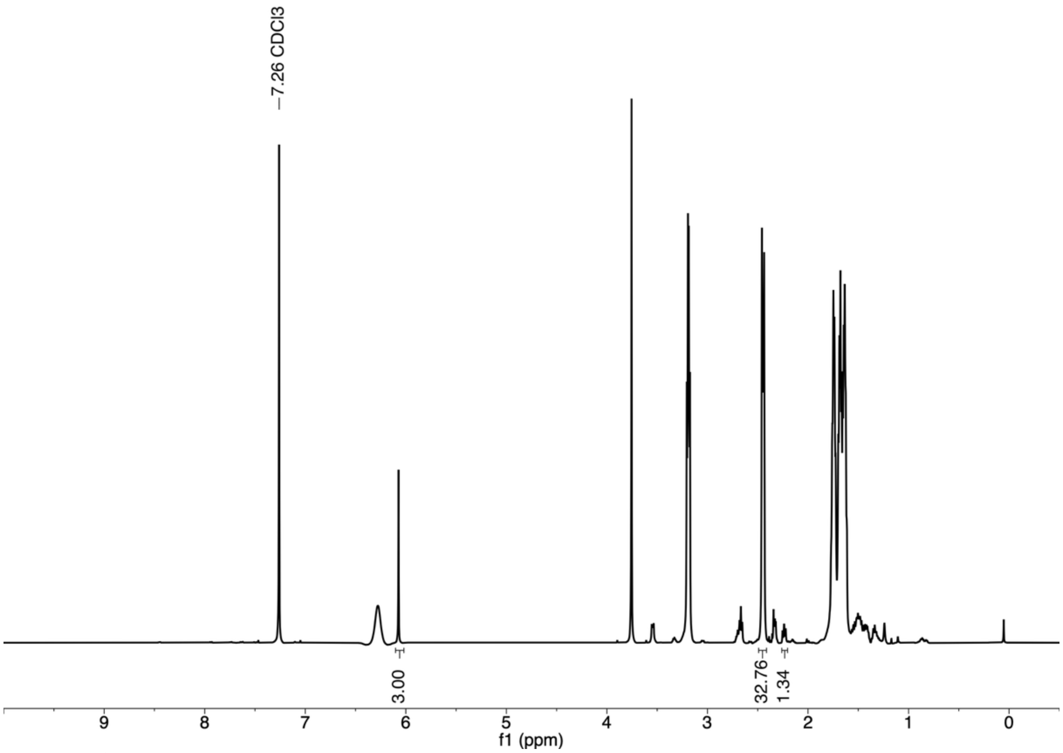

Figure S80. ^{1}H NMR of post-consumer PA6 T-shirt after photothermal ring-closing depolymerization with 2.5 wt% CB.

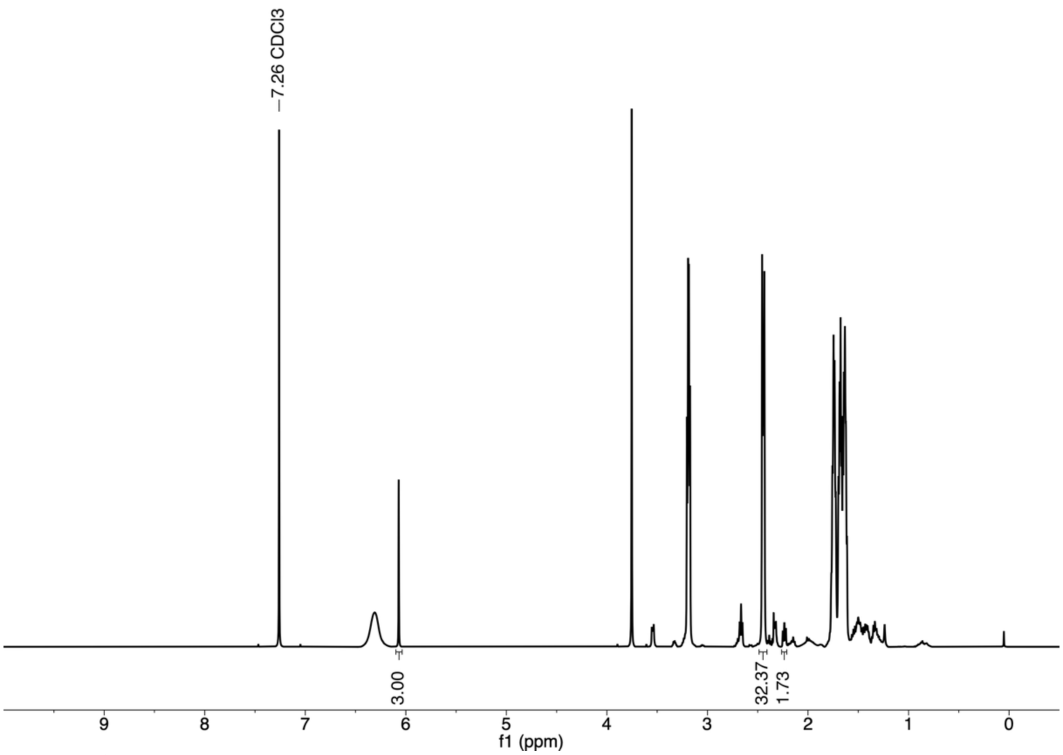

Figure S81. ^{1}H NMR of post-consumer PA6 tubing after photothermal ring-closing depolymerization with 2.5 wt% CB.

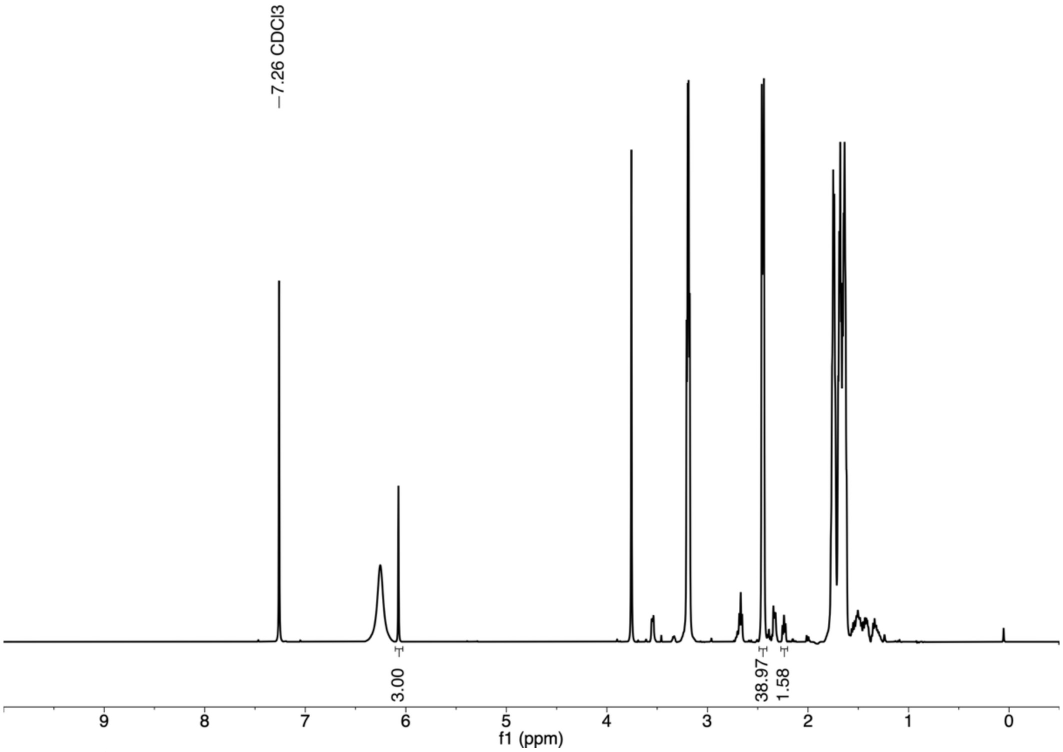

Figure S82. ^{1}H NMR of post-consumer PA6 black carpet after photothermal ring-closing depolymerization with 2.5 wt% CB.

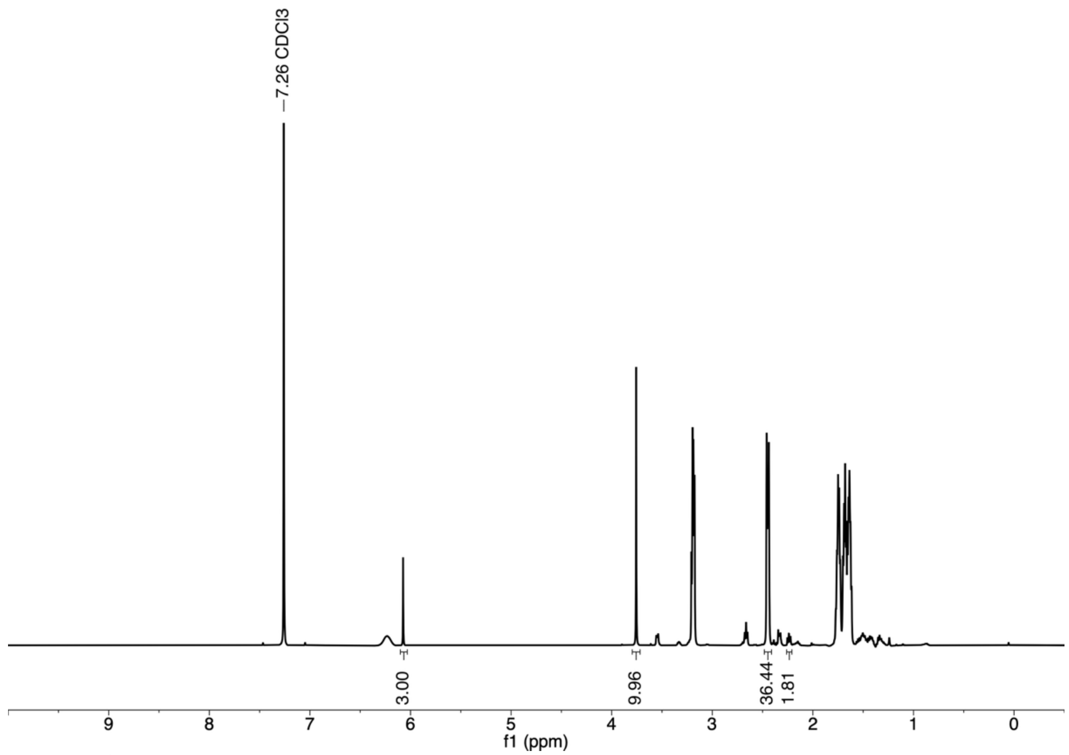

Figure S83. ^{1}H NMR of post-consumer PA6 beige carpet after photothermal ring-closing depolymerization with 2.5 wt% CB.

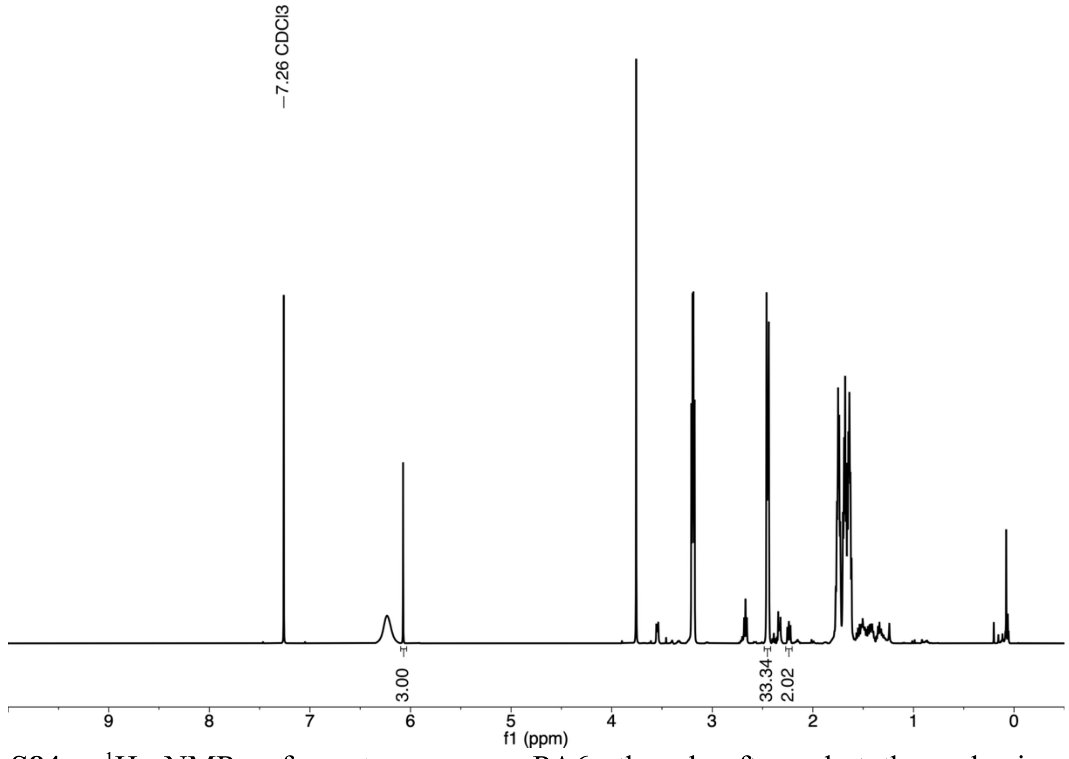

Figure S84. ^{1}H NMR of post-consumer PA6 thread after photothermal ring-closing depolymerization with 2.5 wt% CB.

Photothermal Ring-Closing Depolymerization of PA6 T-shirt Using Focused Sunlight Irradiation

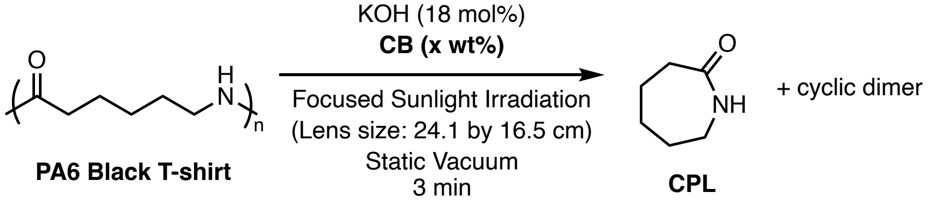

The procedure was slightly modified from the Focused Sunlight PA6 ring-closing depolymerization procedure, where a black PA6 T-shirt was used instead of purchased PA66 powder. A smaller Fresnel lens was used (24.1 cm x 16.5 cm), and reactions had either 0 or 2.5 wt% CB. The focused sunlight light intensity per unit area was determined to be 17.7 W/cm^2 (see section entitled "Photothermal Reaction Setup" for calculation). ^{1}H NMR analysis was performed in $CDCl_3$, and leftover PA6 was not quantified. The reaction workup was the same as the general PA6 photothermal depolymerization procedure. The depolymerization results are summarized below.

Table S33. Results of post-consumer PA6 photothermal ring-closing depolymerization using focused sunlight as a light source.

Entry	CB Loading (wt%)	PA6 Purity (%)	CPL Yield$_{NMR}$ (%)	Dimer Yield$_{NMR}$ (%)
1	2.5	79.2	70.0	2.7
2	0	79.2	57.9	2.7

Each entry is an average of 2 trials. All yields are scaled with PA6 purity.

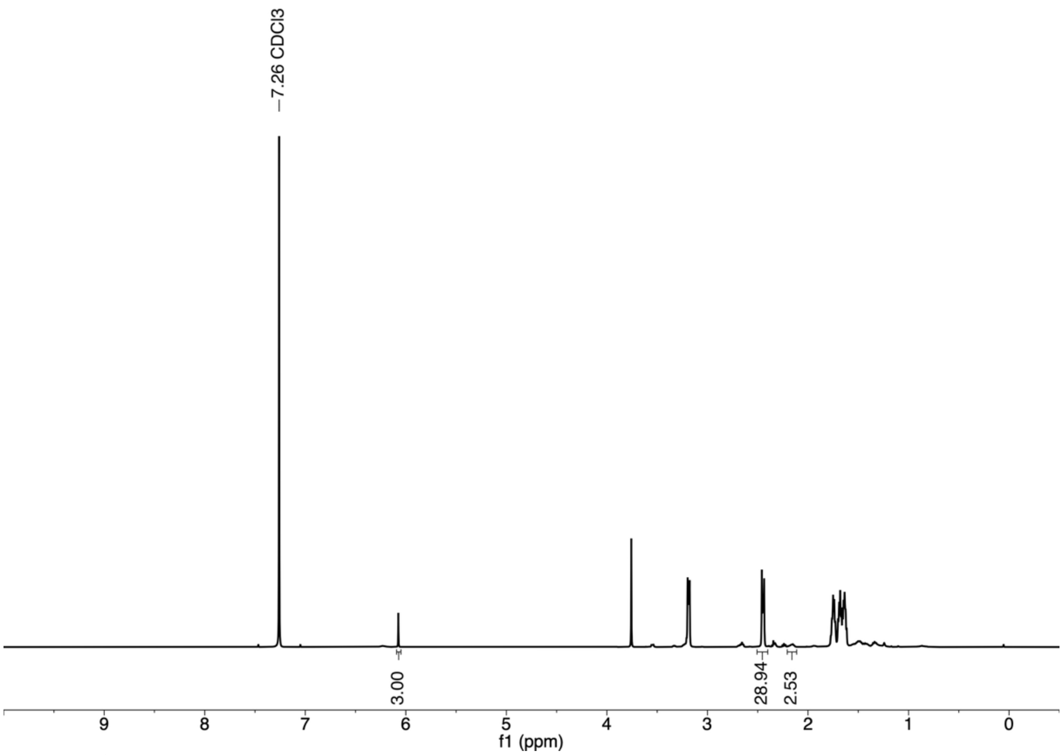

Figure S85. ^{1}H NMR of post-consumer PA6 T-shirt after photothermal ring-closing depolymerization using focused sunlight irradiation as a light source.

Photothermal Ring-Closing Depolymerization of Mixed Post-Consumer PA6

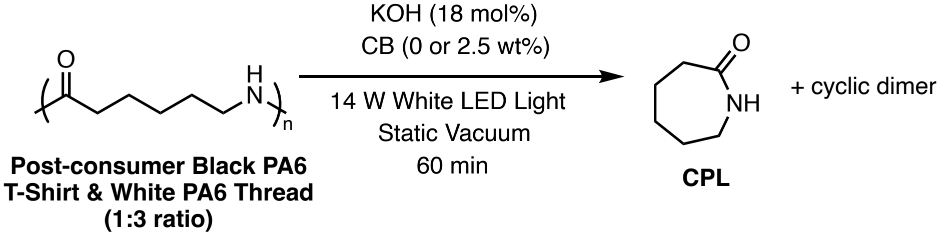

The procedure was slightly modified from the post-consumer PA6 ring-closing depolymerization procedure, where a 1:3 mixture of black PA6 T-shirt and white PA6 thread was used as the starting material and either 0 or 2.5 wt% CB was added. ^1H NMR analysis was performed in $CDCl_3$, and leftover PA6 was not quantified. The reaction workup was the same as the general PA6 photothermal depolymerization procedure. The depolymerization results are summarized below.

Table S34. Results of mixed post-consumer PA6 photothermal ring-closing depolymerization.

Entry	CB (wt %)	PA6 Purity (%)	CPL $Yield_{NMR}$(%)	Dimer $Yield_{NMR}$ (%)
1	0	82.1	42.1	0.9
2	2.5	82.1	50.4	1.4

Each entry is an average of 2 trials. PA6 purity for mixed post-consumer samples is scaled for amount of each PA6 sample's purity. All yields are scaled with PA6 purity.

Post-Consumer PA66 Photothermal Acidic Hydrolysis

Purity Determination for PA66 Post-Consumer Samples

In a 1-dram glass vial, ~12-25 mg of post-consumer PA66 samples were dissolved in a mixture of 3:1 TFE/$CDCl_3$ alongside a known amount of $DMSO_2$ as an internal standard. Aliquots were taken for ^1H NMR analysis, and purity results are shown below. ^1H NMR spectra were baseline corrected between the 3.5 to 0.5 ppm region to ensure that protonated TFE signals appearing between 3.8 ppm and 5.3 ppm would not interfere with qNMR purity determinations.

Table S35. Sample purity information for post-consumer PA66 samples.

Entry	Sample Name	Sample Mass (mg)	Standard Mass (mg)	PA66 Purity (%)
1	PA66 Thread	19.0	5.2	81.4
2	PA66 Black Zip Ties	19.9	4.7	91.9
3	PA66 White Zip Ties	14.3	8.0	95.2
4	PA66 Multicolored Zip Ties	14.4	4.1	87.1
5	PA66 Red Zip Ties	17.6	4.5	92.8
6	PA66 Green Zip Ties	21.5	6.6	92.7

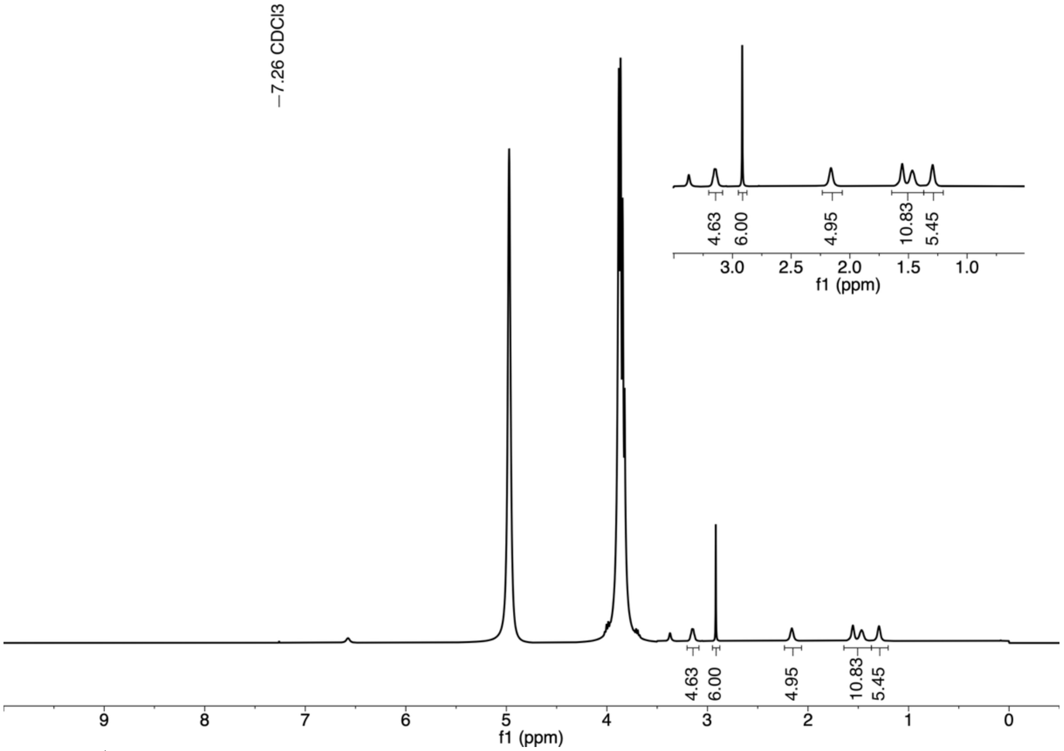

Figure S86. ^1H NMR of post-consumer PA66 thread. Protonated TFE peaks appear at 3.8 ppm and 4.9 ppm. $DMSO_2$ signal appears at 2.9 ppm.

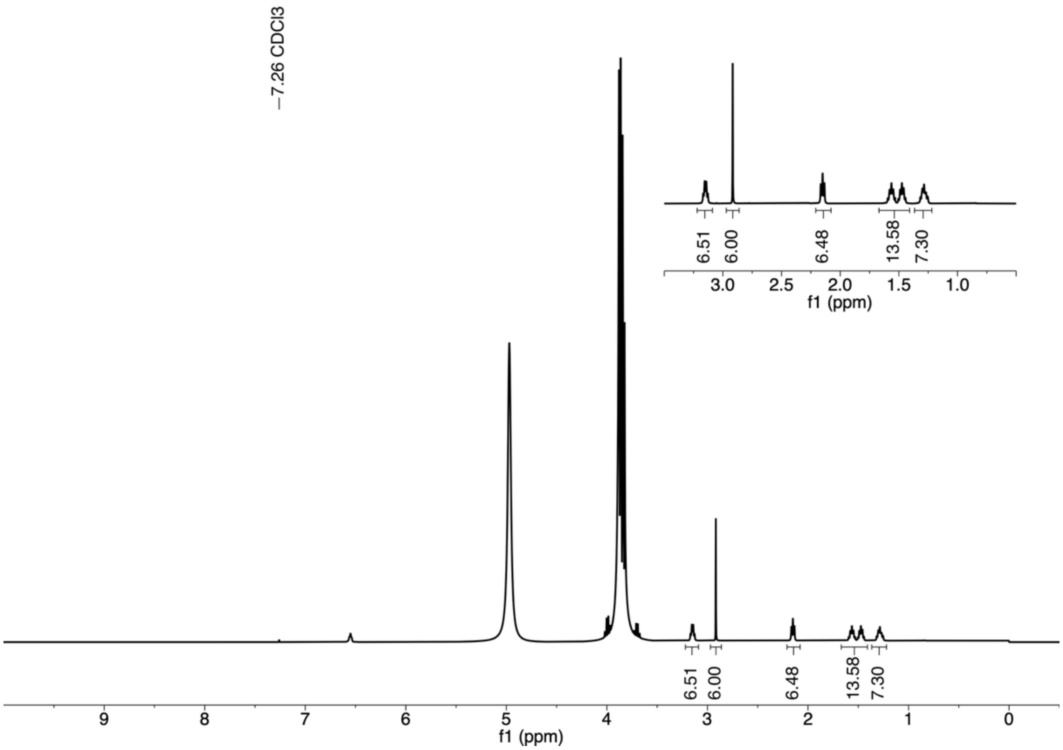

Figure S87. ^{1}H NMR of post-consumer PA66 black zip ties. Protonated TFE peaks appear at 3.8 ppm and 4.9 ppm. $DMSO_2$ signal appears at 2.9 ppm.

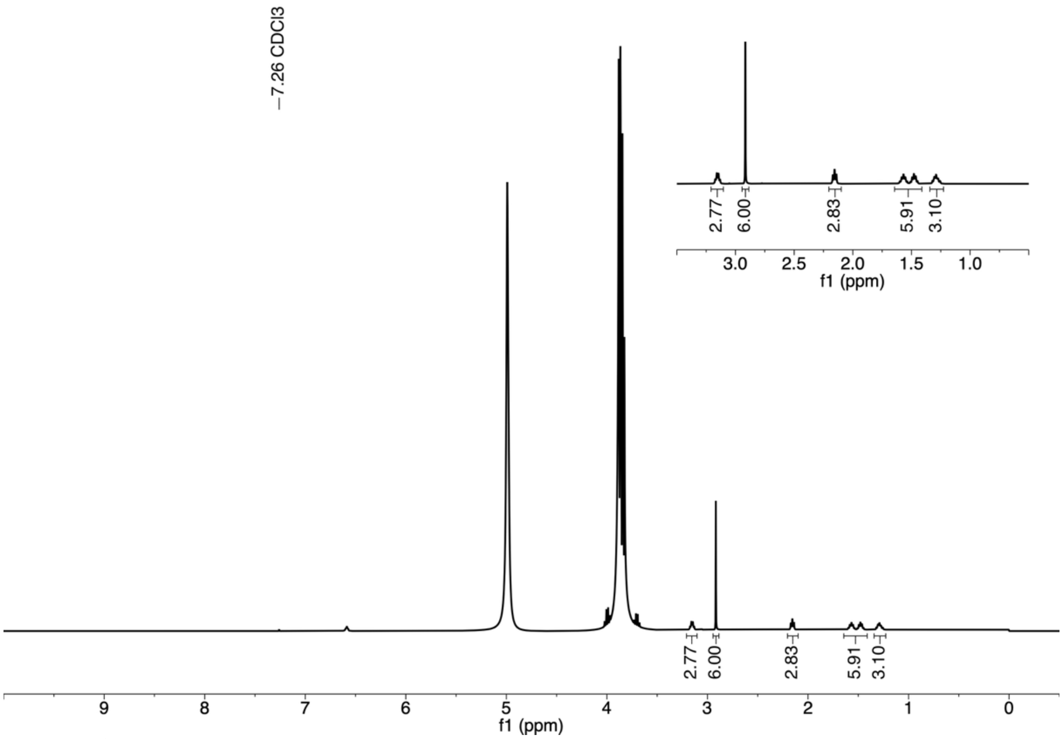

Figure S88. ^{1}H NMR of post-consumer PA66 white zip ties. Protonated TFE peaks appear at 3.8 ppm and 4.9 ppm. $DMSO_2$ signal appears at 2.9 ppm.

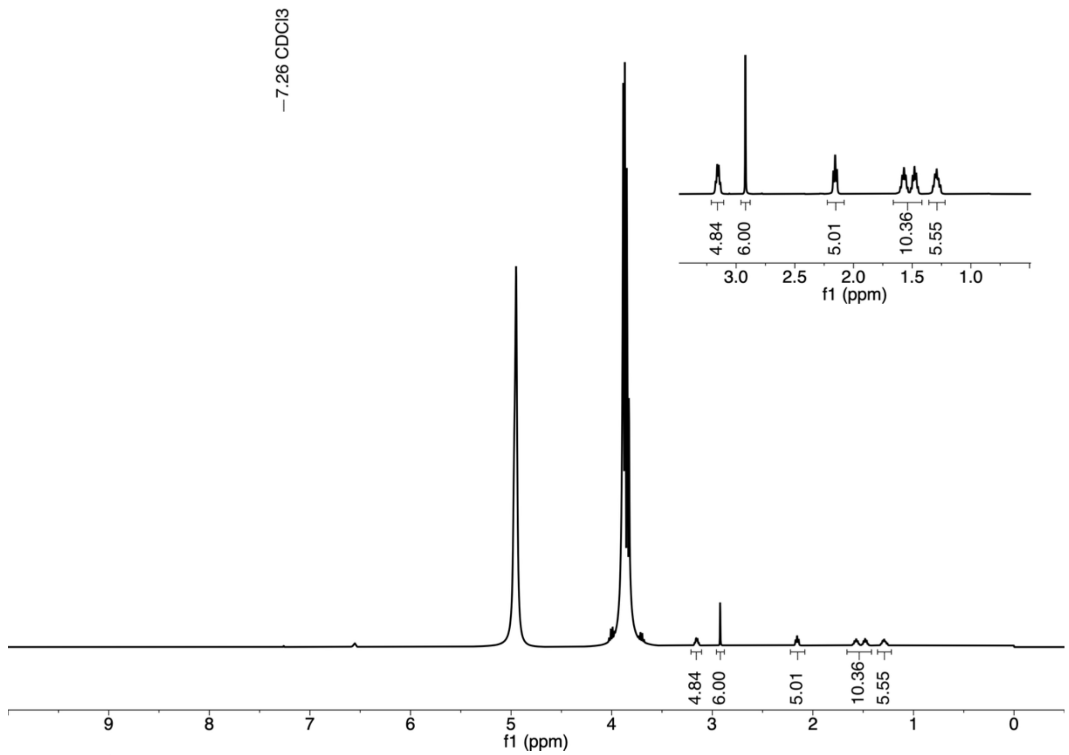

Figure S89. ^{1}H NMR of post-consumer PA66 multi-colored zip ties. Protonated TFE peaks appear at 3.8 ppm and 4.9 ppm. $DMSO_2$ signal appears at 2.9 ppm.

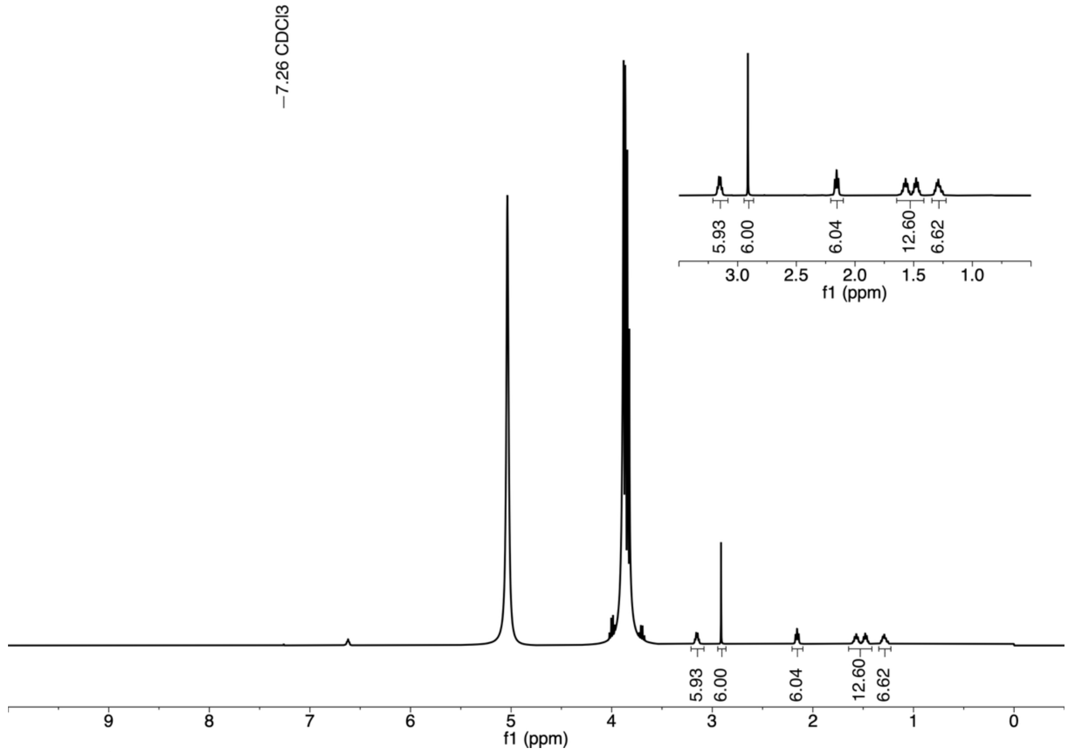

Figure S90. ^{1}H NMR of post-consumer PA66 red zip ties. Protonated TFE peaks appear at 3.8 ppm and 4.9 ppm. $DMSO_2$ signal appears at 2.9 ppm.

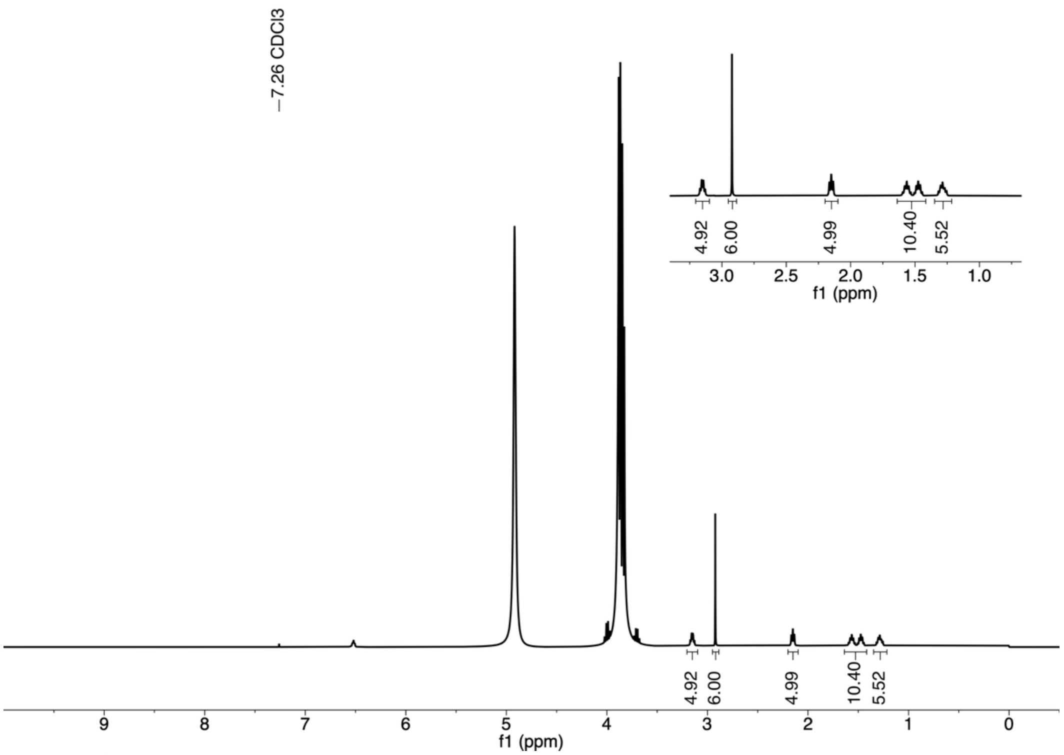

Figure S91. ^{1}H NMR of post-consumer PA66 green zip ties. Protonated TFE peaks appear at 3.8 ppm and 4.9 ppm. $DMSO_2$ signal appears at 2.9 ppm.

Post-Consumer PA66 Photothermal Acidic Hydrolysis

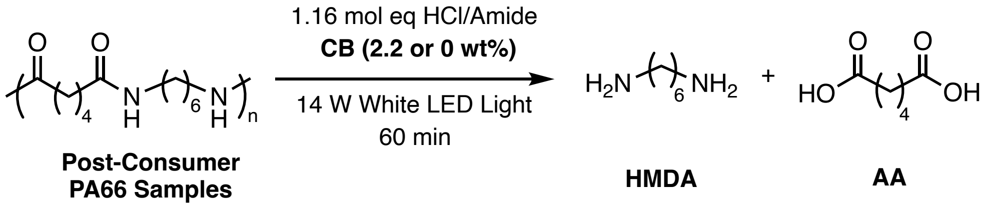

The procedure was modified from the general PA66 acidic hydrolysis depolymerization procedure, where post-consumer samples were used instead of purchased PA66 powder. All post-consumer samples were cut to approximately 1 to 2 mm pieces prior to depolymerization. Depolymerization reactions were performed with either 0 or 2.2 wt% CB. ^{1}H NMR analysis was performed in DMSO-d_6 and leftover PA66 was not quantified. The reaction workup was the same as the general PA66 photothermal acidic hydrolysis procedure. The depolymerization results are summarized below.

Table S36. Results of post-consumer PA66 photothermal acidic hydrolysis with 2.2 wt% CB.

Entry	PA66 Sample	PA66 Purity (%)	HMDA Yield$_{NMR}$(%)	AA Yield$_{NMR}$ (%)
1	PA66 Thread	81.4	>99	>99
2	PA66 Black Zip Ties	91.9	>99	>99
3	PA66 White Zip Ties	95.2	>99	>99
4	PA66 Mulitcolored Zip Ties	87.1	>99	>99
5	PA66 Red Zip Ties	92.8	>99	>99
6	PA66 Green Zip Ties	92.7	>99	>99

each entry is an average of 2 trials

Table S37. Results of post-consumer PA66 photothermal acidic hydrolysis with 0 wt% CB.

Entry	PA66 Sample	PA66 Purity (%)	HMDA Yield$_{NMR}$(%)	AA Yield$_{NMR}$ (%)
1	PA66 Thread	81.4	67.9	73.4
2	PA66 Black Zip Ties	91.9	>99	>99
3	PA66 White Zip Ties	95.2	0.0	0.0
4	PA66 Mulitcolored Zip Ties	87.1	71.2	71.7
5	PA66 Red Zip Ties	92.8	94.4	93.3
6	PA66 Green Zip Ties	92.7	65.4	66.6

each entry is an average of 2 trials

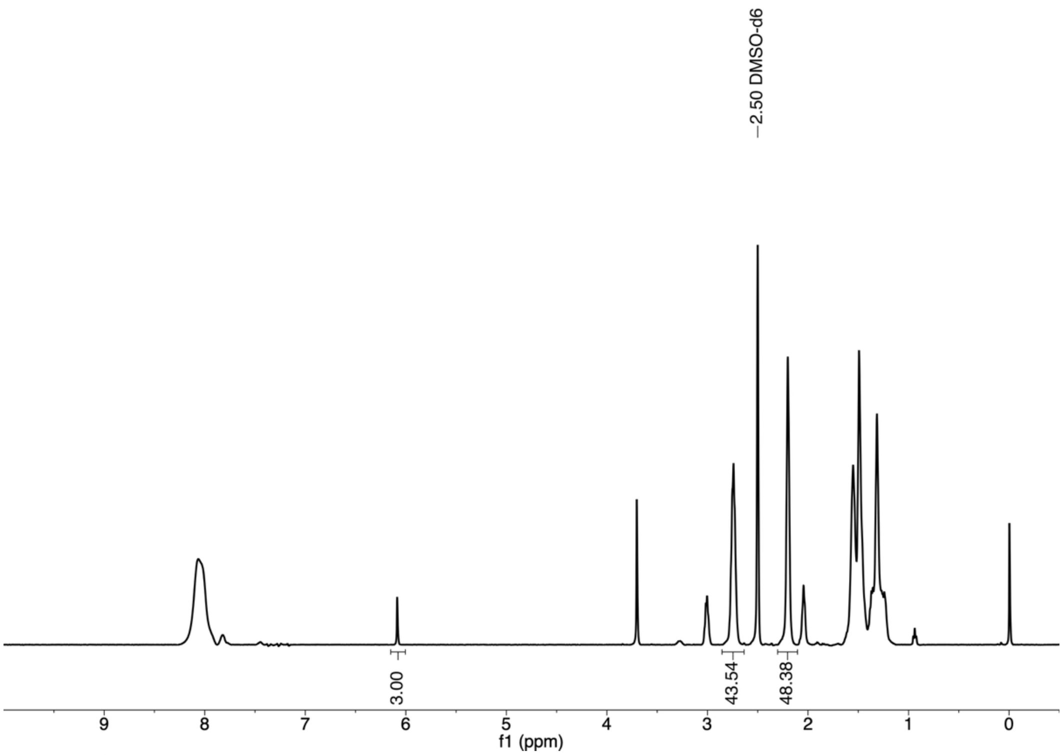

Figure S92. ^{1}H NMR of post-consumer PA66 thread after photothermal acidic hydrolysis with 2.2 wt% CB.

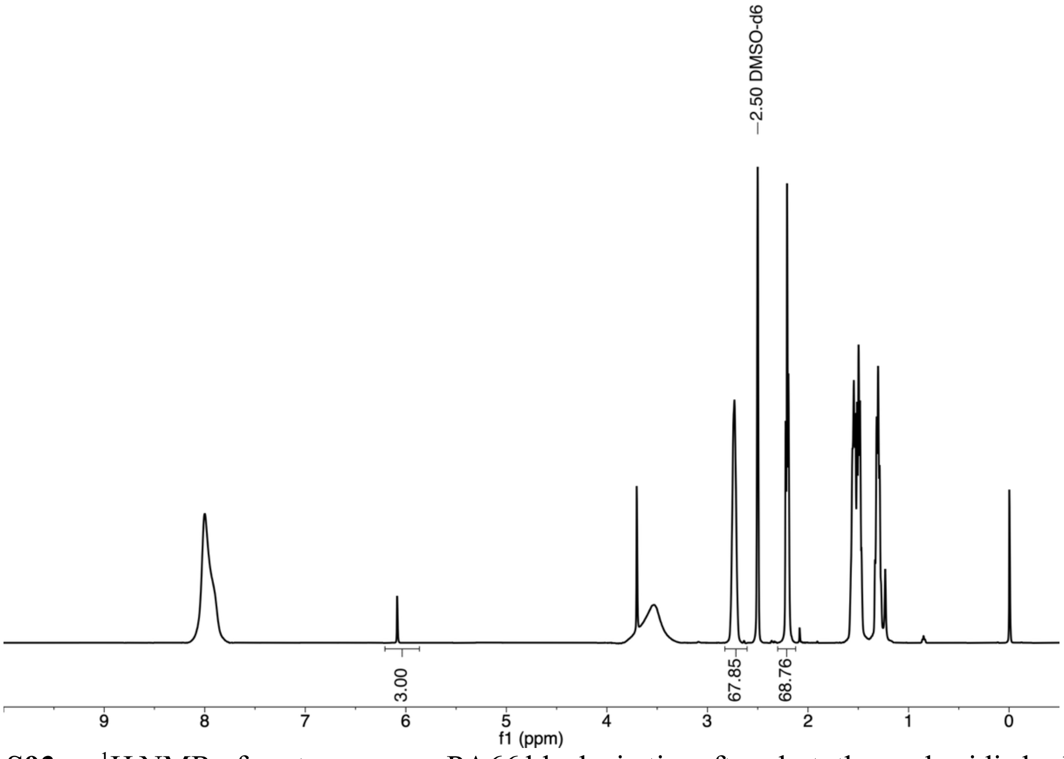

Figure S93. ^{1}H NMR of post-consumer PA66 black zip ties after photothermal acidic hydrolysis with 2.2 wt% CB.

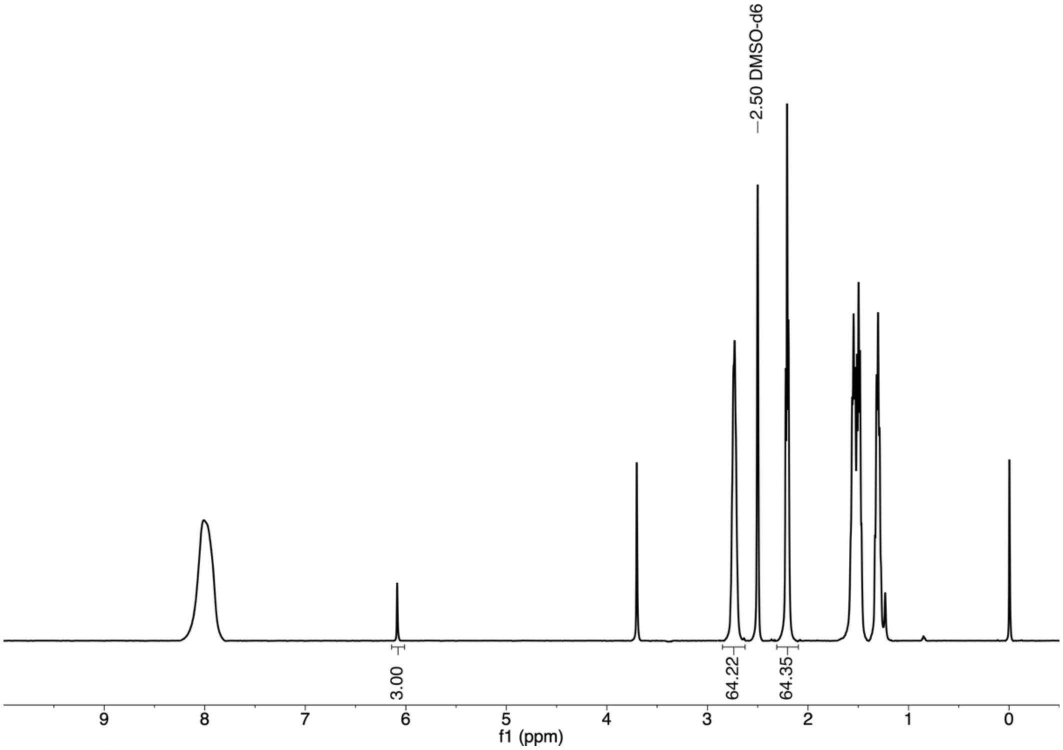

Figure S94. ^{1}H NMR of post-consumer PA66 white zip ties after photothermal acidic hydrolysis with 2.2 wt% CB.

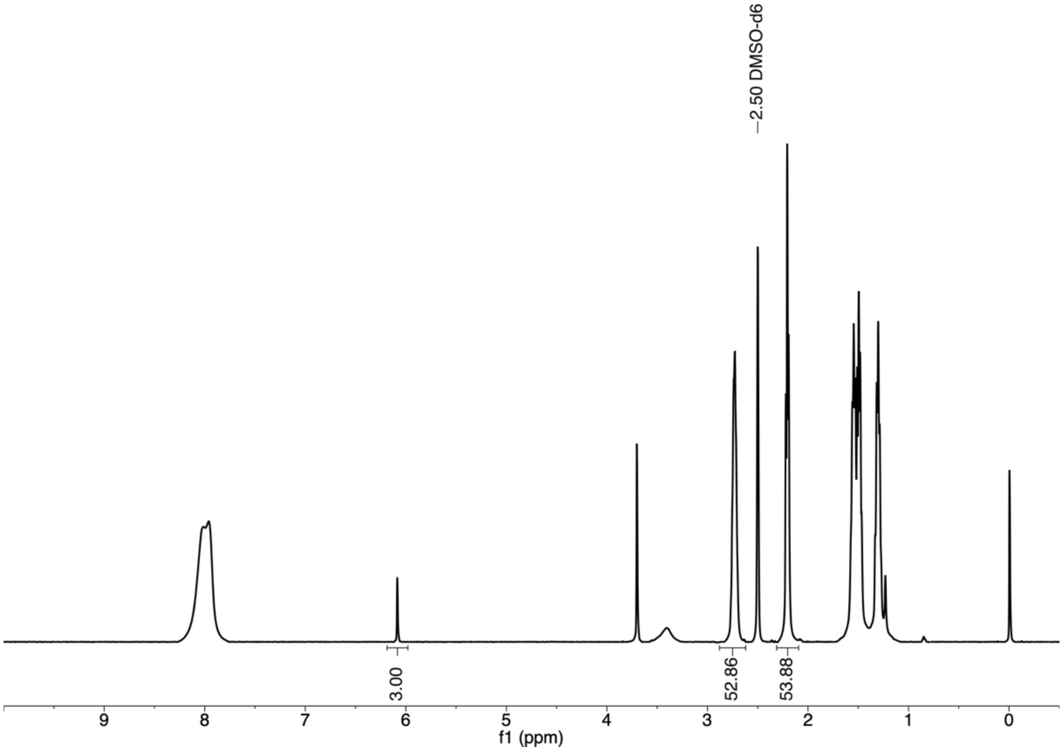

Figure S95. ^{1}H NMR of post-consumer PA66 multi-colored zip ties after photothermal acidic hydrolysis with 2.2 wt% CB.

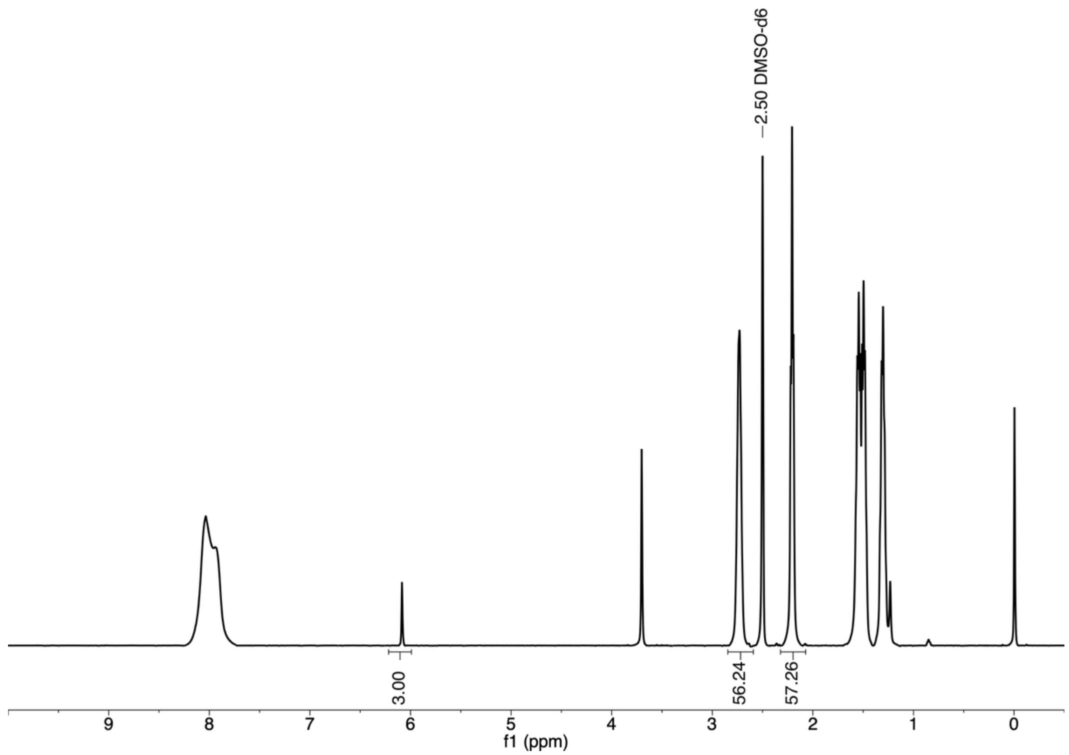

Figure S96. ^{1}H NMR of post-consumer PA66 red zip ties after photothermal acidic hydrolysis with 2.2 wt% CB.

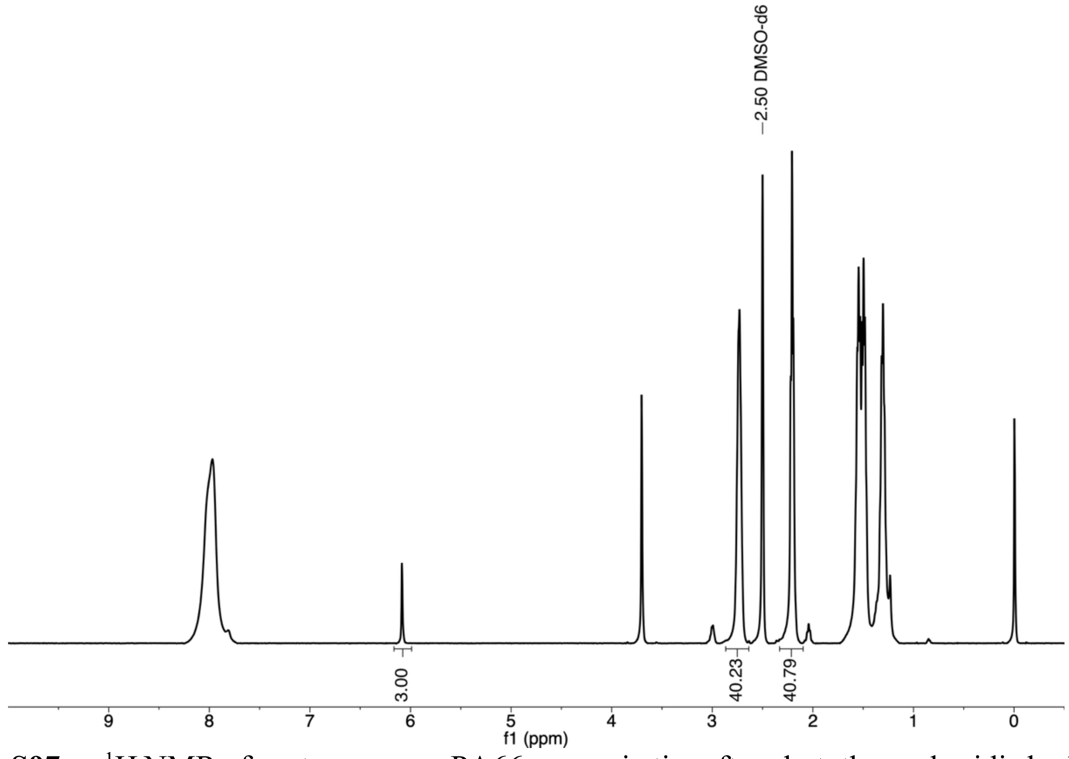

Figure S97. ^{1}H NMR of post-consumer PA66 green zip ties after photothermal acidic hydrolysis with 2.2 wt% CB.

Mixed Post-Consumer PA Photothermal Acidic Hydrolysis

Post-Consumer Mixed PA6 and PA66 Photothermal Acidic Hydrolysis

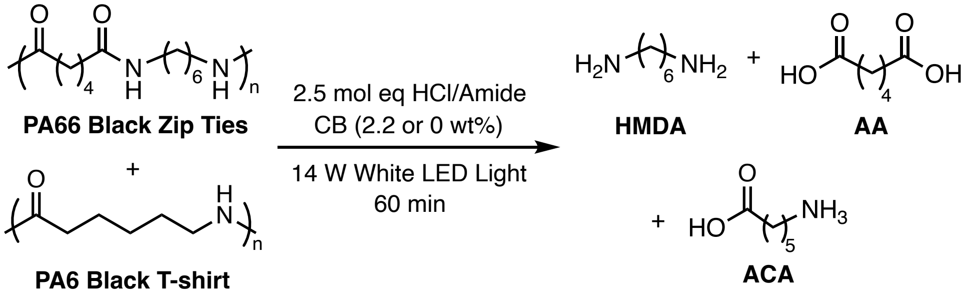

The procedure was slightly modified from the mixed PA6 and PA66 photothermal acidic hydrolysis procedure, where post-consumer PA6 T-shirt (50 mg) and post-consumer PA66 black zip ties (50 mg) were used as the starting material instead of PA6 and PA66. The copolymer filament was cut into small 1-mm pieces prior to hydrolysis. ^{1}H NMR analysis was performed in D_2O and leftover PA6 and PA66 were not quantified. The reaction workup was the same as the mixed PA6 and PA66 photothermal acidic hydrolysis procedure. The depolymerization results are summarized below.

Table S38. Results of post-consumer mixed PA6 and PA66 photothermal acidic hydrolysis.

Entry	CB (wt%)	PA6 Purity (%)	Total Monomer Recovery (%)
1	2.2	85.6	97.6 (± 0.8)
2	0	85.6	98.7 (± 0.3)

Each entry is an average of 3 trials. PA purity for mixed post-consumer samples is determined using the ratio of PA6 and PA66 starting material (in this case, 1:1 ratio of PA6 to PA66). All yields are scaled with PA purity.

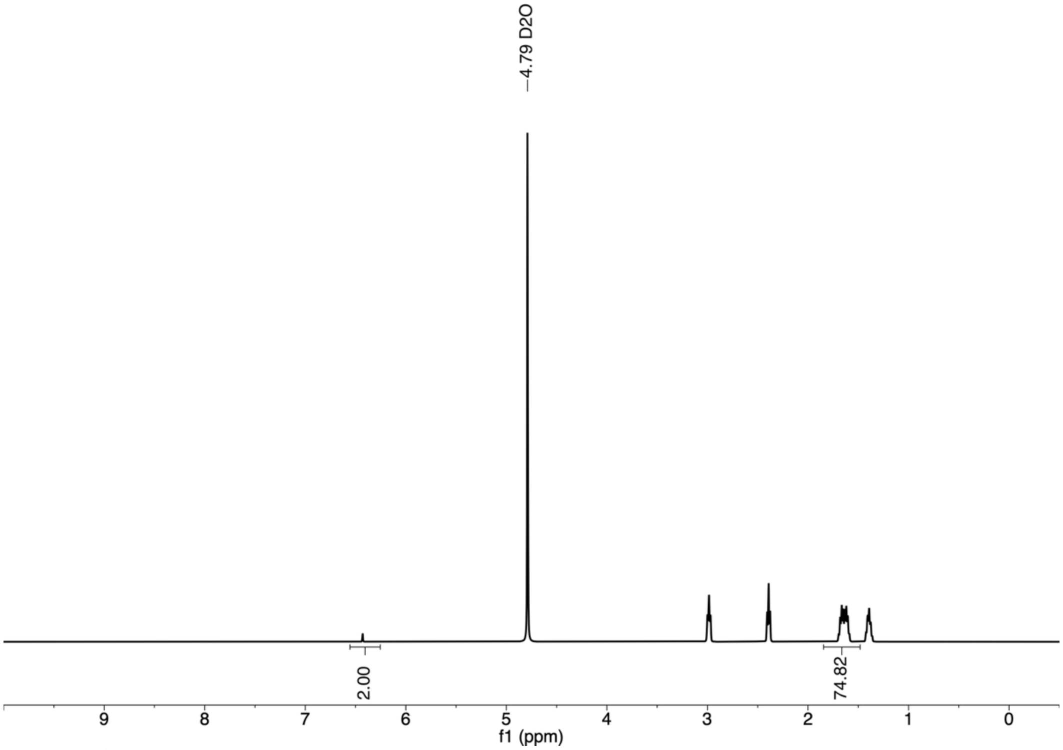

Figure S98. ^{1}H NMR of post-consumer PA6 t-shirt and PA66 black zip ties after photothermal acidic hydrolysis with 0 wt% CB.

PA6/66 Copolymer Filament Photothermal Acidic Hydrolysis

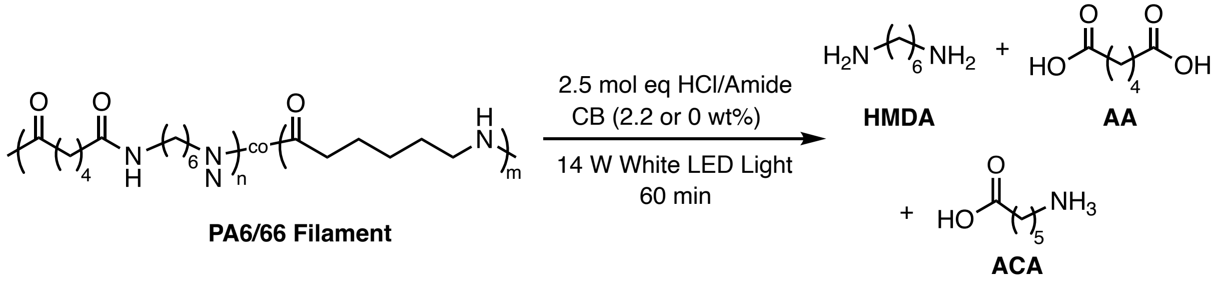

The procedure was slightly modified from the mixed PA6 and PA66 photothermal acidic hydrolysis procedure, where PA6/66 copolymer filament (100 mg) was used as the starting material instead of PA6 and PA66. The copolymer filament was cut into small 1-mm long pieces prior to hydrolysis. The PA6/66 copolymer filament purity was not determined as PA6 and PA66 have overlapping ^{1}H NMR signals, therefore the total monomer recovery was not scaled for PA purity. ^{1}H NMR analysis was performed in D_2O and leftover PA6/66 was not quantified. The reaction workup was the same as the mixed PA6 and PA66 photothermal acidic hydrolysis procedure. The ^{1}H NMR spectra and depolymerization results are summarized below.

Table S39. Results of PA6/66 filament copolymer photothermal acidic hydrolysis.

Entry	CB (wt%)	Total Monomer Recovery (%)
1	2.2	91.6 (± 1.4)
2	0	81.0 (± 3.5)

Each entry is an average of 3 trials. Yields are not scaled with purity.

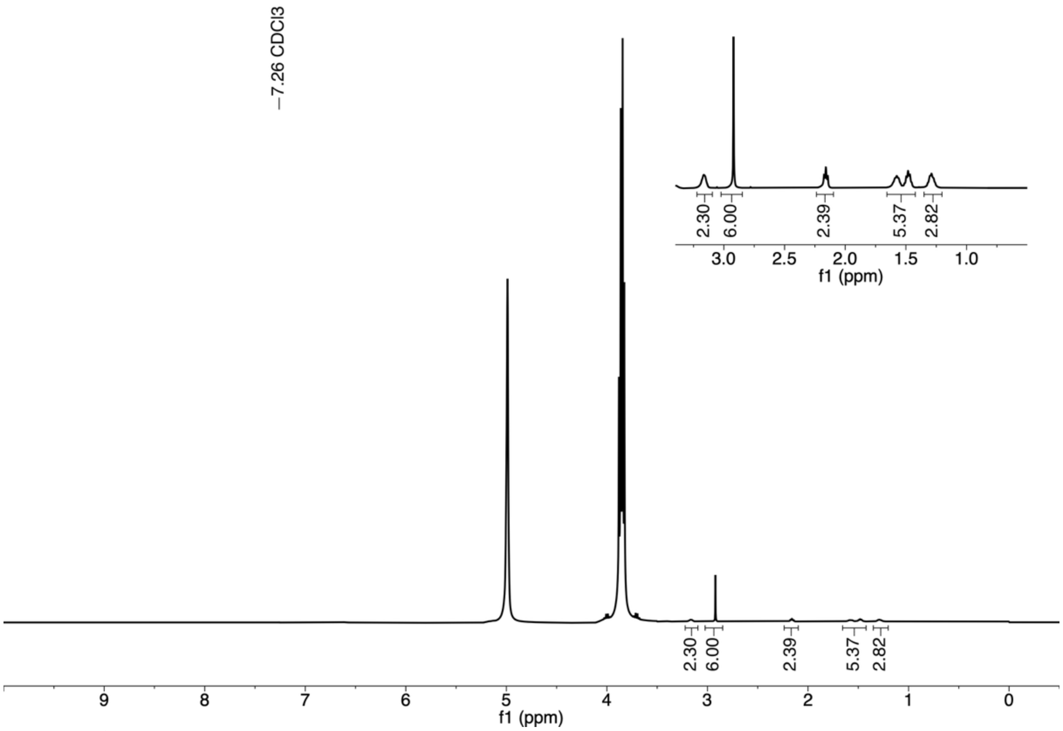

Figure S99. ^{1}H NMR of PA6/66 filament in 3:1 TFE/$CDCl_3$. The spectrum was baseline corrected between the 3.5 to 0.5 ppm region to ensure that protonated TFE signals appearing at 3.8 ppm and 4.9 ppm would not interfere with integrations. $DMSO_2$ signal appears at 2.92 ppm.

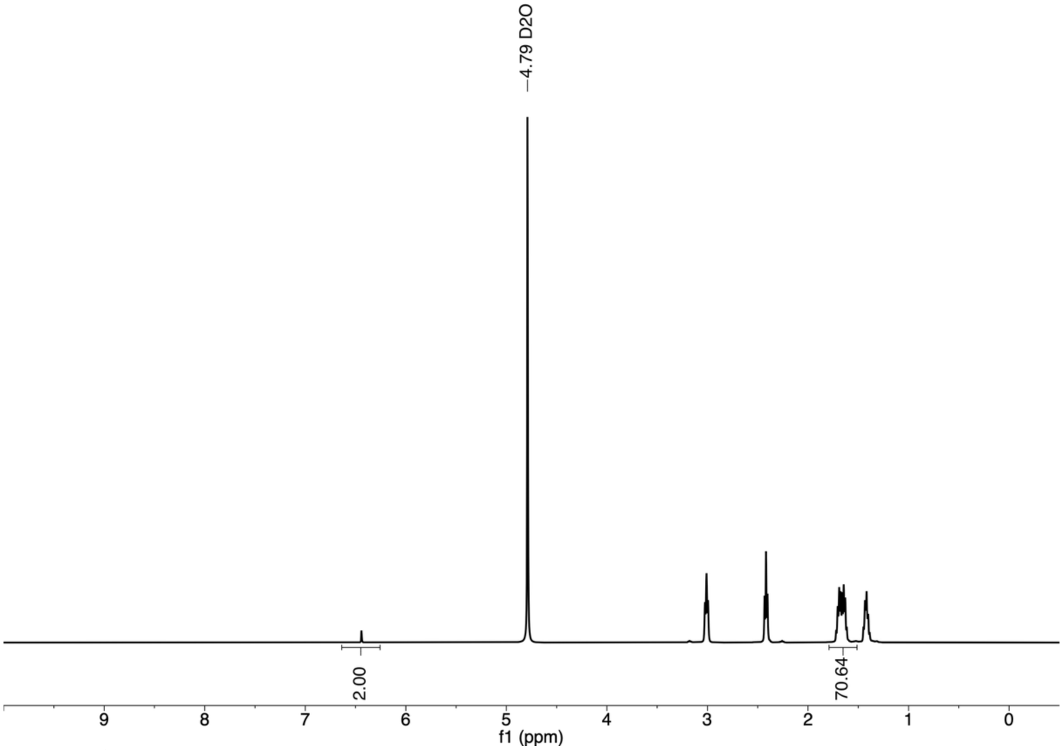

Figure S100. ^{1}H NMR of PA6/66 filament after photothermal acidic hydrolysis.

Polycondensation of HMDA, AA, and ACA from PA6/66 Copolymer Filament Photothermal Acidic Hydrolysis

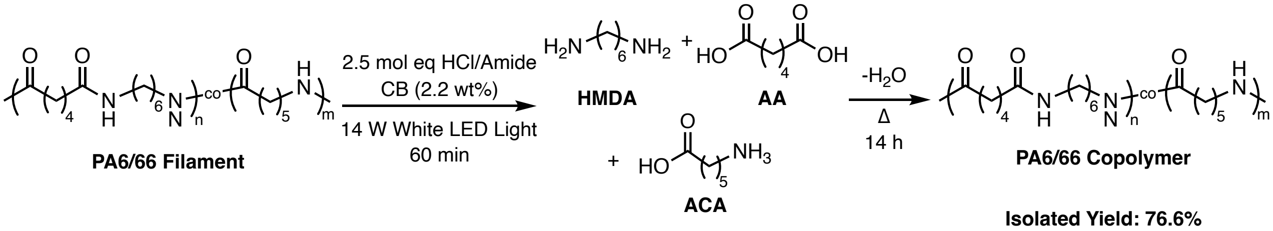

Polycondensation was performed to repolymerize monomers formed during the photothermal acidic hydrolysis of the PA6/66 copolymer filament. Using the same procedure outlined in the section entitled "Polycondensation of HMDA, AA, and ACA from Mixed PA6 and PA66 Photothermal Acidic Hydrolysis", a solid polymer product (76.6 mg, 76.6% isolated yield) was formed and characterized using ^{1}H and ^{13}C NMR analysis in 3:1 TFE/$CDCl_3$.

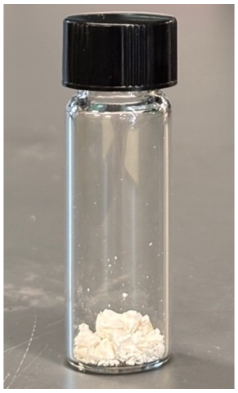

Figure S101. Synthesized PA6/66 copolymer *via* polycondensation of HMDA, AA, and ACA from PA6/66 copolymer filament photothermal hydrolysis.

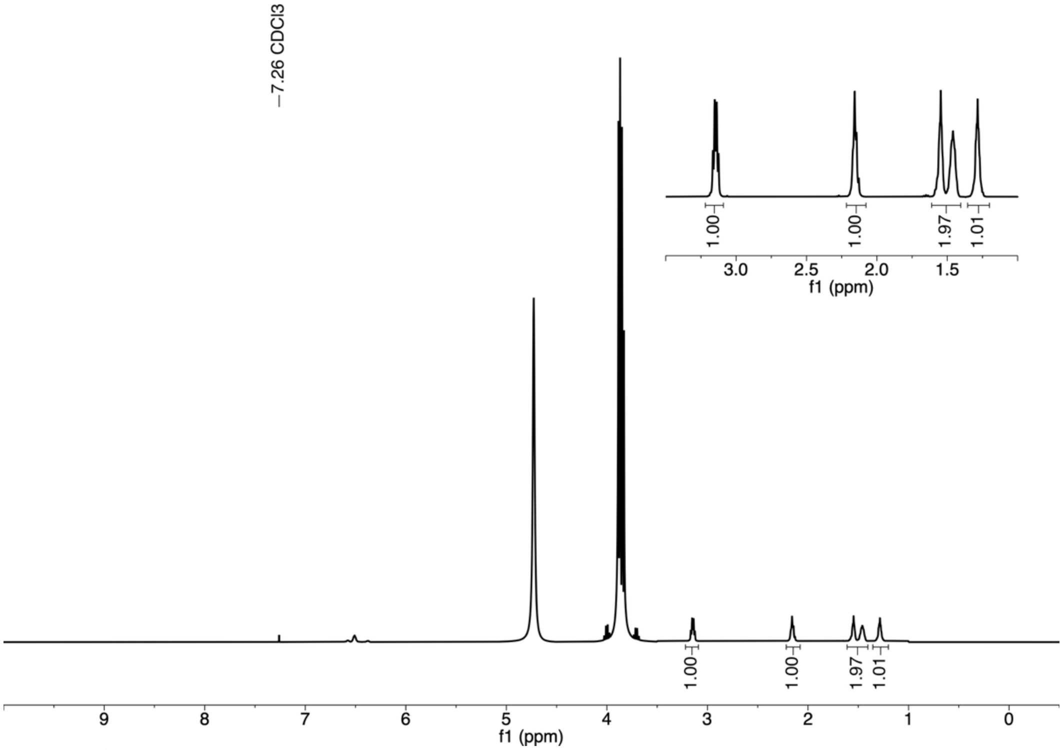

Figure S102. ^{1}H NMR of synthesized PA6/66 copolymer *via* polycondensation of HMDA, AA, and ACA from PA6/66 copolymer filament photothermal hydrolysis. The spectrum was baseline corrected between the 3.5 to 1.0 ppm region to ensure that protonated TFE signals appearing at 3.9 ppm and 4.9 ppm would not interfere with integrations.

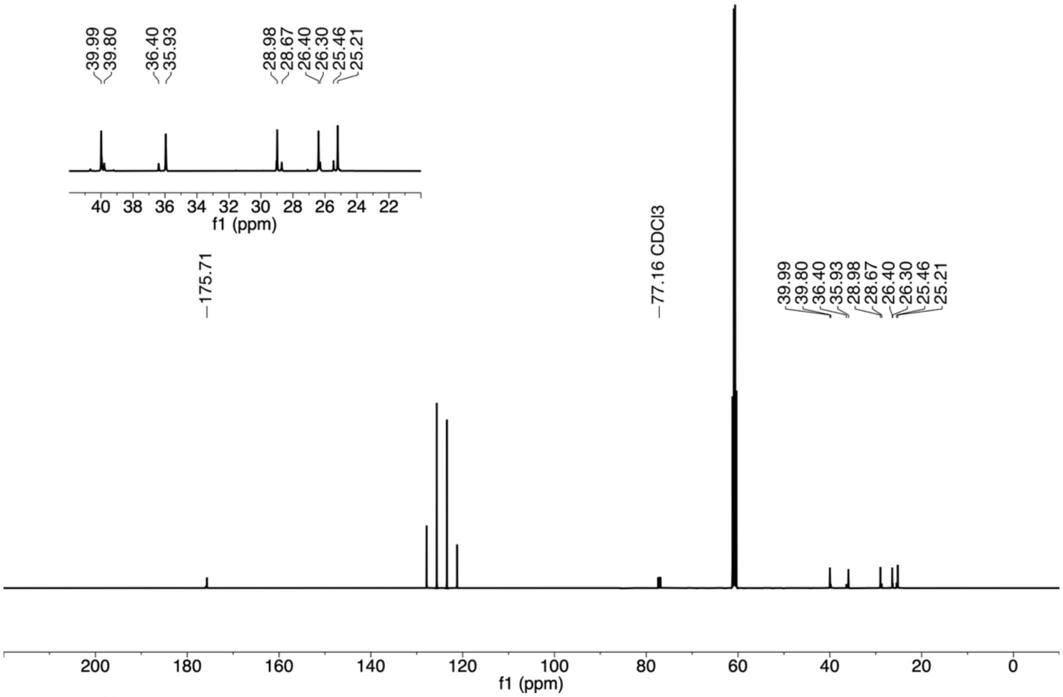

Figure S103. ^{13}C NMR of synthesized PA6/66 copolymer *via* polycondensation of HMDA, AA, and ACA from the PA6/66 copolymer filament photothermal hydrolysis. Protonated TFE peaks can be seen at 124 and 60 ppm.

Bulk Temperature Measurements of Photothermal Reactions

21 W Light Intensity Bulk Temperature Measurement Using a Digital Thermometer

To measure the bulk temperature under our photothermal ring-closing depolymerization conditions, a modified procedure from Oh et al, 2024 was performed.[5] CB (200 mg) was added to a 1 dram vial and sealed with a cap fitted with a punctured Teflon septum. A stainless-steel thermocouple attached to a digital thermometer (Digital K Type Thermometer, Gain Express) was placed inside the vial through the hole in the septum until the tip of the thermocouple was fully submerged in the solid contents of the vial. The vial was then placed 0.2 mm above a 6000K white LED light (21 W) and irradiated for 10 minutes. The temperature measured by the thermocouple was recorded every 15 seconds for the entire irradiation time. After the irradiation time finished, the thermocouple temperature was measured every 15 seconds for 7.5 minutes. The measurement results are summarized below.

Photothermal conversion creates localized temperature gradients near the photothermal agent. Therefore, it is important to note that measuring the bulk temperature is not a true reflection of the local temperatures near the surface of the photothermal agent, and this is supported using experimental data. The maximum bulk temperature measured in this experiment was 269.7 ºC (at t = 7 minutes). Thermal ring-closing reactions run at similar temperatures to this bulk temperature afford a lower amount of product compared to our optimized conditions. For example, thermal ring-closing reactions run at 300 ºC for 60 minutes affords 6.0% CPL yield (see section entitled "Thermal Controls for PA6 Photothermal Ring-Closing Depolymerization").

Thermal reactions run at 550 ºC for 10 minutes (67.3% CPL) provide comparable yields to our optimized photothermal conditions that use 21 W white light LED light (74.1% CPL) (see section entitled "General Procedure for PA6 Photothermal Ring-Closing Depolymerization"). Thus, it's likely that the temperatures achieved near the photothermal agent surface using 21 W white light LED light are above the bulk temperature that was measured in this experiment.

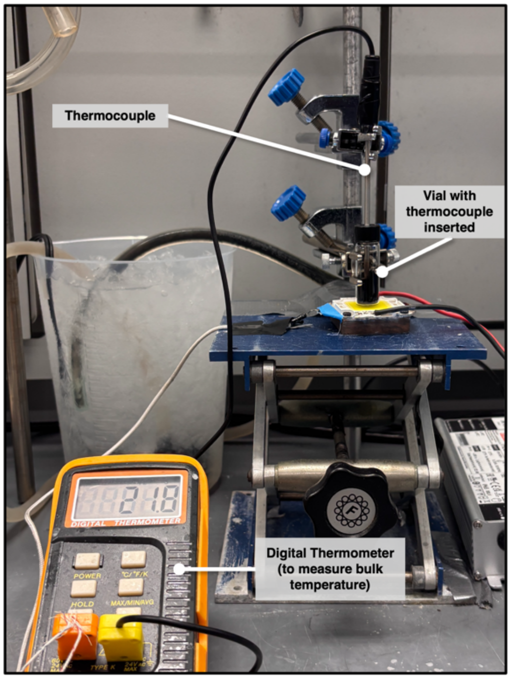

Figure S104. Bulk temperature measurement laboratory setup for 21 W white LED light.

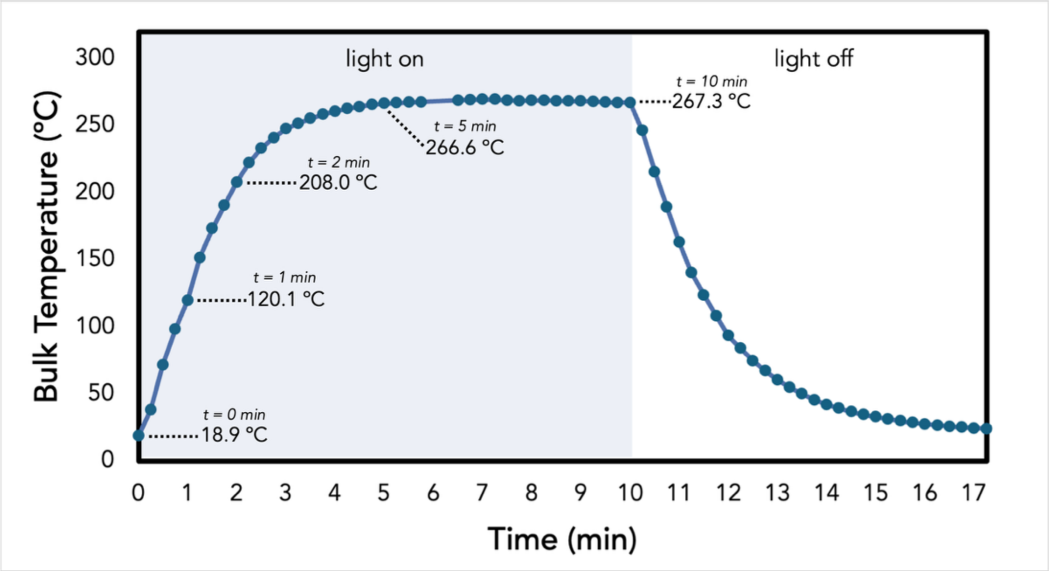

Figure S105. Plot of bulk temperature measurements using a 21 W white LED with 10 minutes of irradiation time. Temperature measurements were taken every 15 seconds using a digital thermocouple, with the exception of the 6.0-and 6.25-minute timepoints.

14 W Light Intensity Bulk Temperature Measurement Using a Thermal Camera

As HCl could cause corrosion of a digital thermocouple if inserted inside the reaction vial, we opted to use the FLIR thermal camera to measure the bulk temperature of the photothermal acidic hydrolysis reaction. FLIR TG297 industrial high temperature thermal camera from Teledyne FLIR was used to measure the bulk temperature under our photothermal acidic hydrolysis conditions. The procedure was the same as the general PA66 acidic hydrolysis depolymerization procedure, where FLIR photos were taken every 30 seconds for the first 2.5 minutes of light irradiation, then at the 5-, 10-, and 15-minute timepoint. The temperature was not measured beyond the 15-minute timepoint. The thermal photos are shown below.

As noted in the previous section, it is important to note that the bulk temperature measurements are not indicative of the local surface temperature near the photothermal agent surface.

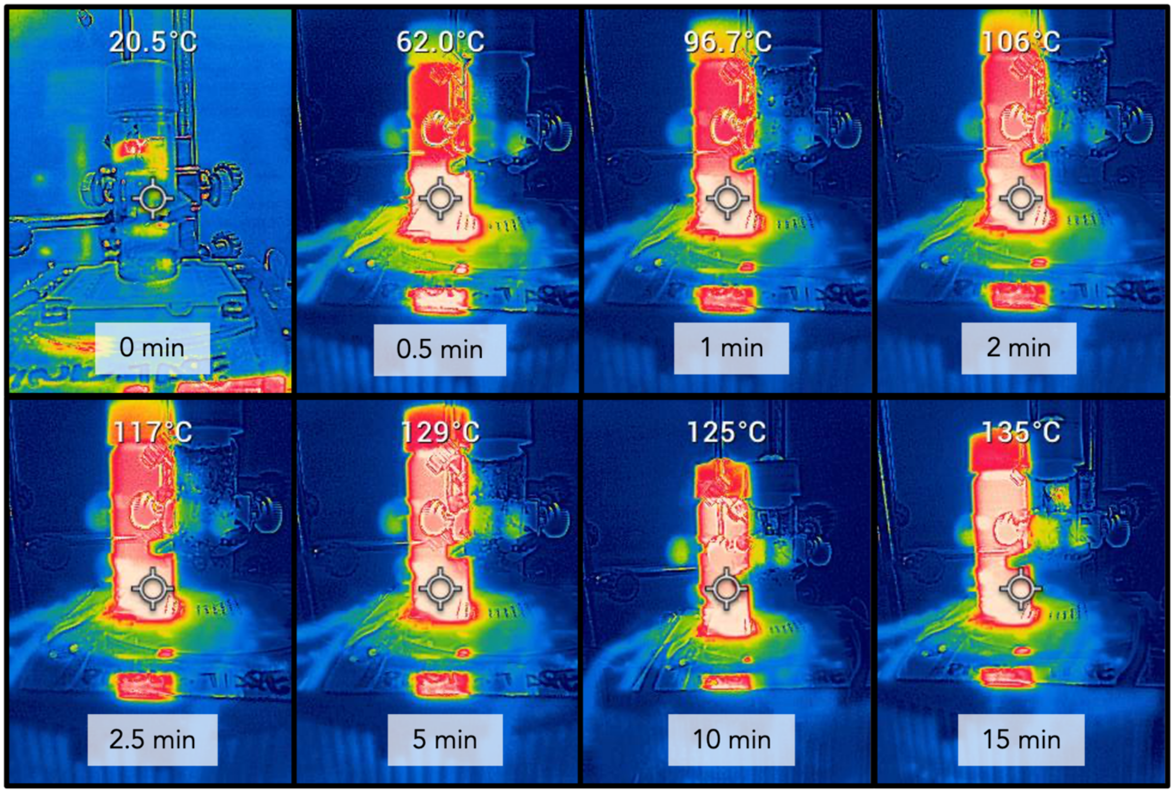

Figure S106. Thermal camera photos of the PA66 acidic hydrolysis reaction vial under 14 W white LED light irradiation.

Photothermal Ring-Closing Depolymerization for PA11 and PA12

Photothermal ring-closing depolymerization was applied to PA11 and PA12 to form the cyclic monomers undecalactam and laurolactam, respectively.

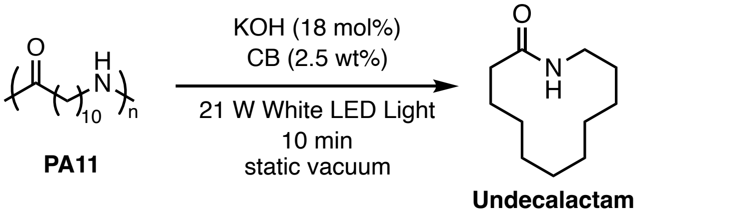

Undecalactam Yield$_{NMR}$: 9.3% (± 4.8)

(average of 3 trials)

For PA11 ring-closing depolymerization, PA11 (79.9 mg, 0.436 mmol), KOH (4.4 mg, 0.078 mmol, 0.18 mol equivalents), and CB (2.2 mg, 2.5 wt% of total reaction volume) were added, sealed with a cap fitted with a Teflon septum, and vortexed for 1 minute to mix until thoroughly combined. The vial was evacuated and backfilled with nitrogen three times and left under static vacuum after a fourth vacuum pull. The vial was subsequently placed 0.2 mm above a 6000K white LED light (21 W) and irradiated for 10 minutes. After the reaction, the vial was cooled in a dry ice-acetone bath for 1 minute and defrosted to room temperature. $CDCl_3$ (1 mL) and a stock solution of 1,3,5-trimethoxybenzene stock solution (0.2 mL, 0.12M in $CDCl_3$) were added to the reaction vial. Aliquots were taken for ^{1}H NMR analysis (0.1 mL of the dissolved reaction mixture was diluted with 0.35 mL $CDCl_3$), and the spectrum is shown below.

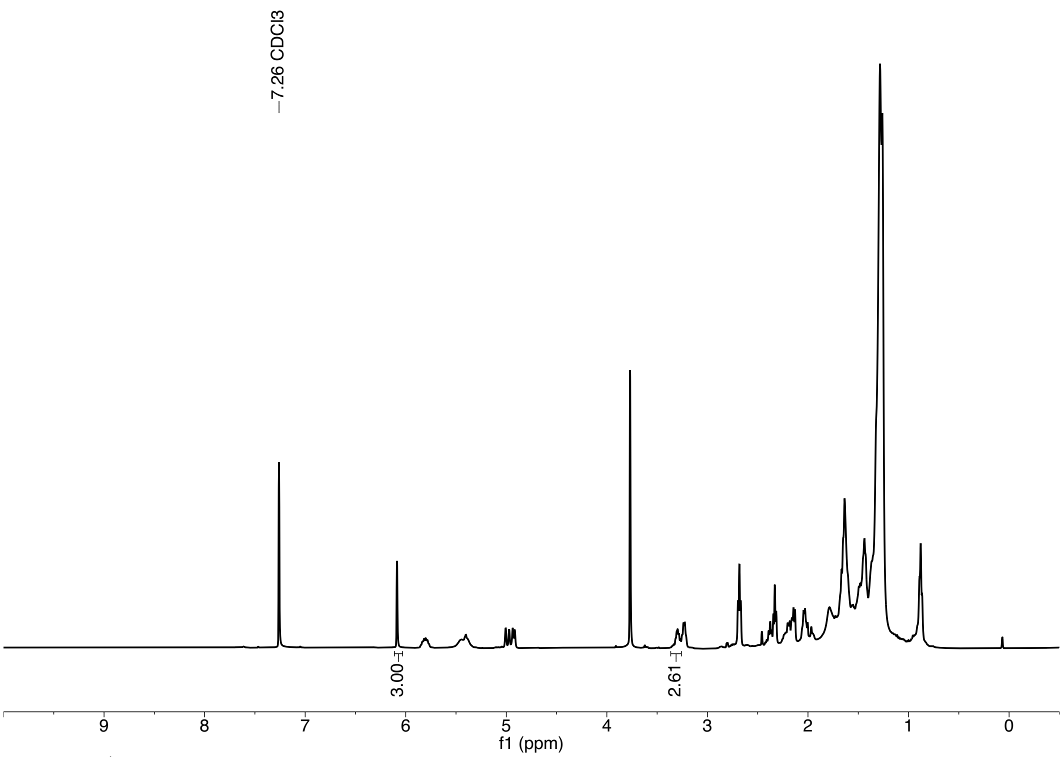

Figure S107. ^{1}H NMR of PA11 after photothermal ring-closing depolymerization.

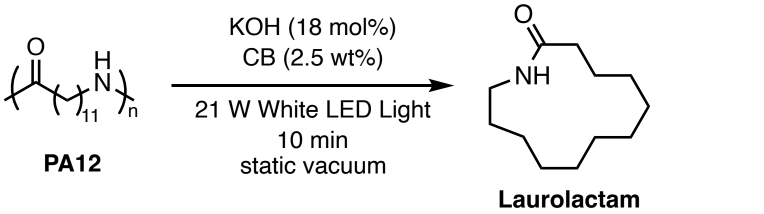

Laurolactam Yield$_{NMR}$: 6.9% (± 1.1)

(average of 3 trials)

For PA12 ring-closing depolymerization, PA12 (80.2 mg, 0.406 mmol), KOH (4.1 mg, 0.073 mmol, 0.18 mol equivalents), and CB (2.2 mg, 2.5 wt% of total reaction volume) were added, sealed with a cap fitted with a Teflon septum, and vortexed for 1 minute to mix until thoroughly combined. The vial was evacuated and backfilled with nitrogen three times and left under static vacuum after a fourth vacuum pull. The vial was subsequently placed 0.2 mm above a 6000K white LED light (21 W) and irradiated for 10 minutes. After the reaction, the vial was cooled in a dry ice-acetone bath for 1 minute and defrosted to room temperature. $CDCl_3$ (1 mL) and a stock solution of 1,3,5-trimethoxybenzene stock solution (0.2 mL, 0.12M in $CDCl_3$) were added to the reaction vial. Aliquots were taken for ^1H NMR analysis (0.1 mL of the dissolved reaction mixture was diluted with 0.35 mL $CDCl_3$), and the spectrum is shown below.

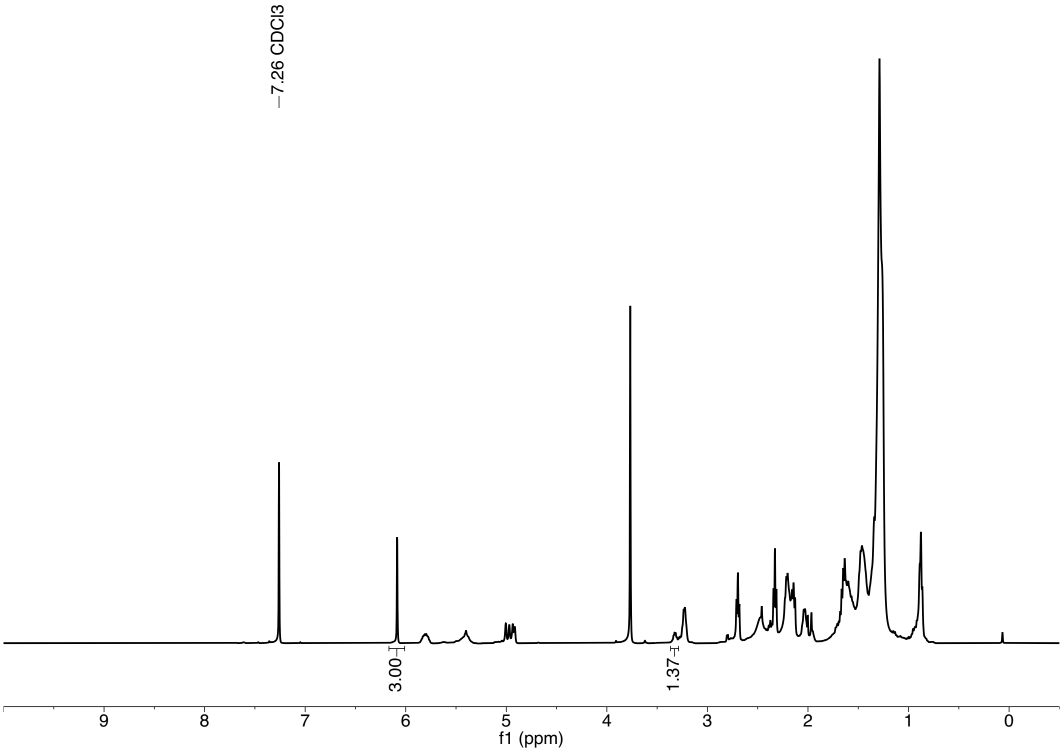

Figure S108. ^1H NMR of PA12 after photothermal ring-closing depolymerization.

References

(1) Peng, L.; Li, J.; Peng, S.; Yi, C.; Jiang, F. The Crystal-Form Transition Behaviours and Morphology Changes in a Polyamide 6 Cyclic Dimer. *R. Soc. Open Sci.* **2018**, *5* (11), 180957. https://doi.org/10.1098/rsos.180957.

(2) Carraher, C. E. Synthesis of Caprolactam and Nylon 6. *J. Chem. Educ.* **1978**, *55* (1), 51. https://doi.org/10.1021/ed055p51.

(3) Jiang, M.; Tan, H.; Jiang, L.; Duan, J.; Gu, X.; Feng, L.; Zhang, C. Anionic Ring-Opening Polymerization of ε-Caprolactam above the Crystallization Temperature of Polyamide 6: A Study on the Effects of the Activator and Catalyst. *Ind. Eng. Chem. Res.* **2024**, *63* (45), 19476–19485. https://doi.org/10.1021/acs.iecr.4c02855.

(4) Lehrle, R. S.; Parsons, I. W.; Rollinson, M. Thermal Degradation Mechanisms of Nylon 6 Deduced from Kinetic Studies by Pyrolysis-g.c. *Polym. Degrad. Stab.* **2000**, *67* (1), 21–33. https://doi.org/10.1016/S0141-3910(99)00112-3.

(5) Oh, S.; Jiang, H.; Kugelmass, L. H.; Stache, E. E. Recycling of Post-Consumer Waste Polystyrene Using Commercial Plastic Additives. *ACS Cent. Sci.* **2024**, acscentsci.4c01317. https://doi.org/10.1021/acscentsci.4c01317.

(6) Herrera, M.; Matuschek, G.; Kettrup, A. Main Products and Kinetics of the Thermal Degradation of Polyamides. *Chemosphere* **2001**, *42* (5–7), 601–607. https://doi.org/10.1016/S0045-6535(00)00233-2.

(7) *Safety Assessment of P-Phenylenediamine, p-Phenylenediamine HCl, and p-Phenylenediamine Sulfate as Used in Cosmetics*; Cosmetic Ingredient Review.

(8) Davis, R. D.; Jarrett, W. L.; Mathias, L. J. Solution 13 C NMR Spectroscopy of Polyamide Homopolymers (Nylons 6, 11, 12, 66, 69, 610 and 612) and Several Commercial Copolymers. *Polymer* **2001**, *42* (6), 2621–2626. https://doi.org/10.1016/S0032-3861(00)00501-2.
